# The global burden of migraine and tension-type headache, 1990–2021: a multi-model Bayesian meta-regression with decomposition of age-period-cohort effects, health inequalities, and frontier analysis

**DOI:** 10.3389/fneur.2026.1818932

**Published:** 2026-08-18

**Authors:** Jinpin Zhang, Yuanxi Xia, Xuan Wen, Qinghua Huang, Qian Zhang, Yanjun Di, Miaomiao Nie, Yong Diao, Xunxun Wu, Xiaoyun You, Huiyong Yang, Dandan Tong, Fengjing Cai

**Affiliations:** 1 School of Medicine, Huaqiao University, Quanzhou, China; 2 Department of Neurosurgery, The Second Affiliated Hospital of Fujian Medical University, Quanzhou, Fujian, China

**Keywords:** GBD, incidence, migraine, prevalence, tension-type headache, YLD

## Abstract

**Background:**

The Global Burden of Disease Study 2021 (GBD 2021) indicates that headache disorders constitute a major global public health burden. Based on its data, this study constructed models of migraine and tension-type headache across 204 countries and 21 regions to systematically analyze their epidemiological characteristics.

**Methods:**

The analysis stratified prevalence by age, sex, and Socio-demographic Index (SDI), and applied decomposition methods to quantify the attributable burden. Age–Period–Cohort models and spatiotemporal models of health inequality were constructed to assess burden disparities. BAPC models were employed to project trends over the next decade, with 95% uncertainty intervals generated through Monte Carlo simulation.

**Results:**

In 2021, the global prevalence of the two types of headache remained largely unchanged compared to 1990, but years lived with disability (YLDs) increased significantly, with the heaviest burden observed among young and middle-aged women. Migraine was most prevalent in Tropical Latin America and Western Europe, while tension-type headache was highest in High-income North America; the fastest increases were observed in the Andean Latin America and East Asia regions. Notable increases occurred in Singapore and China, whereas Thailand showed a significant decline. The burden was strongly positively correlated with the SDI, and the suppressive effect of aging on migraine showed gender-specific differences.

**Conclusion:**

This study is the first to integrate age-period-cohort analysis, health inequality decomposition, frontier analysis, and multi-model prediction to quantify the drivers of migraine and TTH burden up to 2021, addressing key evidence gaps left by prior GBD studies.

## Background

In 2021, neurological disorders became the second most prevalent disease category globally, after other infectious diseases, with headache disorders accounting for 97.8% (95% UI: 97.6–98.1%). This study focuses primarily on two major types of headache disorders: migraine and tension-type headache. Diagnostic criteria strictly follow the International Classification of Headache Disorders, 3rd edition (ICHD-3), with specific diagnostic criteria applied for migraine and tension-type headache, respectively (1, 2).

Migraine is a common, chronic, and disabling neurological disorder involving a complex interplay of neurovascular, genetic, and inflammatory mechanisms, with increased excitability of the central nervous system being one of its core pathological features. It is typically characterized by recurrent headache attacks accompanied by associated symptoms, including aura in some cases. A small proportion of patients may even present with monogenic mutations (3, 4). As the most common type of primary headache, tension-type headache (TTH) is a multifactorial disorder involving genetic and environmental factors, with a global lifetime prevalence approaching 50%. While tension-type headache contributes considerably to global disability burden, migraine remains the leading cause of headache-related disability worldwide. Clinically, migraine and TTH exhibit some differences. The lifetime risk of developing migraine in the general population is approximately 12%, and about one-third of migraine patients experience visual aura during attacks. Accordingly, migraine is classified into three main types: migraine without aura, migraine with aura, and chronic migraine, with the former being the most prevalent. Migraine is typically characterized by recurrent unilateral, moderate-to-severe throbbing headaches, often accompanied by nausea, vomiting, and multisensory hypersensitivity (5). Tension-type headache (TTH) can be classified into three subtypes: infrequent, frequent, and chronic. It is characterized by recurrent mild to moderate headaches, typically presenting as bilateral pressure or dull pain with a “band-like” tightening sensation, and the pain location is not fixed. Unlike migraine with aura, TTH is usually not accompanied by photophobia, phonophobia, or nausea (6). It is worth noting that GBD 2021 does not distinguish between migraine with and without aura, and most epidemiological studies report only overall migraine data. Therefore, this article focuses on the general characteristics of migraine and tension-type headache.

Considering the close association between disease burden and socioeconomic development levels, the GBD study introduced the Socio-demographic Index (SDI) as a comprehensive evaluation metric (2). The Socio-demographic Index (SDI) consists of three dimensions: per capita lagged income, average years of education for the population aged 15 and above, and total fertility rate for women under 25 years old. Its range is from 0.00 to 1.00, commonly multiplied by 100 for representation. The GBD database classifies countries and regions into five levels based on global development in 2021: low (<0.46), lower-middle (0.46–0.60), middle (0.61–0.69), upper-middle (0.70–0.81), and high (>0.81) (7). As the GBD database is publicly available aggregate data and the information is non-identifiable, ethical approval and informed consent are not required for this study.

To develop effective strategies for improving patient health, a systematic assessment of the disease burden of migraine and tension-type headache is crucial. However, significant gaps in existing research severely hinder strategy development: Although several studies have assessed the global burden of migraine and tension-type headache using Global Burden of Disease (GBD) data (8), most were updated only to 2016 and did not establish a comprehensive multi-model analytical framework including age-period-cohort (APC) modeling, inequality decomposition, and frontier analysis. Between 2016 and 2021, the world experienced major public health events, such as the COVID-19 pandemic, which may have had a potential impact on the epidemiological characteristics of different headache types. Most importantly, there are widespread methodological issues in current research, including a single temporal perspective, fragmented health disparities, and poor integration of indicators. These gaps in methodology have hindered the development of a comprehensive evidence system, highlighting the lack of theoretical depth and methodological innovation. In response to these challenges, this study innovatively integrates the Age-Period-Cohort model (APC), health inequality analysis, frontier analysis, and decomposition models to analyze epidemiological trends, along with Bayesian hierarchical regression, ARIMA, and Holt-Winters models for time series forecasting. This study is the first to conduct multi-dimensional modeling and analysis of the epidemiological characteristics of migraine and tension-type headache in 204 countries and 21 regions worldwide from 1990 to 2021. Using advanced methods such as APC effect three-dimensional surface plots, Bayesian posterior distribution visualization, and health inequality contribution decomposition charts, we systematically reveal the heterogeneous distribution characteristics of prevalence, incidence, and disability burden across etiological, demographic, and social development dimensions (8).

## Methods

GBD 2021 is a globally recognized disease burden assessment system that updates and evaluates age- and sex-specific epidemiological data for 371 diseases and 88 risk factors across 204 countries and 21 regions. The data for this study were derived from the GBD 1990–2021 estimates, with key indicators including incidence rate, prevalence rate, YLD. These indicators can be expressed in multiple forms, such as counts, rates, or percentages.

For data presentation, age-standardized prevalence rates and YLD are expressed per 100,000 people, with 95% uncertainty intervals (UIs). Notably, in the GBD assessment system, the disease burden is measured using disability-adjusted life years (DALYs), which is the sum of years of life lost (YLLs) due to premature death and YLD due to disability. Since the GBD does not classify headache disorders as a primary cause of death, the YLL was set to 0, meaning DALYs were equivalent to YLD (8, 9).

To address different epidemiological questions, we used complementary analytical approaches rather than multiple interchangeable models. First, descriptive analyses were used to characterize the burden of migraine and tension-type headache (TTH) according to age, sex, region, and Socio-demographic Index (SDI). Second, Joinpoint regression was applied to quantify long-term temporal changes in age-standardized prevalence and YLD rates from 1990 to 2021 by estimating annual percentage change (APC) and average annual percentage change (AAPC) with 95% confidence intervals (CIs). Third, decomposition analysis was used to partition changes in burden into contributions from population growth, population aging, and epidemiological change. Fourth, the age-period-cohort (APC) model was used to identify temporal patterns associated with age, period, and birth cohort effects. Finally, the Bayesian age-period-cohort (BAPC) model was used as the primary approach for forecasting, whereas ARIMA and Holt-Winters models were included as conventional time-series comparators to assess the consistency of projected trends.

Joinpoint regression was based on a log-linear model and used Monte Carlo permutation tests to determine the optimal number of joinpoints, with a maximum of six joinpoints and a minimum of zero. If the 95% CI of the APC did not include 0, an APC > 0 was interpreted as an increasing trend, whereas an APC < 0 indicated a decreasing trend (10).

Decomposition analysis was used to quantify the relative contributions of population growth, population aging, and epidemiological change to the observed changes in disease burden over time, thereby helping to identify the main drivers of burden variation.

The APC model was used to disentangle temporal patterns that could not be captured by overall trend analysis alone. By simultaneously considering age, period, and birth cohort dimensions, this model estimated net drift and local drift and described how burden patterns varied across successive generations and calendar periods (11). To meet the requirements of the APC framework, data were arranged into consecutive 5-year age groups from 5–9 years to 95 years and older, consecutive 5-year calendar periods from 1990 to 2021, and corresponding birth cohorts derived from age and period groupings. Because this study was based on global GBD data, all analyses were conducted at the global, regional, or national level as appropriate. It should be noted that APC models are inherently descriptive and are intended to characterize temporal patterns rather than to establish causal relationships.

To assess health inequality, incidence rates were ranked by SDI, and regression lines were fitted using the cumulative population distribution midpoint as the independent variable. The slope index was used to reflect the absolute disparity across SDI groups (12). In addition, frontier analysis was performed to estimate the distance between each country’s age-standardized YLD rate and the theoretical minimum level expected at a given SDI, thereby identifying locations with potentially excess burden relative to their development level (13). LOESS smoothing with different spans (0.3, 0.4, and 0.5) was used to characterize the nonlinear relationship between SDI and age-standardized YLD rates.

For forecasting, we used the R packages “BAPC” and “INLA” (Integrated Nested Laplace Approximation) to build the BAPC model based on GBD data from 1990 to 2021. This model was used to project age-standardized incidence and YLD rates of migraine and TTH from 2022 to 2030 while accounting for age, period, and cohort structures (14). Compared with traditional Markov Chain Monte Carlo methods, the BAPC approach offers greater computational efficiency and stable estimation in hierarchical Bayesian settings (12).

To complement the BAPC projections, we also applied ARIMA and Holt-Winters models as conventional time-series forecasting approaches. ARIMA models were fitted using the auto.arima() function, and model fit was evaluated using the Akaike Information Criterion (AIC). Holt-Winters exponential smoothing was used to capture level and trend components and to reduce short-term random fluctuations (15). These two approaches were not intended to replace the BAPC model, but rather to provide additional reference points for the direction and consistency of projected trends. Because migraine and TTH showed consistently higher burden in females than in males, forecasting analyses were additionally presented for females.

All data analyses were conducted using the World Health Organization’s Health Equity Assessment Toolkit, and visualization work was completed using the statistical computing software R (version 4.4.1). This systematic study provides in-depth insights into the global epidemiological characteristics of migraine and TTH, including comparisons and trends of disease burden across global regions in 2021, differences in burden by sex and age, and disease burden trends across 204 countries and 21 regions based on SDI changes. These findings offer valuable scientific evidence for the prevention and control of migraine and TTH worldwide.

## Results

### Global level

To answer the overall burden changes of migraine and tension-type headache globally, we first estimated the prevalence and YLDs across 204 countries and 21 regions from 1990 to 2021. In 2021, there were 1.158 billion (95% UI: 0.996–1.331 billion) cases of migraine worldwide and 2.012 billion (95% UI: 1.777–2.271 billion) cases of TTH. The age-standardized prevalence rate of migraine was 14,246.55 per 100,000 population (95% UI: 12,194.12–16,378.70), while that of TTH was 24,764.77 per 100,000 population (95% UI: 21,863.62–27,954.74). Compared to 1990, the prevalence of migraine increased by 1.56% (95% UI: 1.08–1.87), whereas the prevalence of TTH decreased by 0.56% (95% UI: 0.30–0.44). In 2021, the YLD due to migraine were 532.69 (95% UI: 80.57–1,167.71), while for TTH, the YLD were 55.69 (95% UI: 16.13–185.07) (Supplementary Tables 1, 2).

The global number of migraine cases increased from 732,564,462.68 (95% UI: 624,559,243.92–847,058,436.26) in 1990 to 1,158,432,823.82 (95% UI: 995,861,966.38–1,331,312,506.13) in 2021, with an age-standardized prevalence rate percentage change of 3.12% (95% UI: 1.77–4.15) (Supplementary Table 1; Supplementary Figure 2). Similarly, the global number of TTH cases rose from 1,286,366,671.75 (95% UI: 1,122,503,420.79–1,467,160,187.52) in 1990 to 2,011,612,877.49 (95% UI: 1,776,544,390.83–2,270,860,638.85) in 2021, with an age-standardized prevalence rate percentage change of 0.96% (95% UI: 0.22–1.70) (Supplementary Table 2; Supplementary Figure 4).

Between 1990 and 2021, the YLD burden of TTH showed the most significant increase, rising from 2,848,687.62 (95% UI: 820,890.75–9,563,745.41) in 1990 to 4,596,785.25 (95% UI: 1,347,300.84–15,012,932.75) in 2021, marking a 61.4% increase (Supplementary Table 2). However, the age-standardized YLD rate percentage change was −1.43% (95% UI: −5.21 to 2.27) (Supplementary Figure 4). Similarly, the YLD burden of migraine showed a substantial increase, rising from 27,412,196.29 (95% UI: 4,076,605.01–60,325,805.84) in 1990 to 43,378,889.81 (95% UI: 6,732,642.21–95,079,454.09) in 2021, with an increase of 58.3% (Supplementary Table 1). The age-standardized YLD rate percentage change was 2.02% (95% UI: −3.37 to 4.15) (Supplementary Figure 2).

### Regional level

**Figure 1:**
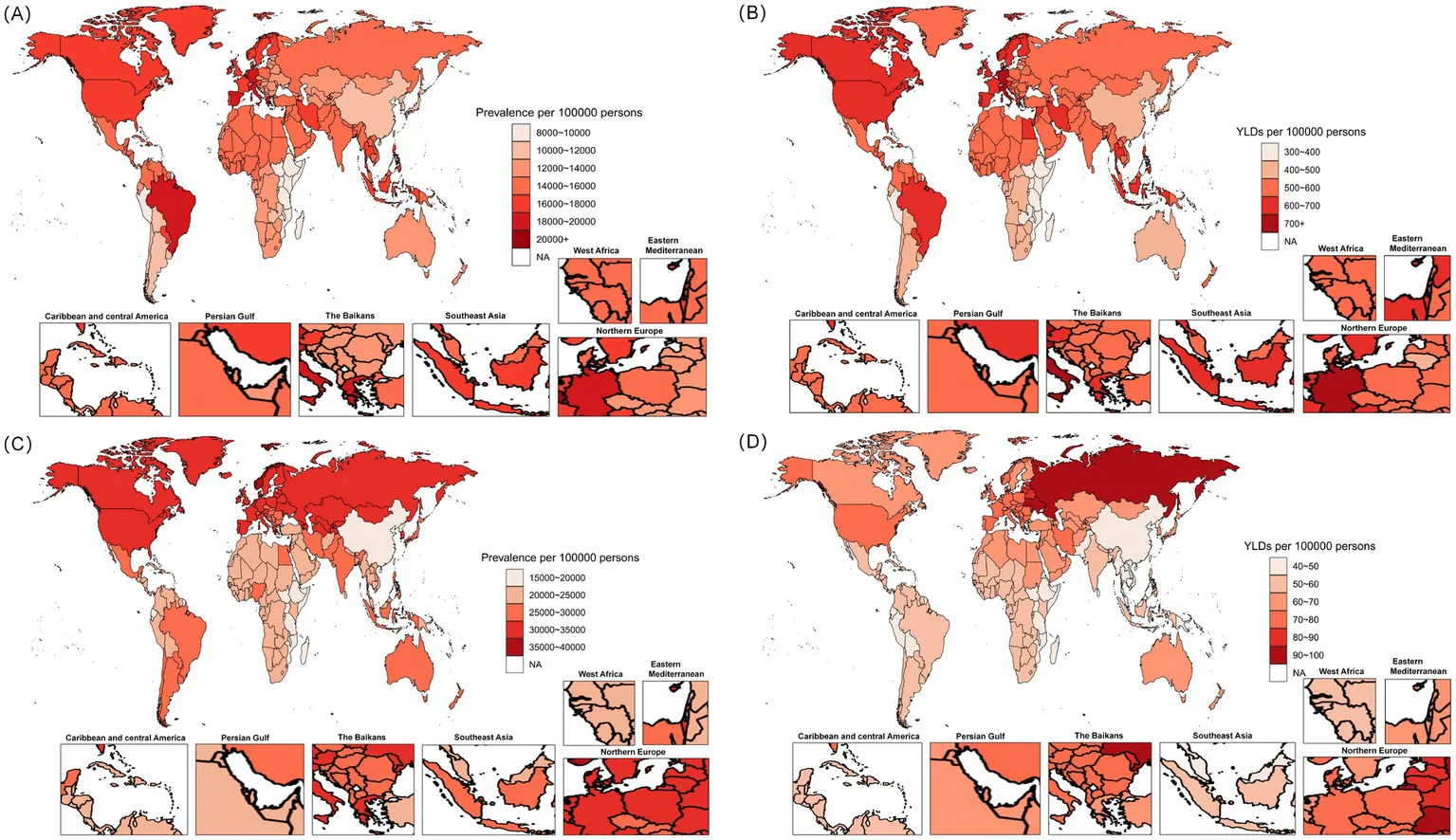
Global migraine and tension-type headache burden: prevalence and YLD rates by country in 2021. **(A)** Age-standardised point prevalence of migraine per 100,000 persons in 2021, by country. **(B)** Age-standardised years lived with disability (YLDs) rate of migraine per 100,000 persons in 2021, by country. **(C)** Age-standardised point prevalence of tension-type headache per 100,000 persons in 2021, by country. **(D)** Age-standardised years lived with disability (YLDs) rate of tension-type headache per 100,000 persons in 2021, by country.

In 2021, the age-standardized prevalence rate of migraines was highest in Latin America and the Caribbean [18,595.34 (95% UI: 16,084.24-21,444.51)] and Western Europe [18,170.70 (95% UI: 15,724.92-21,091.55)], and lowest in Eastern Sub-Saharan Africa [9,084.26 (95% UI: 7,732.97-10,507.26)] (Figure 1; Supplementary Table 1). The age-standardized prevalence rate of TTH (per 100,000 people) was highest in high-income North America [34,180.26 (95% UI: 30,603.85-38,251.42)], Western Europe [32,770.54 (95% UI: 28,999.01-37,028.00)], and Eastern Europe [31,243.12 (27,766.56-34,948.02)], and lowest in East Asia [18,489.51 (16,320.68-20,929.50)] and Eastern Sub-Saharan Africa [18,801.27 (95% UI: 16,305.57-21,690.09)] (Figure 1; Supplementary Table 2).

The age-standardized YLD rate for migraines was highest in Latin America and the Caribbean [679.39 (95% UI: 73.91–1542.87)] and Western Europe [676.64 (95% UI: 96.68–1471.91)], and lowest in Eastern Sub-Saharan Africa [347.19 (95% UI: 77.86–757.55)] (Figure 1; Supplementary Table 1). The highest percentage change in age-standardized YLD rates was observed in East Asia [12.90 (95% UI: 5.35–17.39)], while the lowest was in high-income North America [−14.52 (95% UI: −18.78 to −10.36)] (Supplementary Table 1; Supplementary Figure 1).

For TTH, the age-standardized YLD rate was highest in Eastern Europe [96.83 (95% UI: 33.12–286.15)] and lowest in East Asia [43.40 (95% UI: 13.06–141.04)] and Oceania [46.21 (95% UI: 13.06–163.83)] (Supplementary Figure 1; Supplementary Table 2). The highest percentage change in age-standardized YLD rates was observed in East Asia [11.93 (95% UI: 7.86–16.23)], while the lowest was in high-income North America [−13.38 (95% UI: −18.18 to −8.80)] (Supplementary Table 2; Supplementary Figure 3).

From 1990 to 2021, the Andean region of Latin America experienced the largest change in migraine prevalence, increasing from 9,979.03 (95% UI: 8,656.54-11,428.59) in 1990 to 10,614.71 (95% UI: 8,993.46-12,309.15) in 2021, a rise of 6.37%, the highest globally. Following closely was Southern Latin America, where prevalence grew from 11,393.96 (95% UI: 9,682.23-13,161.22) in 1990 to 11,706.98 (95% UI: 9,960.37-13,667.23) in 2021, marking an increase of 2.75%. Central Sub-Saharan Africa saw the smallest change, with a slight decrease from 12,430.14 (95% UI: 10,518.04-14,549.86) in 1990 to 12,396.15 (95% UI: 10,491.24-14,511.21) in 2021, a reduction of 0.27%. The highest percentage change in age-standardized prevalence rates was observed in East Asia at 11.68 (95% UI: 7.71–15.46), while the lowest was in high-income North America at −2.02 (95% UI: −5.61 to 1.54) (Supplementary Table 1; Supplementary Figure 1).

Regarding TTH prevalence trends, East Asia experienced the most significant change, with rates climbing from 17,188.29 (95% UI: 15,101.02-19,389.89) in 1990 to 18,489.51 (95% UI: 16,320.68-20,929.50) in 2021, an increase of 7.57%. Southern Latin America had the smallest change, with a slight rise from 26,063.87 (95% UI: 22,667.27-30,010.09) in 1990 to 26,264.27 (95% UI: 22,771.94-30,205.10) in 2021, an uptick of 0.77%. The highest percentage change in age-standardized prevalence rates was in Central Sub-Saharan Africa at 10.58 (95% UI: 1.70–20.82), while the lowest was in high-income North America at −13.38 (95% UI: −18.18 to −8.80) (Supplementary Table 2; Supplementary Figure 3).

Additionally, we analyzed the prevalence and YLD rates for all ages globally, across five SDI quintiles, and in 21 regions using the same methodology (Supplementary Figures 5–8).

### National level

Between 1990 and 2021, the age-standardized prevalence rate of migraines saw the largest increases in Singapore [0.4162 (95% UI: 0.3557–0.4767)] and Peru [0.3296 (95% UI: 0.321–0.3381)], while Thailand experienced the largest decrease [−0.2928 (95% UI: −0.3106 to −0.2751)]. The smallest changes were observed in Cote d’Ivoire [−0.0002 (95% UI: −0.0007–0.0003)] and Rwanda [0.0004 (95% UI: −0.0064–0.0073)]. For age-standardized YLD rates of migraines, Singapore [0.3453 (95% UI: 0.2872–0.4035)] and Peru [0.2931 (95% UI: 0.2703–0.316)] had the largest increases, while Thailand [−0.2719 (95% UI: −0.3047 to −0.239)] saw the largest decrease. The smallest changes were in the Philippines [−0.0001 (95% UI: −0.0022–0.0021)] and Poland [−0.0001 (95% UI: −0.0041–0.0039)] (Supplementary Table 1).

For TTH, the age-standardized prevalence rate increased the most in Singapore [0.2644 (95% UI: 0.2486–0.2801)] and China [0.2516 (95% UI: 0.1822–0.321)], while Ethiopia experienced the largest decrease [−0.2982 (95% UI: −0.3067 to −0.2896)]. Malawi showed the smallest change [0 (95% UI: −0.0002–0.0002)]. The age-standardized YLD rate for t TTH increased the most in China [0.1515 (95% UI: 0.1388–0.1642)] and the middle SDI group [0.1507 (95% UI: 0.1207–0.1807)], while Ethiopia [−0.2764 (95% UI: −0.2919 to −0.261)] and Pakistan [−0.2528 (95% UI: −0.2671 to −0.2384)] saw the largest decreases. Bermuda had the smallest change [0.0001 (95% UI: −0.012–0.0122)] (Supplementary Table 2).

**Figure 2:**
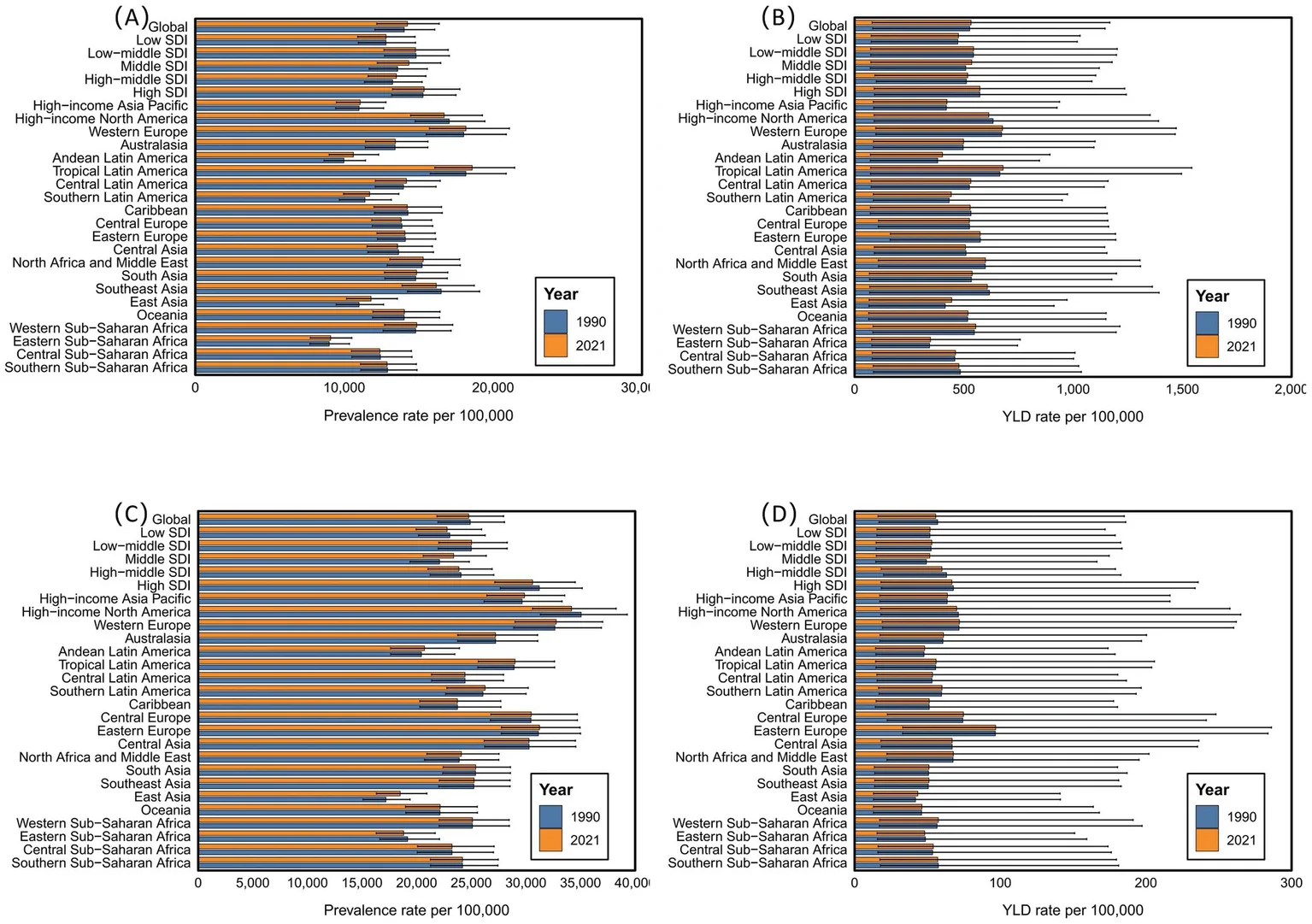
Temporal trend of tension-type headache and migraine burden in regions. **(A)** Prevalence rate per 100,000 persons of migraine in 1990 and 2021. **(B)** YLD rate per 100,000 persons of migraine in 1990 and 2021. **(C)** Prevalence rate per 100,000 persons of tension-type headache in 1990 and 2021. **(D)** YLD rate per 100,000 persons of tension-type headache in 1990 and 2021. YLD, years lived with disability.

In 2021, the highest age-standardized prevalence rates of migraines were observed in Belgium [21,751.47 (95% UI: 18,730.05–25,705.50)], Italy [19,244.27 (95% UI: 16,729.30–22,008.96)], and Germany [19,203.72 (95% UI: 16,546.88–22,548.90)], while the lowest rates were in China [11,777.51 (95% UI: 10,137.56–13,538.56)], the Republic of Maldives [14,991.69 (95% UI: 12,756.93–17,610.83)], and the Philippines [16,273.79 (95% UI: 14,024.28–18,738.94)]. The highest age-standardized YLD rates for migraines were in Belgium [800.36 (95% UI: 91.99–1,772.02)], Italy [717.20 (95% UI: 89.24–1,568.08)], and Germany [707.40 (95% UI: 101.69–1,567.80)], while the lowest rates were in Ethiopia [313.55 (95% UI: 63.99–683.83)], Djibouti [345.08 (95% UI: 79.29–752.63)], and Burundi [347.39 (95% UI: 81.37–756.47)] (Figure 2; Supplementary Table 1).

For TTH, the highest age-standardized prevalence rates in 2021 were in Norway [35,492.44 (95% UI: 31,718.03–39,481.91)], the Netherlands [34,984.91 (95% UI: 30,123.30–39,942.65)], and the United States [34,320.58 (95% UI: 30,762.93–38,348.91)], while the lowest rates were in Ethiopia [15,855.33 (95% UI: 13,637.60–18,206.87)], Taiwan, China [17,557.81 (95% UI: 14,915.25–20,515.45)], and North Korea [17,560.91 (95% UI: 14,930.47–20,516.62)]. The highest age-standardized YLD rates for TTH were in Russia [99.72 (95% UI: 34.79–288.43)], Ukraine [91.72 (95% UI: 29.95–277.45)], and Belarus [88.01 (95% UI: 27.48–269.39)], while the lowest rates were in Ethiopia [40.27 (95% UI: 13.00–128.45)], North Korea [40.51 (95% UI: 11.75–136.01)], and Taiwan, China [41.13 (12.49–134.34)] (Figure 2; Supplementary Table 2).

### Temporal trend

In 1990–2000, the global age-standardized prevalence rate of migraine (per 100,000 population) showed a continuous decline, followed by a positive correlation with time thereafter. For TTH, the global age-standardized prevalence rate declined from 1990 to 2000, then sharply increased from 2000 to 2005, followed by a gradual decline from 2005 to 2015. However, this trend reversed after 2015, reaching its peak in 2020 (Supplementary 2 Figures 1, 2).

**Figure 3:**
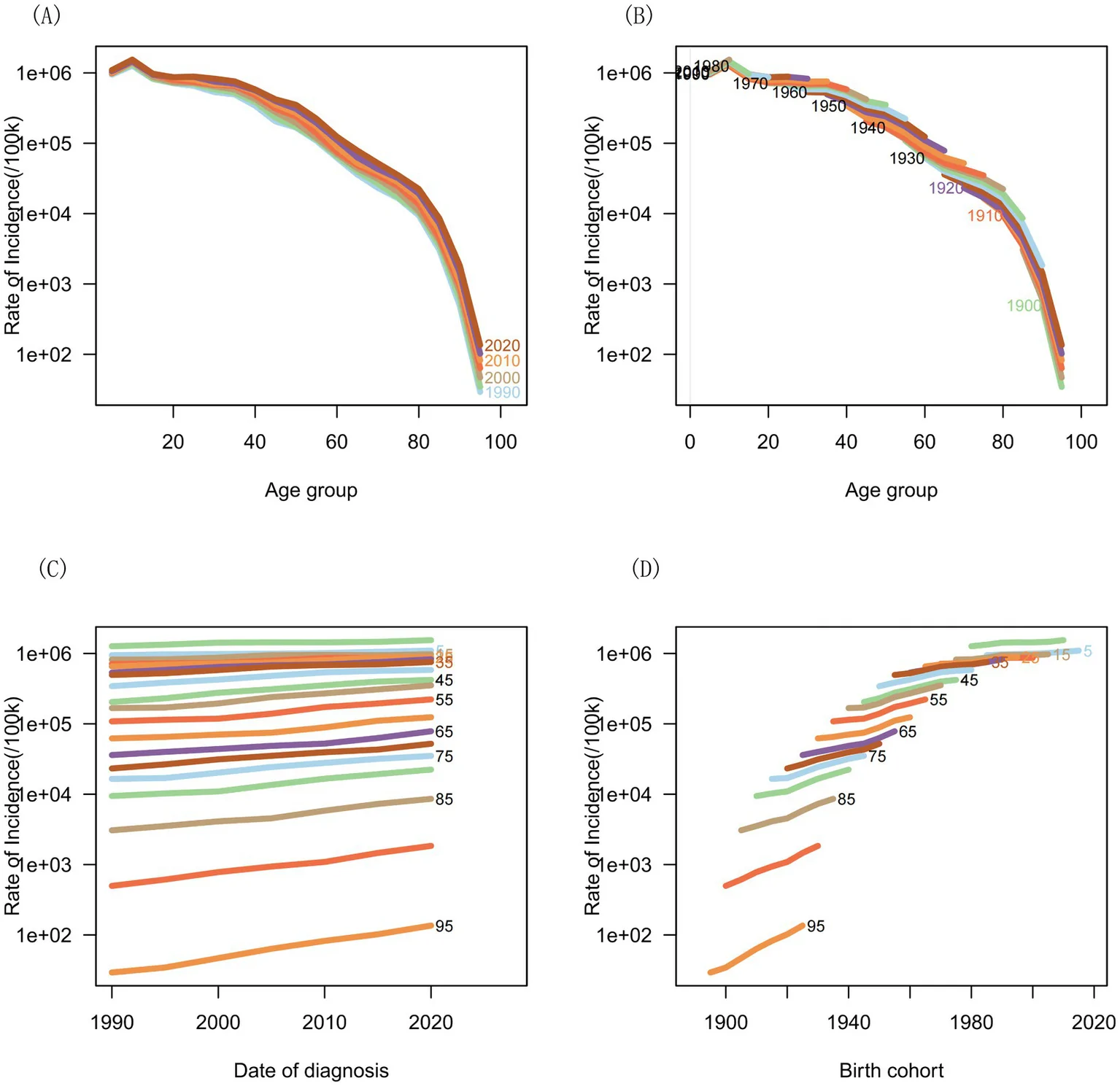
Age, period, and cohort effects on migraine incidence from 1990 to 2020. **(A)** Age group vs. number of incidence (1990–2020): This graph depicts the incidence number (/100 k) across age groups (0–100 years) from 1990 to 2020. The number rises significantly with age, peaking in middle age (40–60 years) and remaining high in older groups (60+), highlighting a strong age-related trend. **(B)** Age group vs. number of incidence (1900–2020): Similar to the 1990–2020 trend, this graph shows incidence numbers increasing with age, with older groups having higher numbers. Fluctuations over time suggest influences from medical advancements, diagnostic changes, or environmental factors. **(C)** Date of diagnosis vs. number of incidence (1990–2020): This graph tracks incidence numbers by diagnosis date (1990–2020). Rates remain relatively stable, with minor fluctuations likely tied to medical advancements or disease prevalence, indicating a weak period effect. **(D)** Birth cohort vs. number of incidence (1900–2020): Incidence numbers are higher in earlier cohorts (1900–1940) and decline in later ones (1980–2020), suggesting improvements in medical conditions, prevention, and lifestyle over time.

**Figure 4:**
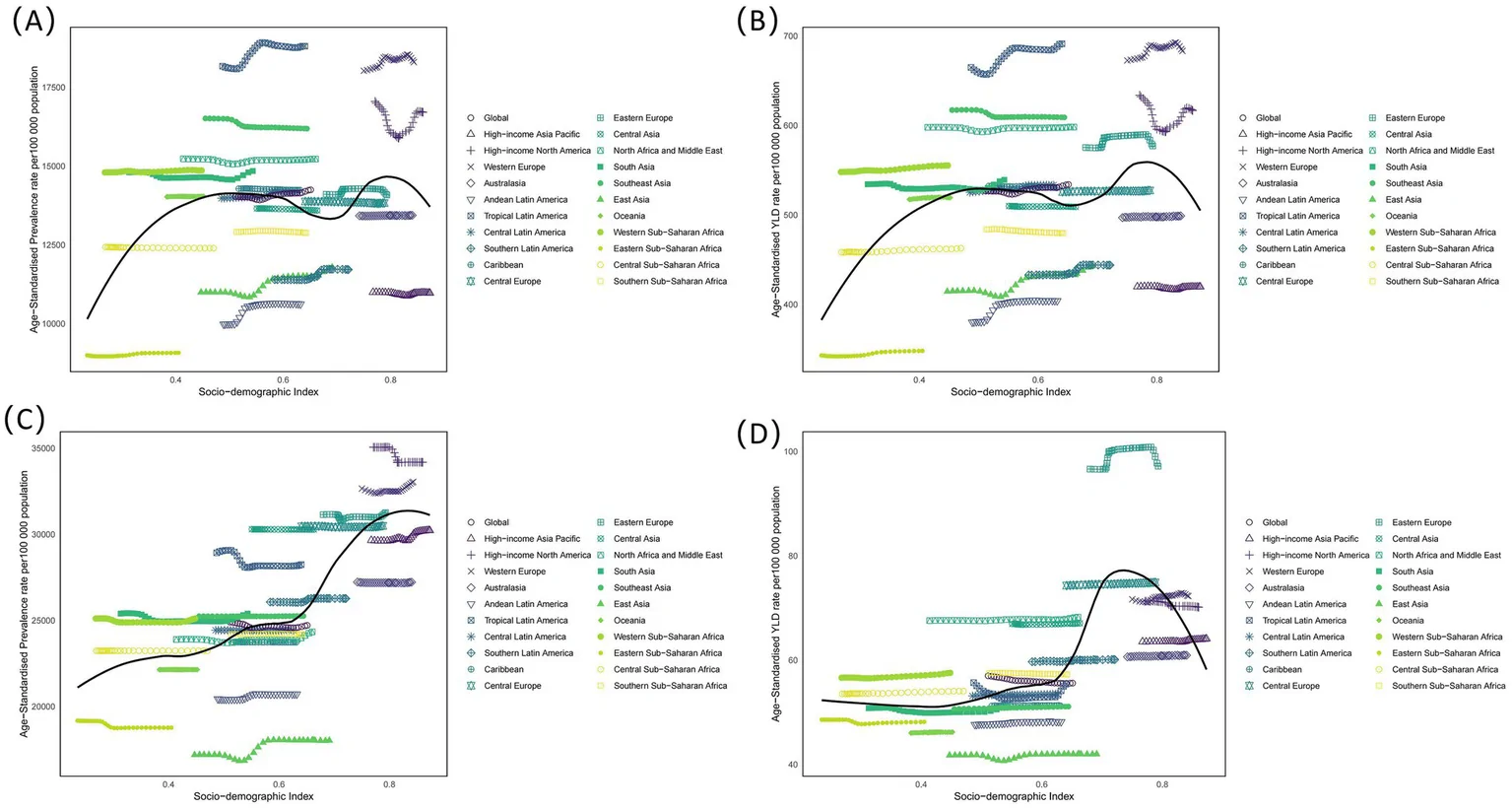
SDI-driven burden of migraine and tension-type headache: prevalence and YLD rate. **(A)** Age−standardised prevalence rate per 100,000 population of migraine. **(B)** Age−standardised YLD rate per 100,000 population of migraine. **(C)** Age−standardised prevalence rate per 100,000 population of tension-type headache. **(D)** Age−standardised YLD rate per 100,000 population of tension-type headache.

The global age-standardized YLD rate of migraine (per 100,000 population) declined from 1990 to 2000, then sharply increased from 2000 to 2005, followed by a slow rise and reaching its peak in 2019. Subsequently, the trend reversed (Supplementary 2 Figures 3, 4).

### Age and sex patterns

In 2021, the prevalence rate of migraines peaked for women in the 40–44 age group [27.48% (95% UI: 22.28–34.32%)], while for men, it peaked in the 30–34 [16.11% (95% UI: 12.63–20.42%)] and 35–39 [16.11% (95% UI: 12.94–19.98%)] age groups (Supplementary Figure 9). The YLD rate for both men [7.80% (95% UI: 0.77–16.72%)] and women [9.34% (95% UI: 0.87–19.82%)] peaked in the 15–19 age group (Supplementary Figure 10). For TTH, the prevalence rate peaked for both men [34.71% (95% UI: 24.14–45.30%)] and women [35.52% (95% UI: 24.76–47.11%)] in the 30–34 age group (Supplementary Figure 11). The YLD rate for men peaked in the 20–24 age group [0.75% (95% UI: 0.17–2.94%)], while for women, it peaked in the 15–19 age group [0.69% (95% UI: 0.16–2.65%)] (Supplementary Figure 12).

To identify the key drivers of burden changes over the past three decades, decomposition analysis was performed to quantify the contributions of population growth, aging, and epidemiological changes. By decomposing the incidence, prevalence, and YLD of migraine and TTH, this study systematically evaluates the impact of aging, population growth, and epidemiological changes on the burden of these two headache disorders from 1990 to 2021. For migraine, the incidence gap between males and females showed a widening trend, with a more pronounced increase in females. Moreover, epidemiological changes (environmental stress, changes in diagnostic criteria) further exacerbated the disease burden. Notably, the effect of aging on reducing incidence showed gender differences, with a more pronounced mitigating effect in females than in males (Supplementary Figure 40). Similar trends were confirmed in the prevalence and YLD decomposition results (Supplementary Figures 41, 42). For TTH, although the gender gap in incidence also widened, the driving mechanisms differed. Aging and epidemiological changes jointly contributed to a reduction in incidence and prevalence, with the decline in incidence more pronounced in females (Supplementary Figure 43) and the decline in prevalence mainly driven by males (Supplementary Figure 44). In terms of YLD, aging increased the burden of TTH, but epidemiological changes (lifestyle interventions) partially offset this effect, with greater benefits observed in females (Supplementary Figure 45).

To explore long-term temporal effects and underlying age-period-cohort patterns, APC analysis was conducted. APC model analysis revealed that migraine incidence exhibited a significant longitudinal trend with age, showing low rates in younger populations, peaking in middle-aged individuals (40–60 years old), and declining in older age groups, suggesting that middle-aged individuals are a high-risk group (Supplementary Figure 13). In cross-sectional analysis, the incidence rate in older groups declined significantly, while the relative risk in middle-aged groups fluctuated (RR: 1.05–1.10), indicating the combined effects of period and cohort influences (Supplementary Figure 13).

From 1990 to 2021, incidence significantly increased across all age groups, showing an exponential rise with age, potentially linked to advancements in diagnostic technology or an actual increase in risk. Cohort effect analysis indicated that individuals born between 1900 and 1940 had a significantly higher incidence rate compared to later birth cohorts, which may reflect differences in unmeasured early-life exposures or broader contextual factors across birth cohorts (Figure 3). Migraine incidence demonstrated a clear age effect (higher risk in older adults compared to younger populations), a stable period effect (minimal trend fluctuations from 1990 to 2020), and a significant cohort effect (earlier birth cohorts had higher incidence rates than recent cohorts), confirming that historical exposure differences across birth cohorts have long-term impacts on disease burden (Figure 3). The analysis of incidence percentages and rates further supports these conclusions (Supplementary Figures 14–17).

The longitudinal age curve for TTH shows a continuous increase in incidence from the age of 20, peaking between 60 and 80 years before slightly declining, suggesting that incidence rates tend to stabilize in the elderly population (Supplementary Figure 18). In the cross-sectional analysis, the relative risk (RR ≈ 1.0) for the different age groups was highly consistent with the longitudinal trend, with a small decrease in the RR value to 0.95 for those aged 80 years and over, further confirming the dominance of the age effect (Supplementary Figure 18) while cohort or period effects are relatively limited. The rate ratio for the 1940 birth cohort is significantly higher than that of the 1980 cohort, indicating that earlier cohorts were which may be associated with differences in early-life conditions or other unmeasured contextual influences across cohorts (Supplementary Figure 19). Between 1990 and 2021, the incidence increased exponentially with age, peaking in the older age group of 80–100 years. This fluctuation may be associated with improvements in medical conditions or changes in diagnostic criteria. Cohort analysis (Supplementary Figure 19) revealed that the incidence rate for the 1900 birth cohort was approximately 10,000–11,000 per 100,000. This rate gradually declined to around 9,000–10,000 per 100,000 in the 1940 cohort and further decreased to approximately 8,000–9,000 per 100,000 in the 1980 cohort (Supplementary Figure 19). This decline reflects the protective effects of modern medical interventions and preventive measures, such as public health policies and improved nutrition, on more recent birth cohorts. The conclusions drawn from percentage-based and rate-based incidence analyses are consistent with these findings (Supplementary Figures 20–23). These findings should be interpreted as descriptive temporal patterns rather than causal effects.

### Association with the socio-demographic index (SDI)

To evaluate health inequalities and socioeconomic gradients, we analyzed the associations between burden and SDI. At the regional level, the disease burden of migraine shows a complex trend in relation to the level of SDI: as SDI increases from a low level (around 0.5), the burden gradually rises; it declines briefly when SDI reaches 0.7, then increases again until around 0.8, after which it declines once more. Overall, there is a positive correlation. From 1990 to 2021, the age-standardized YLD rate for migraine in High-income North America, Tropical Latin America, Western Sub-Saharan Africa, Western Europe, Eastern Europe, North Africa and the Middle East, and Southeast Asia was higher than the SDI-predicted values. In contrast, High-income Asia Pacific, Andean Latin America, Southern Latin America, Eastern Sub-Saharan Africa, Southern Sub-Saharan Africa, Oceania, and East Asia had lower-than-expected values. Except for South Asia, where prevalence was higher than predicted, and Eastern Europe, where it was lower, the age-standardized prevalence and YLD rates of migraine were generally consistent with SDI predictions (Figure 4).

At the national level, from 1990 to 2021, the age-standardized YLD rate of migraine increased with SDI, indicating a higher burden in more socio-economically developed countries. Belgium, Germany, Thailand, Brazil, Russia, and Myanmar had YLD rates significantly higher than the predicted values, while South Africa, China, Switzerland, South Korea, and Japan had lower-than-expected rates. The Central African Republic and Congo had rates approximately in line with expectations. Countries such as Belgium, Brazil, Spain, Portugal, and Italy showed age-standardized prevalence rates significantly above predictions, whereas Japan, South Korea, Chile, and Argentina were significantly below expectations.

At the regional level, the burden of tension-type headache increased steadily with SDI, showing a sharp rise after an SDI of 0.6 and declining after 0.7, indicating a clear positive correlation with socio-economic development. From 1990 to 2021, regions such as Western Sub-Saharan Africa, Central Sub-Saharan Africa, North Africa and the Middle East, Central Asia, and Eastern Europe had age-standardized prevalence rates higher than SDI predictions, whereas Andean Latin America, Eastern Sub-Saharan Africa, Australasia, Oceania, East Asia, South Asia, and Southeast Asia were below expectations.

Unlike migraine, the relationship between SDI and the age-standardized prevalence and YLD rates of tension-type headache showed more variation. High-income North America, Tropical Latin America, Western Sub-Saharan Africa, and South Asia had age-standardized YLD rates higher than SDI-predicted values, while Andean Latin America, Eastern Sub-Saharan Africa, Oceania, and East Asia were below predictions (Figure 4).

At the national level, countries such as Norway, the Netherlands, the United States, and Italy had age-standardized prevalence rates significantly higher than expected, whereas China, Peru, Ecuador, Saudi Arabia, and Guam were below predictions. Russia, Ukraine, Belarus, and Estonia showed YLD rates significantly above expectations, while China, North Korea, Australia, Singapore, and Ethiopia were significantly below.

In addition, the DALY burden of both migraine and tension-type headache showed a decreasing trend when SDI exceeded 0.78, but overall remained positively correlated with SDI (Supplementary Figures 24, 25).

In the analysis of health inequality related to migraine, significant health disparities were identified among countries with different levels of development: both the absolute disparity (difference in the actual number of cases) and the relative disparity (difference in prevalence rates) were significantly associated with the SDI, with low-income countries bearing a heavier burden of migraine. Specifically, the Slope Index of Inequality (SII) worsened from −0.74 in 1990 to −1.91 in 2021 (Supplementary Figure 34), and the CI slightly decreased from 0.1 to 0.08 (Supplementary Figure 35). These results indicate that although there was a slight improvement in the distribution of health, high-income populations continued to hold an advantage. Notably, while relative disparities narrowed, absolute disparities widened over time (Supplementary Figure 36).

A similar pattern was observed in tension-type headache: the SII declined from −0.16 to −0.78 (Supplementary Figure 37), and the CI increased from −0.01 to 0.03 (Supplementary Figure 38), suggesting that the disease burden is shifting from low-income to high-income populations, and overall health inequality continues to intensify (Supplementary Figure 39).

**Figure 5:**
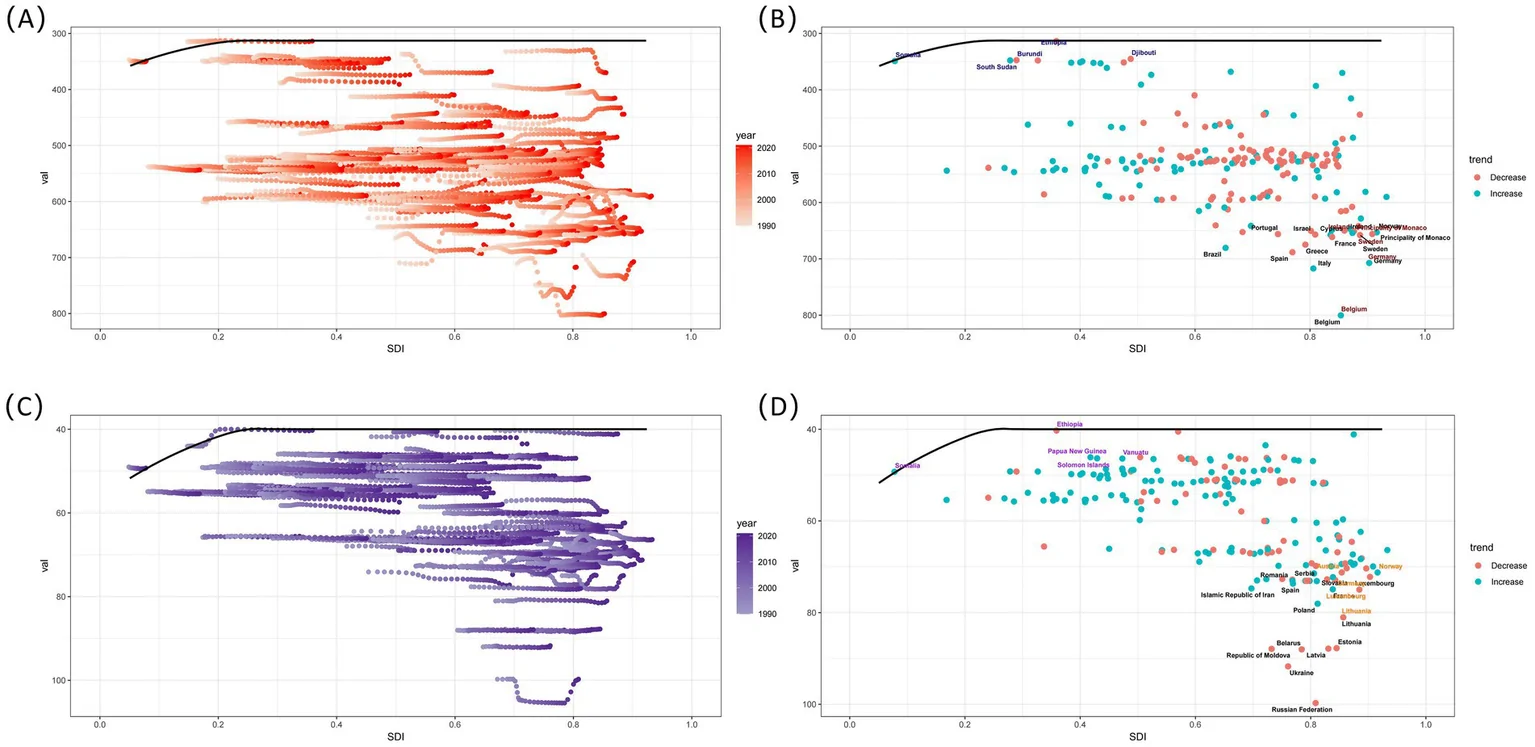
SDI-ASR frontier analysis: migraine and tension-type headache burden (1990–2021). Frontier analysis, represented by the solid black lines, explores the relationship between SDI and age-standardized rate (ASR) for YLDs in the context of migraine **(A,B)** and tension-type headache **(C,D)**. The color gradient in graphs A, C illustrates the progression of years, ranging from light shades representing 1990 to the darkest shades denoting 2021. In graphs **(B,D)** each dot signifies a specific country or territory for the year 2021, with the top 15 countries displaying the most significant deviation from the frontier labeled in black. Countries with low SDI (>0.455) and minimal deviation from the frontier are highlighted in blue or purple, while those with high SDI (>0.805) and notable deviation for their developmental level are emphasized in red or orange. The direction of change from 1990 to 2021 in ASR is indicated by the color of the dots: decrease dots represents a decrease, while increase dots signifies an increase.

Meanwhile, frontier analysis based on SDI and age-standardized YLD rates during the same period revealed spatiotemporal heterogeneity in the burden of migraine and tension-type headache. For migraine, as the SDI value increased from 0.0 to 1.0, the age-standardized YLD rate showed a general downward trend, indicating a gradual reduction in YLD globally (Figure 5). The 2021 frontier analysis showed that in 15 countries, including Belgium and Italy, the actual YLD rates significantly deviated from the theoretical optimal frontier, being much higher than expected for their respective SDI levels. In contrast, low-SDI countries such as Somalia and Ethiopia were closer to the frontier, suggesting relatively better disease burden control given their level of socioeconomic development.

For TTH. Although YLD rates generally declined with increasing SDI, some countries exhibited substantial deviations. Countries like Russia and Ukraine showed notable divergence, while others such as the Somalia and Ethiopia were closer to the theoretical optimal boundary (Figure 5). These findings further reveal the complex and nonlinear relationship between socioeconomic development and the burden of headache disorders.

### Underlying causes

The present study is a secondary analysis of the Global Burden of Disease 2021 database and did not directly measure individual-level risk factors for migraine or tension-type headache. For contextual reference, we summarize evidence from recent high-quality systematic reviews on lifestyle-related triggers and modifiers, as recommended by Quartetti et al. (10).

Consistent with contemporary evidence, multiple modifiable lifestyle factors are associated with migraine frequency and severity, including dietary patterns, physical activity, sleep quality, and psychosocial stress (16–18). Quartetti et al. confirmed that healthy dietary habits, regular physical exercise, adequate sleep, and stress management may reduce migraine attack frequency, whereas skipped meals, dehydration, irregular sleep, and acute or chronic stress may act as potential triggers (10). These factors are widely recognized as important targets for nonpharmacological prevention and management but were not incorporated into the epidemiological modeling of the present study.

## Discussion

Our findings are consistent with previous global burden of disease studies on migraine and tension-type headache. Stovner et al. reported the global prevalence and disability burden from 1990 to 2016 (8); Li et al. focused on young people aged 15–39 years up to 2019 (11); and Zhang et al. described trends in the BRICS countries (12). All confirmed that headache burden was higher in females, peaked in middle age, and increased with population growth and aging. However, the present study offers three unique contributions beyond previous work. First, we updated burden estimates to 2021, enabling the capture of epidemiological trends during the COVID-19 pandemic. Second, we integrated age-period-cohort analysis, health inequality assessment, frontier analysis, and decomposition analysis within a unified framework to provide a comprehensive interpretation. Third, we quantified the drivers of burden changes and performed multi-model future projections, which have not been fully addressed in earlier GBD based reports.

This study systematically evaluated the global burden and trends of migraine and TTH across sex, age, region, and country from 1990 to 2021. Compared to the 2016 analysis, this research improved both modeling complexity and accuracy. A multi-model ensemble was adopted, integrating BAPC models, APC models, ARIMA, Holt-Winters, Joinpoint regression, bootstrap decomposition, and concentration index analysis, forming a multidimensional analytical framework. Using cross-validation and complementary modeling, the study analyzed the total burden, age-standardized rates, demographic differences, socioeconomic gradients, and gender distribution, identifying patterns in the temporal and spatial burden of migraine and TTH. It clarified the roles of population aging, growth, and epidemiological transitions.

Based on GBD 2021—the most comprehensive global dataset—cases of headache rose by 57.16% (95% UI: 56.34–57.66) since 1990, while age-standardized prevalence rose only 0.25% (95% UI: 0.35–0.45). The sharp increase in case numbers, with minimal changes in standardized rates, may reflect rising life expectancy among headache patients (14, 19). These results align broadly with the 2019 migraine study, though this analysis showed slightly higher case increases and smaller prevalence changes.

The trend of increasing case numbers with stable rates suggests the strong impact of demographic shifts and aging on chronic diseases, and also reflects limited effectiveness of public health interventions. Future strategies should aim not only at reducing incidence, but also improving quality of life and functional outcomes to reduce healthcare system stress.

The effect of aging on migraine incidence varied by sex, with a stronger protective effect in women. Decomposition analysis also found that both aging and epidemiologic changes contributed to reduced TTH incidence and prevalence, especially among women.

In terms of sex differences, both prevalence and YLD rates were consistently higher in females. This supports GBD 2016 findings that women are at higher risk. Young and middle-aged adults (15–49 years) bore the heaviest standardized burden, while children under 5 and the elderly had the lowest. These results suggest more resources should target younger populations. Many young individuals are unaware of headache triggers and appropriate management (14, 20).

Physiologically, young women are vulnerable to hormone-related headaches due to estrogen fluctuations, such as menstrual-related migraine (21). Perinatal hormonal shifts may also worsen migraine attacks.

Psychosocially, work, family, and financial pressures in young and middle-aged groups lead to chronic stress, raising TTH risk. Sedentary lifestyle, screen overuse, and poor routines increase headache risk via muscle tension or circadian disruption. This group also shows higher diagnosis rates and health-seeking behavior, partially explaining the prevalence distribution. Current migraine treatment includes drugs (NSAIDs, TCAs) and non-drug therapy (CBT) (15–18). Young women, balancing social and family roles, are more vulnerable to headaches triggered by fatigue and anxiety (19). Lower stress and pain tolerance may increase migraine susceptibility. Moreover, gender-biased drug ads may worsen stigma and reduce care-seeking among women (22).

For forecasting, Bayesian models assessed and predicted age-standardized incidence and YLD rates from 2022 to 2030. From 1990 to 2021, these rates remained largely stable among women, with projections showing little change through 2030.

Unlike the 2019 migraine-only study, this study also used ARIMA and Holt-Winters models to forecast YLD counts. Total burden continued to rise, especially in women, mainly due to population growth and an aging working-age population. Stable standardized YLDs suggest underlying causes remain unresolved. The absence of patient-level data limits the ability to assess treatment impact.

**Figure 6:**
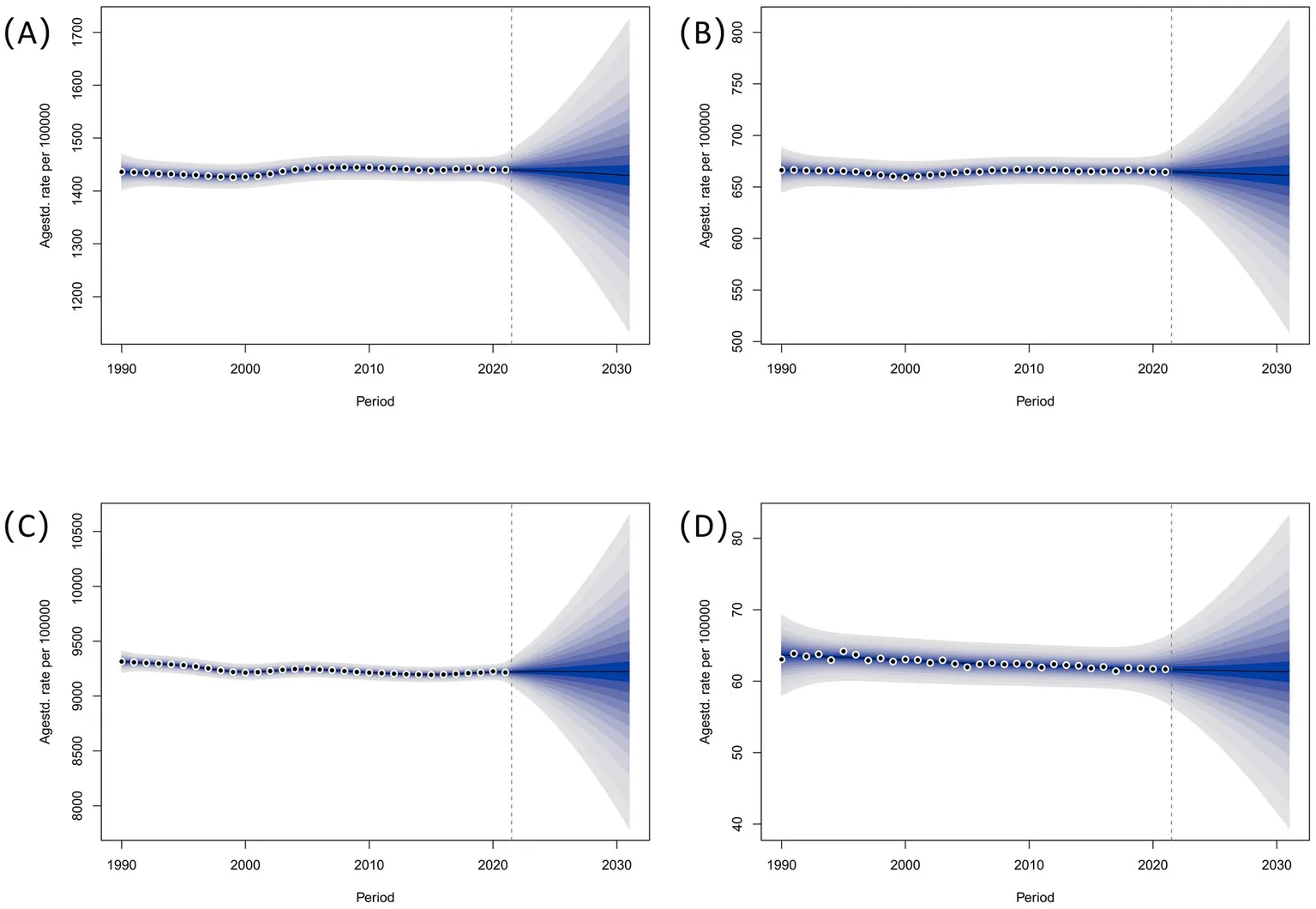
Projected incidence and YLD burden of migraine and tension-type headache: next decade trends. **(A)** Projected numbers and ASR of incidence for migraine from 1990 to 2030 based on the BAPC model. **(B)** Projected numbers and ASR of YLD for migraine from 1990 to 2030 based on the BAPC model. **(C)** Projected numbers and ASR of incidence for tension-type headache from 1990 to 2030 based on the BAPC model. **(D)** Projected numbers and ASR of YLD for tension-type headache from 1990 to 2030 based on the BAPC model.

Regarding the specific prediction results, from 1990 to 2021, the age-standardized incidence rate of tension-type headache among women worldwide remained largely stable (from 1,435.60 to 1,439.72 per 100,000 population, with a change rate of −0.01, 95% UI: −0.02–0.00); the incidence of migraine also showed only a slight increase, reaching 1,438.90 (95% UI: 1,261.41–1,620.05). Prediction results indicate that by 2030, the incidence rates of both types of headache will continue to exhibit minimal changes. The trend in years lived with YLDs is consistent with the incidence rate (Figure 6). However, according to projections from the ARIMA and Holt-Winters models, over the next decade, the absolute number of cases and YLDs among women of all age groups is expected to continue rising (Supplementary Figures 26–33). This phenomenon of “stable age-standardized incidence but increasing absolute numbers” may be attributed to the different dimensions of the two indicators: age-standardized rates eliminate the influence of population structure changes, whereas absolute values directly reflect the combined effects of population growth and aging.

At the regional level, in 2021, migraine had the highest prevalence in tropical Latin America [18,595.34 (95% UI: 16,084.24–21,444.51)], while TTH had the heaviest burden in high-income North America [34,180.26 (95% UI: 30,603.85–38,251.42)]. Both conditions showed relatively high levels in Western Europe, whereas the lowest burden was observed in Eastern sub-Saharan Africa [18,801.27 (95% UI: 16,305.57–21,690.09)].

This pattern may result from high-income countries facing long-term mental stress and intensive cognitive workload. Chronic stress may activate headache pathways through inflammation-related substances such as IL-6 (16). Data from 2019 indicated that Western Europe and North America had some of the highest burdens of illness due to mental health disorders worldwide (22), with high rates of anxiety and depression. There may be a two-way relationship between psychological issues and headaches.

In contrast, traditional diets in sub-Saharan Africa (based on unprocessed grains and vegetables) may have a protective effect by reducing the release of substances that affect blood vessels. However, due to limited medical access, many mild headaches go undiagnosed or unreported, leading to possible underestimation. Additionally, widespread conflict and a lack of mental health services mean that many headache cases remain undetected in this region (21). A previous GBD 2016 analysis suggested that the headache burden in low- and middle-income regions may be underestimated due to “missing data.” This study further confirms such structural underestimation using a concentration index model, particularly in Eastern Europe and parts of Central Asia. These findings support the broader view that nonfatal conditions tend to be underestimated in developing countries within GBD data.

At the national level, some high-income countries (Singapore, Italy) have shown a significant rise in headache prevalence, likely linked to urban stress and high diagnosis rates. In Belgium, Norway, and Italy, the prevalence and YLD rates are much higher than in countries such as Ethiopia and Djibouti. This may reflect more intense mental workloads, inflammatory responses, and hormone imbalances in high-income workforces (23). Urbanization-related factors such as noise, light pollution, and sedentary jobs may also worsen TTH symptoms (24). Moreover, widespread consumption of highly processed foods could trigger migraines by activating prostaglandin pathway (25). Russia presents a notable case, with high headache prevalence and chronicity. Studies show that about 14.5% of Russians aged 18–65 experience daily headaches, and the prevalence of chronic headaches (≥15 headache days/month) reaches 10.5%. Around two-thirds of these cases involve medication overuse, leading to medication-overuse headaches (MOH), a preventable source of health burden (26). Although Russia has developed a multi-tiered headache care system with over 50 tertiary headache centers, the use of preventive medications remains low—fewer than 1% of migraine patients receive preventive treatment, indicating gaps in service use and public awareness (27).

Interestingly, countries like Thailand—a middle-income nation—have shown a declining headache prevalence, suggesting that public health efforts are beginning to pay off. Urbanization, better healthcare access, and improvements in air quality may be key drivers of this trend. These environmental and policy changes appear especially influential in young people.

Analysis based on the APC model reveals the distinct roles of age, period, and birth cohort effects in the evolution of headache-related disease burden. Regarding age effects, the burden of migraine peaks in middle-aged individuals (40–60 years), whereas TTH reaches its maximum in the elderly (60–80 years). This suggests that with physiological aging, factors such as neural degeneration, increased comorbidity of chronic diseases, and disrupted circadian rhythms may significantly contribute to the development and persistence of headache disorders. Moreover, older populations often exhibit cognitive biases or behavioral barriers toward headache recognition and management, further exacerbating the chronicity and disabling impact of the condition. Period effects show relatively minor fluctuations, indicating that short-term changes in social environment, medical infrastructure, and lifestyle have not significantly influenced headache incidence. This may reflect the nature of headache as a chronic, non-lethal condition whose onset and progression are more strongly influenced by long-term biopsychosocial factors. Cohort effects demonstrate that individuals born between 1900 and 1940 bear a significantly higher disease burden than more recent cohorts. This pattern may be related to differences in early-life conditions across generations; however, these interpretations should be considered hypothesis-generating, as such factors were not directly analyzed in the present study, including poor public health conditions, inadequate nutrition, lower education levels, and limited access to healthcare during their formative years. These generational disparities underscore the importance of considering early-life exposures when examining long-term trends in disease burden, and highlight the need for life course–oriented health intervention strategies.

Frontier analysis based on the SDI and age-standardized YLD rates reveals an overall declining trend in both migraine and TTH burdens with increasing socioeconomic development. However, some countries (Russia and Ukraine) exhibit a substantially higher actual burden than predicted by their SDI level. This deviation is likely driven by multiple factors. First, structural deficiencies in healthcare systems—particularly in rural and remote areas—such as uneven distribution of medical resources and inadequate chronic disease management capacity, restrict effective headache control and prevention (28). Second, these countries often have high rates of alcohol consumption, which are strongly associated with increased headache risk and neurological comorbidities. Third, public awareness of headache as a chronic, non-lethal disease is insufficient in some regions, leading to low healthcare-seeking behavior and suboptimal early intervention (29). Psychosocial factors also play a critical role: under high-stress societal contexts, individuals may experience chronic stress, mental disorders, insomnia, poor nutrition, and disrupted circadian rhythms, all of which exacerbate headache frequency and severity. These findings suggest that economic development alone is insufficient to predict disease burden. Structural factors such as healthcare system resilience, accessibility of health services, and sociopolitical stability must also be considered in future burden estimation models.

In the analysis of health inequities, this study constructs a CI and SII model to track the evolution of disease burden in countries with different levels of social development from 1990 to 2021. For migraine, the SII decreased from −0.74 to −1.91, reflecting a significant widening of absolute health disparities. Meanwhile, the CI slightly decreased (from 0.10 to 0.08), indicating that diseases are still more concentrated in lower-income countries, but the improvement has been limited. A similar trend was observed for tension-type headaches, with changes in SII and CI moving in opposite directions, suggesting that the burden of this disease is slowly shifting from low-income to high-income populations, although low-SDI countries still face a heavier burden. We believe that low-SDI countries generally face issues such as insufficient medical resources, a shortage of specialized doctors, and weak basic health systems, which make it difficult for patients to access early diagnosis and standardized treatment, thus exacerbating the disease burden (30). Public awareness of the chronic mechanisms and prevention of headaches is lacking, and there is weak awareness of standardized medication use, with medication-overuse headache (MOH) being more common in low-income regions. Moreover, adverse social and environmental factors such as intense labor, psychological stress, malnutrition, and air pollution are more prominent in these countries, further increasing the risk of disease onset and delaying recovery (8). It is particularly important to note that insufficient disease monitoring and reporting capabilities may result in data underestimation or bias, limiting the accurate allocation of resources. Despite the increasing global interventions on chronic diseases, headache disorders are often not prioritized in low- and middle-income countries, and public health investment remains insufficient.

### Strengths and limitations of the study

The GBDdatabase, as an authoritative health data platform, provides important evidence for assessing the epidemiological characteristics of various system diseases and formulating prevention and control strategies. This study conducted a systematic analysis of the disease burden of migraines and tension-type headaches based on GBD data, but there are several limitations that need to be addressed.

At the data level, the quality of the research results depends on the reliability of the raw data from various countries (31). It is worth noting that low-income countries often lack standardized disease monitoring systems and representative epidemiological surveys, leading to data gaps that need to be filled using statistical models like DisMod-MR, which may introduce estimation bias. In addition, the unique diagnostic characteristics of headache disorders (relying on subjective symptom reports) and lower medical consultation rates may result in an underestimation of the disease burden.

Methodologically, although the APC/AAPC model can effectively analyze long-term trends, its linear assumption may fail to capture nonlinear impacts brought about by sudden public health events (the COVID-19 pandemic). The computational efficiency advantages of Bayesian prediction models may be weakened by improper prior distribution settings, particularly during significant changes in medical technology or social structures. In time series analysis, the differencing process of the ARIMA model may lose key information, while the Holt-Winters model’s over-sensitivity to recent data (high *α* value) can also affect the robustness of the prediction.

In terms of health equity assessment, although advanced methods such as Bootstrap decomposition, concentration index, and SDI-YLD frontier analysis were used, the SDI fails to encompass job-related stress and other headache-specific risk factors, which may lead to biased assessments of disease burden in high-SDI countries. Additionally, the GBD framework categorizes headache disorders as non-fatal diseases (YLL = 0), which, while in line with current standards, may underestimate their indirect effects on mortality through comorbidities (depression).

It should be particularly noted that the current disability assessment based on health surveys may not fully reflect the comprehensive impact of headaches on patients’ social functioning and occupational performance. Differences in symptom reporting due to cultural factors, as well as the disease burden among specific populations (adolescents and the elderly) that may be masked by age-standardization methods, need to be considered when interpreting the results.

## Conclusion

This study found that the overall prevalence of both types of headaches has significantly increased, primarily driven by population growth and aging, with a particularly heavy burden among young and middle-aged women, closely related to hormonal fluctuations and social psychological stress. Geographically, higher prevalence rates were observed in high-income regions (North America, Western Europe), which may be related to lifestyle and diagnostic biases, while the lower prevalence in low-income regions (Sub-Saharan Africa) likely reflects inadequate diagnosis, although protective factors such as traditional diets may play a regulatory role. These findings highlight the need for targeted intervention strategies that consider high-risk populations and regional differences.

Methodologically, the study innovatively integrates seven models, including Bayesian hierarchical regression, ARIMA, Holt, and APC, achieving an in-depth, multidimensional, and dynamic analysis of the disease burden. Compared to existing visualization websites that only provide static indicators, this study not only quantifies the temporal trends of disease burden but also significantly enhances the reliability of the results through cross-validation between models. Moreover, the study innovates the APC model by reapplying age standardization to the original data, effectively avoiding potential age structure biases in the GBD database, making the separation of age, period, and cohort effects clearer, and significantly improving the model’s explanatory power and predictive accuracy. The study also innovatively combines the ARIMA model and the Holt model, using mutual verification to ensure the robustness of overall predictions.

In terms of practical value, the study is the first to systematically reveal the spatiotemporal patterns of migraine and tension-type headache prevalence and disability rates across different genders, age groups, regions, and countries, providing scientific evidence for developing targeted prevention and control strategies. The multi-model analysis framework is not only applicable to headache disease research but also provides a methodological paradigm for the disease burden assessment of other chronic diseases.

Based on these findings, future research should focus on: (1) improving data collection systems and diagnostic standards, particularly in regions with scarce medical resources; (2) developing statistical models that better reflect the characteristics of headache disorders; (3) incorporating more social determinants into the analysis framework. These improvements will help enhance the accuracy of disease burden assessments, establish a more effective global headache disease prevention and control system, and provide more reliable evidence-based support for public health decision-making.

## Data Availability

The data analyzed in this study is subject to the following licenses/restrictions: the data that support the findings of this study are available from the Global Burden of Disease (GBD) 2021 study. Restrictions apply to the availability of these data, which were used under license for the current study, and so are not publicly available. Data are however available from the authors upon reasonable request and with permission of the GBD study. Requests to access these datasets should be directed to: http://ghdx.healthdata.org/gbd-results-tool.
