# Supplementary material for: The global burden of migraine and tension-type headache, 1990–2021: a multi-model Bayesian meta-regression with decomposition of age-period-cohort effects, health inequalities, and frontier analysis
Source: Front Neurol. 2026 Aug 18;17:1818932. doi: 10.3389/fneur.2026.1818932 (PMC13529543; doi:10.3389/fneur.2026.1818932)
Supplement: Supplementary file 1 [file Supplementary_file_1.pdf]

Supplementary Tables and Figures for”Global, regional, and national burden of migraine and tension-type headache and its underlying causes from 1990 to 2021”

Contents

Supplementary Table 1. Prevalent Cases and YLDs for Migraine in 2021, Percentage Change in Age-Standardized Rates by Location, and Average Annual Percentage Change..............................5

Supplementary Table 2. Prevalent Cases and YLDs for Tension-Type Headache in 2021, Percentage Change in Age-Standardized Rates by Location, and Average Annual Percentage Change..21

Supplementary Figure 1. Percentage Change in Age-Standardized YLDs and Prevalence Rates for Migraine by Region（1990-2021）..37

Supplementary Figure 2. Percentage Change in Age-Standardized YLDs and Prevalence Rates for Migraine by SDI quintiles level（1990-2021）...38

Supplementary Figure 3. Percentage Change in Age-Standardized YLDs and Prevalence Rates for Tension-Type Headache by Region（1990-2021）...39

Supplementary Figure 4. Percentage Change in Age-Standardized YLDs and Prevalence Rates for Tension-Type Headache by SDI quintiles level（1990-2021）...40

Supplementary Figure 5. Percentage Change in All-Age YLDs and Prevalence Rates for Migraine by Region（1990-2021）..41

Supplementary Figure 6. Percentage Change in All-Age YLDs and Prevalence Rates for Migraine by SDI quintiles level （1990-2021）..42

Supplementary Figure 7. Percentage Change in All-Age YLDs and Prevalence Rates for Tension-Type Headache by Region（1990-2021）..43

Supplementary Figure 8. Percentage Change in All-Age YLDs and Prevalence Rates for Tension-Type Headache by SDI quintiles level （1990-2021）..44

Supplementary Figure 9. Prevalence of Migraine by Gender and Age Group................................45

Supplementary Figure 10. YLDs of Migraine by Gender and Age Group.......................................46

Supplementary Figure 11. Prevalence of Tension-Type Headache by Gender and Age Group.......47

Supplementary Figure 12. YLDs of Tension-Type Headache by Gender and Age Group.................48

Supplementary Figure 13. Age-Period-Cohort (APC) Model Analysis of Migraine Incidence Number: Epidemiological Characteristics of Age Effects, Period Trends, and Cohort Differences................49

Supplementary Figure 14. Age-Period-Cohort (APC) Model Analysis of Migraine Incidence Rate: Epidemiological Characteristics of Age Effects, Period Trends, and Cohort Differences.................50

Supplementary Figure 15. Age, Period, and Cohort Effects on Migraine Incidence Rate: A Comprehensive Analysis from 1900 to 2020...51

Supplementary Figure 16. Age-Period-Cohort (APC) Model Analysis of Migraine Incidence Percent: Epidemiological Characteristics of Age Effects, Period Trends, and Cohort Differences................52

Supplementary Figure 17. Age, Period, and Cohort Effects on Migraine Incidence Percent: A Comprehensive Analysis from 1900 to 2020...53

Supplementary Figure 18. Age-Period-Cohort (APC) Model Analysis of Tension-Type Headache Incidence Number: Epidemiological Characteristics of Age Effects, Period Trends, and Cohort Differences...54

Supplementary Figure 19. Age, Period, and Cohort Effects on Tension-Type Headache Incidence Number: A Comprehensive Analysis from 1900 to 2020..55

Supplementary Figure 20. Age-Period-Cohort (APC) Model Analysis of Tension-Type Headache Incidence Rate: Epidemiological Characteristics of Age Effects, Period Trends, and Cohort Differences...56

Supplementary Figure 21. Age, Period, and Cohort Effects on Tension-Type Headache Incidence Rate: A Comprehensive Analysis from 1900 to 2020..57

Supplementary Figure 22. Age-Period-Cohort (APC) Model Analysis of Tension-Type Headache Incidence Percent: Epidemiological Characteristics of Age Effects, Period Trends, and Cohort Differences...58

Supplementary Figure 23. Age, Period, and Cohort Effects on Tension-Type Headache Incidence Percent: A Comprehensive Analysis from 1900 to 2020...59

Supplementary Figure 24. Age-standardised disability-adjusted-life-years (DALY) rates for Migraine by 21 Global Burden of Disease regions by Socio-demographic Index (SDI), 1990–2021....60

Supplementary Figure 25. Age-standardised disability-adjusted-life-years (DALY) rates for Tension-Type Headache by 21 Global Burden of Disease regions by Socio-demographic Index (SDI), 1990–2021....61

Supplementary Figure 26. Forecasting YLDs for Migraine in the Global Female Population Across All Age Groups Using the Holt-Winters Algorithm....62

Supplementary Figure 27. Forecasting YLDs for Migraine in the Global Female Population Across All Age Groups Using the ARIMA Algorithm....63

Supplementary Figure 28. Forecasting Incidence for Migraine in the Global Female Population Across All Age Groups Using the Holt-Winters Algorithm....64

Supplementary Figure 29. Forecasting Incidence for Migraine in the Global Female Population Across All Age Groups Using the ARIMA Algorithm....65

Supplementary Figure 30. Forecasting YLDs for Tension-Type Headache in the Global Female Population Across All Age Groups Using the Holt-Winters Algorithm....66

Supplementary Figure 31. Forecasting YLDs for Tension-Type Headache in the Global Female Population Across All Age Groups Using the ARIMA Algorithm....67

Supplementary Figure 32. Forecasting Incidence for Tension-Type Headache in the Global Female Population Across All Age Groups Using the Holt-Winters Algorithm....68

Supplementary Figure 33. Forecasting Incidence for Tension-Type Headache in the Global Female Population Across All Age Groups Using the ARIMA Algorithm....69

Supplementary Figure 34. The Crude Incidence of Migraine by Socio-demographic Index (SDI) in 1990 and 2021....70

Supplementary Figure 35. The Cumulative Fraction of Migraine Incidence by Cumulative Fraction of Population Ranked by SDI in 1990 and 2021....71

Supplementary Figure 36. The Slope Index of Inequality (SII) for Migraine Incidence from 1990 to 2021....72

Supplementary Figure 37. The Crude Incidence of Tension-Type Headache by Socio-demographic Index (SDI) in 1990 and 2021....73

Supplementary Figure 38. The Cumulative Fraction of Tension-Type Headache Incidence by Cumulative Fraction of Population Ranked by SDI in 1990 and 2021..74

Supplementary Figure 39. The Slope Index of Inequality (SII) for Tension-Type Headache Incidence from 1990 to 2021...75

Supplementary Figure 40. Changes in Migraine Incidence by Sex from 1990 to 2021 Based on Population-Level Determinants of Aging, Population Growth, and Epidemiological Changes.......76

Supplementary Figure 41. Changes in Migraine Prevalence by Sex from 1990 to 2021 Based on Population-Level Determinants of Aging, Population Growth, and Epidemiological Changes.......77

Supplementary Figure 42. Changes in Migraine YLDs by Sex from 1990 to 2021 Based on Population-Level Determinants of Aging, Population Growth, and Epidemiological Changes.......78

Supplementary Figure 43. Changes in Tension-Type Headache Incidence by Sex from 1990 to 2021 Based on Population-Level Determinants of Aging, Population Growth, and Epidemiological Changes..79

Supplementary Figure 44. Changes in Tension-Type Headache Prevalence by Sex from 1990 to 2021 Based on Population-Level Determinants of Aging, Population Growth, and Epidemiological Changes..80

Supplementary Figure 45. Changes in Tension-Type Headache YLDs by Sex from 1990 to 2021 Based on Population-Level Determinants of Aging, Population Growth, and Epidemiological Changes..81

Supplementary Table 1. Prevalent Cases and YLDs for Migraine in 2021, Percentage Change in Age-Standardized Rates by Location, and Average Annual Percentage Change

	Migraine							
	Prevalence				YLD			
	2021 counts	The age-standardized prevalence rate	Percentage change in age-standardised rates, 1990–2021	AAPC	2021 counts	The age-standardized YLD rate	Percentage change in age-standardised rates, 1990–2021	AAPC
**Global**	**1158432823.82（995861966.38-1331312506.13）**	**14246.55**	**3.12%(1.77%—4.15%)**	**0.0506（0.0444—0.0568）**	**43378889.81（6732642.21-95079454.09）**	**532.70**	**2.0%(-3.4%—4.1%)**	**0.0357（0.0321—0.0393）**
High SDI	175424403.7（153279198.8-201895736.97）	15365.14	1.59%(-0.09%—3.46%)	0.0155（0.0074—0.0236）	6613112.28（1212857.08-14047722.29）	573.62	-4.7%(-7.6%—-2.3%)	-0.0012（-0.0119—0.0096）
High-middle SDI	191802656.26（167163077.16-220197710.99）	13502.73	4.62%(3.08%—5.97%)	0.0622（0.0493—0.075）	7409676.32（1474226.66-15665836.88）	517.57	4.4%(-5.3%—7.7%)	0.0372（0.0277—0.0468）
Middle SDI	371941648.12（319831620.31-426895953.87）	14344.23	8.09%(6.32%—9.66%)	0.1732（0.1601—0.1863）	13921317.27（2023382.92-30544094.04）	535.85	6.0%(2.5%—8.3%)	0.1687（0.1633—0.1741）
Low-middle SDI	288676927.2（246439632.67-332108194.77）	14786.99	1.69%(-0.04%—3.32%)	-0.0078（-0.0128—-0.0027）	10617256.96（1368932.85-23440099.63）	544.25	4.6%(1.2%—7.9%)	0.0009（-0.002—0.0039）
Low SDI	129654870.34（109563874.32-152432455.61）	12808.97	1.29%(0.02%—2.35%)	0.0028（-0.0142—0.0198）	4782245.51（672592.65-10758550.44）	475.21	8.1%(4.4%—11.2%)	0.0212（0.0042—0.0382）
**High-income North America**	**63986396.11（55643984.98-73406886.01）**	**16709.11**	**-2.02%(-5.61%—1.54%)**	**-0.0683（-0.0814—-0.0553）**	**2366729.89（390228.63-5145721.19）**	**614.00**	**-14.5%(-18.8%—-10.4%)**	**-0.1017（-0.1211—-0.0823）**
Canada	6323300.85（5470147.18-7383158.95）	16333.55	-1.33%(-6.31%—4.44%)	-0.0594（-0.064—-0.0547）	237313.39（39336.52-514041.34）	607.71	-6.7%(-12.7%—-0.6%)	-0.065（-0.0798—-0.0503）
Greenland	9518.76（8158.64-11111.18）	16078.52	3.36%(2.81%—4%)	0.0785（0.0637—0.0932）	353.36（54.82-763.68）	593.26	2.7%(-3.4%—8.2%)	0.0717（0.0366—0.1067）
United States of America	57652573.11（50084784.22-66079074.29）	16750.24	-2.09%(-5.92%—1.94%)	-0.0682（-0.08—-0.0565）	2129026.03（350575.18-4628603.34）	614.68	-15.3%(-19.6%—-10.6%)	-0.1049（-0.1285—-0.0813）
**Australasia**	**4324576.91（3732120.1-4964955.8）**	**13433.71**	**-0.21%(-1.06%—0.67%)**	**0.0029（0.0023—0.0036）**	**162013.02（31164.38-351253.66）**	**498.64**	**2.2%(-1.5%—5.9%)**	**0.012（0.0062—0.0179）**
Australia	3571618.48（3065934.39-41	13338.06	-0.42%(-1.43%—0.66%)	0.0056（0.0053—0.	133810.77（25590.92-2910	494.98	1.5%(-3.1%—6.1%)	0.0096（0.0006—

	20713.64）			006）	10.17）			0.0186）
New Zealand	752958.43（655401.42-861493.81）	13912.65	0.75%(-0.25%—1.67%)	-0.0117（-0.0211—-0.0024）	28202.26（5414.69-61064.37）	517.01	5.5%(1.7%—9.6%)	0.0086（-0.0065—0.0236）
**High-income Asia Pacific**	**21172926.84（18371952-24296845.67）**	**11072.69**	**3.67%(0.23%—7.19%)**	**0.0278（0.0164—0.0392）**	**827640.33（204646.32-1773190.03）**	**422.48**	**2.6%(-1.1%—6.0%)**	**0.0254（0.0121—0.0386）**
Brunei Darussalam	52654.26（44637.28-62275.8）	10292.50	2.50%(1.86%—3.25%)	0.0142（0.0101—0.0183）	2015.08（406.11-4508.36）	393.16	0.4%(-4.0%—5.0%)	0.0136（0.0061—0.0212）
Japan	13831413.44（12019193.67-15876262.53）	10818.40	5.14%(0.77%—9.63%)	0.0797（0.0738—0.0855）	545229.14（141779.57-1160180.18）	415.34	3.5%(-1.0%—7.5%)	0.0737（0.0576—0.0899）
Singapore	603543.18（519624.83-703071.65）	9471.09	-1.20%(-6.55%—4.64%)	0.4162（0.3557—0.4767）	24170.44（6063.33-52095.98）	370.02	1.2%(-5.1%—8.9%)	0.3453（0.2872—0.4035）
Republic of Korea	6685315.96（5762126.87-7766627.64）	11812.12	16.91%(7.32%—26.64%)	-0.1394（-0.2179—-0.0608）	256225.67（55453.62-555436.6）	444.10	16.2%(3.1%—26.5%)	-0.1101（-0.235—0.015）
**Western Europe**	**80359608.3（70005541.78-93109621.14）**	**18170.70**	**1.93%(0.35%—3.64%)**	**0.0235（-0.0018—0.0487）**	**3026513.28（527630.22-6473769.21）**	**676.64**	**-0.6%(-3.0%—1.8%)**	**0.0184（-0.0121—0.0489）**
Andorra	16182.8（13861.95-18948.69）	17391.72	1.73%(1.35%—2.18%)	0.0373（0.0086—0.0661）	610.96（105.11-1323.79）	646.76	-3.2%(-7.8%—1.4%)	0.0354（0.0139—0.057）
Austria	1522682.97（1315123.42-1761091.77）	16466.12	0.08%(-0.39%—0.61%)	-0.0164（-0.03—-0.0028）	57720.25（10769.14-124519.76）	615.73	-0.1%(-5.1%—4.2%)	-0.0186（-0.0386—0.0015）
Belgium	2544608.37（2192801.83-2985664.82）	21751.47	8.12%(1.42%—16.47%)	0.2444（0.2379—0.2508）	94014.86（12767.28-205439.93）	800.36	2.9%(-7.3%—11.4%)	0.2248（0.1981—0.2515）
Cyprus	258627.6（222725.23-302839.91）	17622.86	1.43%(1.11%—1.85%)	0.0175（0.0165—0.0184）	9731.92（1569.49-21129.2）	657.50	-0.1%(-4.3%—4.9%)	0.0176（0.0101—0.0251）
Denmark	906327.27（783204.77-1046930.3）	15422.39	3.47%(-1.59%—8.98%)	0.091（0.0767—0.1053）	34566.93（6198.3-72993.07）	581.19	4.1%(-2.9%—11.0%)	0.0793（0.0682—0.0903）
Finland	959037.81（826906.73-1120219.04）	17477.69	-0.32%(-0.88%—0.23%)	-0.0169（-0.0174—-0.0164）	36012.12（6289.88-77781.08）	649.91	-0.8%(-5.1%—3.3%)	-0.0221（-0.0289—-0.0153）
France	11508601.68（9881348.61-13376867.86）	17478.90	0.18%(-4.16%—4.74%)	-0.0238（-0.0295—-0.0182）	441400.79（84487.74-934151.12）	661.31	-1.7%(-8.1%—4.5%)	-0.0287（-0.0465—-0.0109）
Germany	16539322.38	19203.72	3.77%(-1.02%—10.17%)	0.0712	615737.75	707.40	-0.6%(-7.8%—5.6%)	0.0447

	（14277979.53-19290791.57）			（0.0563—0.086）	（104953.06-1316251.1）			（0.0233—0.0662）
Greece	1868500.63 （1599645.43-2180107.87）	18254.82	1.75%(-2.34%—6.74%)	-0.0071 （-0.0085—-0.0057）	69985.94 （11835.2-151360.14）	674.80	-4.4%(-10.5%—1.5%)	-0.0213 （-0.0351—-0.0076）
Iceland	62574.14 （53834.22-72796.51）	17439.61	0.66%(0.09%—1.23%)	-0.0146 （-0.0209—-0.0083）	2352.48 （381.6-5109.59）	651.01	0.2%(-3.9%—4.4%)	-0.0169 （-0.0313—-0.0025）
Ireland	890679.36 （765137.9-1040070.43）	17632.30	1.27%(0.76%—1.78%)	0.0111 （0.0092—0.0129）	33360.28 （5525.86-72824.76）	654.12	-3.0%(-7.9%—2.0%)	0.0029 （-0.0094—0.0152）
Israel	1646071.46 （1408912.11-1929109.68）	17572.11	0.59%(0.13%—1.16%)	-0.0195 （-0.0201—-0.0189）	61690.19 （9154.26-135395.15）	656.91	-1.5%(-5.9%—2.6%)	-0.0273 （-0.0409—-0.0137）
Italy	11636929.73 （10174340.39-13422449.68）	19244.27	1.96%(-1.25%—4.75%)	0.0211 （-0.1043—0.1468）	438568.65 （68925.67-944341.92）	717.20	3.3%(-0.7%—7.0%)	0.0273 （-0.0994—0.1543）
Luxembourg	116291.68 （99994.13-136017.1）	16915.46	0.66%(0.28%—1.17%)	-0.0128 （-0.0249—-0.0008）	4472.92 （872.26-9474.61）	641.87	1.9%(-1.9%—6.3%)	-0.0085 （-0.0251—0.0082）
Malta	77439.46 （66742.88-90119.67）	17448.60	0%(-0.43%—0.42%)	-0.0293 （-0.0299—-0.0286）	2921.2 （517.52-6307.73）	650.36	-3.7%(-8.1%—0.2%)	-0.0384 （-0.0463—-0.0306）
Netherlands	2944900.50 （2542076.55—3405005.83）	17015.11	6.38%(0.96%—12.66%)	0.1855 （0.1194—0.2517）	109685.32 （16353.25—242318.90）	628.54	0.9%(-7.0%—8.2%)	0.1715 （0.1349—0.2081）
Norway	980506.95 （852993.92-1124376.32）	17700.09	8.19%(4.55%—12%)	0.2404 （0.2092—0.2715）	36469.37 （5565.68-79611.21）	653.12	10.7%(5.2%—15.2%)	0.2458 （0.2137—0.2779）
Portugal	1903084.5 （1643638.13-2225481.89）	17692.88	1.34%(0.88%—1.85%)	-0.004 （-0.005—-0.0031）	71559.35 （12909.69-153420.92）	656.01	0.7%(-4.3%—5.4%)	-0.0065 （-0.0206—0.0076）
Spain	8537877.56 （7350314.09-10025982）	18266.01	0.91%(0.14%—1.71%)	-0.0037 （-0.0065—-0.001）	327242.61 （62354.28-698496.34）	688.24	-2.0%(-7.3%—3.0%)	-0.0062 （-0.0341—0.0218）
Sweden	1846704.34 （1613904.8-2112037.97）	17826.53	0.54%(-2.77%—4.15%)	-0.0024 （-0.0042—-0.0005）	68595.04 （10307.23-147741.68）	657.82	-2.8%(-7.7%—2.7%)	-0.0205 （-0.0376—-0.0035）
Switzerland	1444817.2 （1250524.02-1670106.75）	15818.27	2.11%(-2.29%—6.12%)	0.0287 （0.0226—0.0347）	54637.32 （10398.48-117888.09）	590.17	3.9%(-2.2%—10.5%)	0.0301 （0.0119—0.0483）
United Kingdom	12064770.46 （10517925.66-13963773.48）	17467.16	2.18%(-0.99%—5.71%)	0.0264 （0.0237—0.0292）	452047.07 （77045.83-968291.88）	648.34	-2.4%(-6.3%—1.5%)	0.0156 （0.013—0.0181）

Monaco	6438.22 (5553.35-7523.67)	17639.08	-0.07%（-0.16%—0.01%）	-0.002（-0.005—0.0011）	243.53 (45.70-521.42)	655.88	-0.62%（-3.58%—2.41%）	-0.0192（-0.028—-0.0104）
San Marino	5877.84 (5077.87-6881.18)	17738.70	0.73%（0.52%—0.96%）	0.0236（0.0228—0.0245）	221.72 (39.07-478.47)	660.00	0.31%（-2.93%—3.35%）	0.0100（0.0033—0.0167）
**Southern Latin America**	**8402383.48（7195355.61-9789775.46）**	**11706.98**	**4.59%(0.82%—9.49%)**	**0.0872（0.0781—0.0963）**	**318959.43（64871.36-694704.17）**	**442.43**	**-0.2%(-5.2%—4.8%)**	**0.0692（0.0558—0.0827）**
Argentina	5586220.21（4760123.67-6541041.91）	11636.39	5.41%(0.65%—11.48%)	0.1095（0.1048—0.1141）	212471.19（42665.6-467064.62）	441.01	0.6%(-5.7%—7.5%)	0.0942（0.0694—0.119）
Chile	2397437.62（2048235.4-2801326.08）	11862.87	3.42%(-1.02%—8.46%)	0.0557（0.0508—0.0606）	90555.54（19078.42-195348.24）	445.42	-0.3%(-7.2%—6.2%)	0.0392（0.0283—0.0501）
Uruguay	418261.92（357929.96-487885.46）	11758.22	1.31%(0.45%—2.20%)	0.0024（0.0016—0.0031）	15915.09（3412.04-34532.33）	444.07	-5.7%(-10.5%—-1.5%)	-0.0141（-0.0243—-0.0038）
**Eastern Europe**	**32050565.68（27899660.01-36442160.85）**	**14077.09**	**1.37%(1.10%—1.68%)**	**-0.006（-0.0109—-0.0011）**	**1336530.24（424522.73-2695926.7）**	**574.43**	**0.1%(-4.2%—2.8%)**	**-0.0055（-0.0197—0.0087）**
Belarus	1331465.04（1154466.27-1524260.49）	13015.65	0.95%(0.54%—1.40%)	-0.019（-0.0201—-0.018）	55176.12（17071.72-112397.35）	526.93	-2.5%(-7.8%—2.4%)	-0.0235（-0.034—-0.0129）
Estonia	180463.75（156480.8-206209.93）	12900.48	0.52%(0.03%—1%)	-0.0456（-0.0477—-0.0434）	7476.86（2289.15-15185.74）	523.11	0.3%(-4.2%—5.0%)	-0.0367（-0.0475—-0.0258）
Latvia	259186.37（225126.13-296312.02）	12978.48	0.61%(-0.04%—1.27%)	-0.0316（-0.0332—-0.03）	10761.04（3321.95-21824.39）	526.11	1.3%(-4.4%—6.7%)	-0.0253（-0.0365—-0.0141）
Lithuania	358843.34（310510.04-412286.05）	12244.96	1.46%(0.74%—2.25%)	-0.0439（-0.0528—-0.035）	14584.74（4246.62-29905.12）	487.22	-2.6%(-8.9%—2.6%)	-0.0361（-0.0483—-0.0239）
Republic of Moldova	527387.15（457303-607970.5）	12971.64	0.69%(0.18%—1.24%)	-0.0398（-0.0405—-0.0392）	21846.58（6672.59-44583.8）	526.37	1.4%(-3.8%—6.7%)	-0.0362（-0.0459—-0.0265）
Russian Federation	22799067.61（19758993.61-25864413.57）	14379.09	1.40%(1.10%—1.73%)	-0.0091（-0.0152—-0.0029）	955153.59（306140.85-1915841.84）	589.48	0.5%(-3.9%—3.1%)	-0.0058（-0.0211—0.0095）
Ukraine	6594152.42（5746869.78-7566954.18）	13592.26	0.31%(-0.06%—0.68%)	-0.0227（-0.0233—-0.022）	271531.32（83932.12-543384.43）	548.06	-1.8%(-7.2%—2.6%)	-0.0198（-0.0299—-0.0097）
**Central Europe**	**17302363.75（15177932.24-1**	**13822.04**	**1.87%(1.40%—2.33%)**	**-0.0125（-0.0141—-**	**666284.65（159796.29-143**	**525.24**	**2.6%(-1.0%—5.1%)**	**-0.0024（-0.0079—**

	**9780847.61）**			**0.0109）**	**6227.37）**			**0.0031）**
Albania	392413.1（337140.67-452887.21）	13598.05	3.44%(2.80%—4.21%)	0.0217（0.019—0.0245）	15072.29（3582.1-32681.51）	516.38	1.6%(-5.0%—7.5%)	0.0206（0.009—0.0322）
Bosnia and Herzegovina	491055.62（425225.04-566028.59）	13632.57	2.47%(1.94%—3.03%)	-0.0036（-0.0096—0.0024）	18815.34（4515.3-40504.83）	515.27	-1.4%(-7.1%—3.8%)	-0.011（-0.0255—0.0034）
Bulgaria	994945.37（861408.71-1144457.2）	13595.28	0.98%(0.52%—1.46%)	-0.0191（-0.0209—-0.0173）	38217.27（9300.7-82359.53）	514.42	-2.1%(-8.3%—3.2%)	-0.0205（-0.0343—-0.0066）
Croatia	615855.2（536629.95-712210.72）	13584.21	1.64%(-2.74%—5.86%)	-0.0314（-0.0459—-0.0168）	23744.19（5717.8-50875.52）	516.50	0.8%(-5.7%—6.8%)	-0.0196（-0.0407—0.0016）
Czechia	1539807.14（1332072.98-1769906.12）	13581.76	1.02%(0.56%—1.52%)	-0.0263（-0.0267—-0.0258）	59096.6（14003.58-127917.2）	514.44	0.2%(-5.4%—5.4%)	-0.0227（-0.0326—-0.0128）
Hungary	1420158.83（1231619.28-1633084.72）	13659.57	1.66%(1.08%—2.29%)	-0.0174（-0.0181—-0.0167）	54587.84（13037.44-117434.66）	517.97	5.5%(0.2%—11.0%)	-0.0037（-0.0121—0.0047）
North Macedonia	331180.87（284824.85-383702.41）	13534.57	1.72%(1.20%—2.33%)	-0.0248（-0.0282—-0.0213）	12682.44（2942.47-27528.89）	512.39	-3.6%(-10.0%—1.9%)	-0.0298（-0.0418—-0.0179）
Montenegro	90944.24（78570.22-104953.56）	13632.03	1.61%(1.07%—2.18%)	-0.0051（-0.0063—-0.004）	3483.26（786.48-7549.62）	516.27	-6.0%(-11.8%—-0.7%)	-0.0202（-0.0307—-0.0098）
Poland	5973361.25（5222975.69-6772298.13）	14258.61	1.39%(0.91%—1.88%)	-0.0151（-0.0155—-0.0148）	230605.64（56407.91-492545.37）	543.31	3.6%(0.0%—6.2%)	-0.0001（-0.0041—0.0039）
Romania	2766760.79（2398063.67-3185736.85）	13609.33	2.06%(1.52%—2.62%)	-0.0167（-0.0181—-0.0152）	106718.69（25310.75-228723.74）	517.26	4.9%(-0.4%—10.6%)	0.0013（-0.0079—0.0104）
Serbia	1325082.46（1147908.98-1527772.03）	13545.29	2.58%(2%—3.28%)	-0.0259（-0.0311—-0.0207）	50906.11（12097.85-109628.21）	514.68	-0.9%(-6.5%—4.0%)	-0.0279（-0.0403—-0.0156）
Slovakia	810276.29（699862.57-935455.64）	13618.05	1.13%(0.62%—1.62%)	-0.0237（-0.0246—-0.0228）	31139.92（7227.28-67224.49）	516.94	1.9%(-3.4%—7.3%)	-0.0156（-0.029—-0.0022）
Slovenia	298680.72（258727.88-343964.28）	13531.47	0.81%(0.21%—1.41%)	-0.0366（-0.0372—-0.036）	11517.06（2765.6-24685.68）	513.87	2.7%(-2.5%—7.8%)	-0.0288（-0.0387—-0.0189）
**Central Asia**	**13240029.5（11217466.19-15557584.1）**	**13584.23**	**1.11%(0.77%—1.46%)**	**-0.0162（-0.017—-0.0155）**	**494678.9（87857.7-1116618.46）**	**507.35**	**0.2%(-3.7%—3.0%)**	**-0.0168（-0.0217—-0.0119）**
Armenia	437577.01	13628.90	1.46%(1.05%—1.91%)	-0.0063	16484.99	510.08	0.9%(-4.6%—6.4%)	-0.004

	（374797.72-511583.41）			（-0.0081—-0.0045）	（3165.89-36641.4）			（-0.0135—0.0054）
Azerbaijan	1551484.7 （1317970.39-1820179.98）	13532.87	0.64%(0.18%—1.11%)	-0.0379 （-0.0392—-0.0366）	58294.77 （10896.45-130062.44）	506.66	-1.9%(-8.6%—3.4%)	-0.0392 （-0.0472—-0.0313）
Georgia	508334.72 （434385.21-591389.75）	13553.71	-0.29%(-0.84%—0.25%)	-0.037 （-0.0396—-0.0344）	19122.92 （3852.47-42047.92）	505.78	-7.3%(-13.1%—-2.0%)	-0.0552 （-0.0778—-0.0326）
Kazakhstan	2626667.59 （2226458.7-3080362.17）	13656.47	1.96%(1.24%—2.65%)	-0.0039 （-0.0049—-0.0028）	98006.52 （17958.06-220735.74）	508.64	1.2%(-4.6%—6.4%)	-0.0033 （-0.0127—0.0061）
Kyrgyzstan	915872.76 （772858.59-1077304.38）	13622.21	1.25%(0.75%—1.76%)	-0.0055 （-0.0078—-0.0033）	34132.29 （5869.65-77685.37）	509.39	3.5%(-2.7%—9.2%)	-0.0022 （-0.0145—0.0102）
Mongolia	450789.46 （381218.64-530449.89）	13624.59	2.92%(2.43%—3.42%)	0.0169 （0.0164—0.0175）	16773.44 （2852.49-38126.65）	507.53	0.4%(-4.3%—4.8%)	0.0129 （0.0038—0.022）
Tajikistan	1322078.95 （1116232.94-1560178.08）	13521.62	1.05%(0.65%—1.47%)	-0.0125 （-0.0161—-0.0089）	49216.86 （8048.06-112918.3）	506.14	0.2%(-5.7%—5.2%)	-0.0142 （-0.0256—-0.0028）
Turkmenistan	702053.89 （593583.68-826206.01）	13422.84	0.22%(-0.30%—0.72%)	-0.0514 （-0.0524—-0.0504）	26287.21 （4639.92-59522.91）	503.16	1.0%(-4.3%—6.4%)	-0.0383 （-0.0481—-0.0286）
Uzbekistan	4725170.44 （3998863.36-5559333.69）	13577.32	1.32%(0.68%—1.90%)	-0.0086 （-0.0094—-0.0078）	176359.91 （31420.82-397878.22）	507.07	0.8%(-4.9%—5.5%)	-0.0055 （-0.012—0.001）
**Central Latin America**	**37489337.72 （32024848.75-43508285.84）**	**14178.67**	**4.04%(1.42%—6.61%)**	**0.0456 （0.0363—0.0548）**	**1406733.3 （203911.79-3062757.11）**	**531.67**	**2.5%(-1.3%—6.1%)**	**0.0377 （0.0315—0.0438）**
Colombia	7469770.1 （6249063.94-8788757.58）	14382.09	3.87%(-0.82%—9.69%)	0.0359 （0.0314—0.0403）	280615.5 （39185.57-614033.5）	539.64	4.2%(-3.1%—12.3%)	0.0363 （0.0139—0.0587）
Costa Rica	709915.78 （601857.38-832962.73）	14094.56	2.78%(2.16%—3.41%)	0.0326 （0.0319—0.0333）	26628.76 （3830.73-57774.72）	527.91	-4.6%(-9.9%—0.4%)	0.0183 （0.011—0.0255）
El Salvador	935408.05 （795016.22-1100747.23）	14345.72	4.65%(3.95%—5.41%)	0.0466 （0.0451—0.0482）	34977.77 （4784.27-76320.32）	537.03	8.6%(3.5%—14.4%)	0.0604 （0.0511—0.0696）
Guatemala	2270498.3 （1917138.45-2689247.91）	14154.35	3.61%(3%—4.29%)	0.0242 （0.0237—0.0247）	84011.07 （10845.78-186618.12）	524.95	5.3%(-0.3%—11.5%)	0.0301 （0.0191—0.0411）
Honduras	1451167.78 （1224291.1-1720655.88）	14156.32	3.03%(2.35%—3.79%)	0.0305 （0.0297—0.0312）	53960.33 （6643.57-119696.86）	527.94	-3.4%(-9.3%—2.1%)	0.0142 （0.0049—0.0235）

Mexico	19212341.36（16538504.67-22337785.29）	14127.41	4.06%(-0.37%—8.23%)	0.0416（0.035—0.0483）	722310.25（109702.24-1587845.34）	530.62	2.2%(-2.8%—6.8%)	0.0313（0.0276—0.035）
Nicaragua	961781.58（815163.89-1134044.85）	14032.73	2.15%(1.49%—2.87%)	-0.0156（-0.0161—-0.0151）	35919.18（4733.91-78659.11）	524.90	2.4%(-3.7%—8.2%)	-0.0116（-0.0171—-0.006）
Panama	606862.63（514703.72-712398.79）	13876.05	2.34%(1.62%—3.12%)	0.0037（0.0019—0.0055）	22789.33（3207.29-49539.05）	520.94	-2.6%(-7.4%—2.0%)	-0.0051（-0.0114—0.0011）
Venezuela (Bolivarian Republic of)	3871592.13（3281413.33-4478143.14）	14161.13	5.97%(0.89%—12.38%)	0.1204（0.1089—0.132）	145521.11（21341.45-317773.63）	530.58	3.2%(-4.8%—11.2%)	0.1093（0.0904—0.1281）
**Andean Latin America**	**7215330.92（6090862.48-8396291.19）**	**10614.71**	**7.47%(3.15%—13.86%)**	**0.1987（0.1897—0.2078）**	**272813.8（48480.19-611279.56）**	**401.89**	**6.8%(-0.5%—13.6%)**	**0.1799（0.1526—0.2072）**
Bolivia (Plurinational State of)	1310699.94（1104327.05-1522133.39）	10894.77	0.62%(0.16%—1.05%)	-0.0239（-0.0243—-0.0235）	49147.57（8219.85-109553.95）	409.78	-0.4%(-6.6%—5.9%)	-0.0311（-0.0417—-0.0205）
Ecuador	2287822.55（1915710.03-2701523.6）	12383.18	4.20%(-1.31%—11.10%)	0.0816（0.0742—0.0891）	85731.31（13021.52-188128.47）	464.61	1.0%(-7.2%—9.9%)	0.063（0.0442—0.0817）
Peru	3616808.43（3050028.05-4201120.66）	9645.45	11.07%(4.08%—20.29%)	0.3296（0.321—0.3381）	137934.92（27416.12-307040.77）	368.08	12.0%(-0.8%—23.8%)	0.2931（0.2703—0.316）
**Caribbean**	**6949002（5879711.94-8046252.38）**	**14237.12**	**0.81%(0.56%—1.06%)**	**-0.01（-0.0109—-0.009）**	**258591.18（36827.98-563192.48）**	**528.99**	**-4.2%(-6.5%—-1.9%)**	**-0.0199（-0.0264—-0.0134）**
Antigua and Barbuda	13744.86（11657.57-15935.56）	14278.95	0.58%(0.02%—1.14%)	-0.0195（-0.022—-0.017）	514.34（76.62-1123.55）	532.29	-4.4%(-9.6%—0.6%)	-0.0311（-0.045—-0.0171）
Bahamas	60687.03（51342.88-70212.34）	14343.18	0.92%(0.47%—1.41%)	0.0039（0.0029—0.0048）	2264.43（321.76-4954.58）	533.91	-4.7%(-9.5%—0.0%)	-0.0109（-0.0194—-0.0023）
Barbados	44624.11（37934.93-51620.47）	14292.50	0.48%(0.03%—0.92%)	-0.0124（-0.0128—-0.0121）	1676.85（265.61-3646.26）	533.58	-5.2%(-9.7%—-0.6%)	-0.0232（-0.0385—-0.0079）
Belize	64437.8（54006.93-75422.4）	14249.40	2.48%(1.96%—3.09%)	0.0301（0.0294—0.0307）	2392.51（304.09-5248.87）	530.30	-5.5%(-10.8%—-0.6%)	0.0098（0.0015—0.0182）
Bermuda	9175.66（7863.79-10635.54）	14239.41	0.82%(0.40%—1.24%)	-0.0105（-0.012—-0.0089）	347.92（60.57-753.74）	534.40	-2.4%(-7.1%—2.3%)	-0.0131（-0.0219—-0.0042）
Cuba	1650955.14（1411247.35-19	14147.38	1.97%(1.30%—2.59%)	-0.0083（-0.0086—-	62238.21（10331.35-1351	529.47	-0.9%(-5.3%—3.8%)	-0.0049（-0.0101—

	20508.08）			0.008）	50.9）			0.0002）
Dominica	10063.1（8509.11-11619.98）	14091.22	0.04%(-0.54%—0.57%)	-0.0336（-0.0347—-0.0325）	375.57（54.84-822.32）	524.43	-6.5%(-11.9%—-1.9%)	-0.0492（-0.0603—-0.0381）
Dominican Republic	1610280.57（1356666.97-1869166.99）	14169.24	0.83%(0.40%—1.24%)	-0.0351（-0.0364—-0.0337）	59959.75（8091.4-130569.69）	527.84	-4.5%(-9.0%—0.3%)	-0.0443（-0.0531—-0.0354）
Grenada	15340.19（12958.62-17854.93）	14061.13	-0.01%(-0.45%—0.41%)	-0.0494（-0.0508—-0.048）	572.74（82.4-1246.85）	523.74	-4.0%(-9.0%—1.1%)	-0.0511（-0.0608—-0.0414）
Guyana	112237.64（94272.04-130466.94）	14282.58	1.80%(1.31%—2.24%)	0.0035（-0.0009—0.0078）	4122.93（552.36-9053.96）	524.86	-4.0%(-8.6%—1.0%)	-0.01（-0.0207—0.0007）
Haiti	1854945.01（1552408.62-2174454.9）	14339.78	0.71%(0.40%—1.07%)	-0.004（-0.0055—-0.0025）	67950.59（8311.41-147484.85）	527.01	1.3%(-4.0%—6.7%)	-0.001（-0.0189—0.0169）
Jamaica	427856.27（361326.06-497956.06）	14226.31	0.57%(0.09%—1.02%)	-0.0159（-0.0167—-0.0151）	16000.34（2247.44-35029.95）	531.53	-4.3%(-9.0%—0.3%)	-0.0264（-0.0336—-0.0193）
Puerto Rico	482949.78（412712.72-559391.92）	14317.03	0.64%(-0.04%—1.36%)	-0.0113（-0.012—-0.0107）	18151.12（2995.55-39206.55）	535.12	-2.3%(-7.1%—2.0%)	-0.0158（-0.0269—-0.0046）
Saint Lucia	27076.64（22958.42-31432.79）	14161.11	0.10%(-0.38%—0.56%)	-0.043（-0.0436—-0.0423）	1009.74（155.6-2191.4）	525.64	-2.8%(-8.0%—2.3%)	-0.0487（-0.0618—-0.0355）
Saint Vincent and the Grenadines	16769.77（14211-19358.64）	14107.49	1.19%(0.55%—1.81%)	-0.0264（-0.027—-0.0258）	625.3（94.13-1362.5）	524.41	-5.8%(-10.3%—-1.4%)	-0.0412（-0.0502—-0.0322）
Suriname	85371.81（72232.79-98712.61）	14248.98	1.70%(1.25%—2.23%)	0.0133（0.011—0.0157）	3161.38（464.71-6928.06）	526.65	-7.8%(-13.5%—-2.1%)	-0.0139（-0.0232—-0.0045）
Trinidad and Tobago	206096.27（174679.17-238282.77）	14155.63	1.10%(0.35%—1.74%)	-0.0098（-0.0102—-0.0094）	7681.13（1185.87-16798.9）	525.41	-5.5%(-10.7%—-1.0%)	-0.0245（-0.0337—-0.0152）
United States Virgin Islands	12145.15（10427.06-14112.32）	14272.60	0.06%(-0.39%—0.52%)	-0.0241（-0.0265—-0.0217）	455.44（77.56-977.43）	531.68	-6.8%(-11.3%—-2.1%)	-0.0376（-0.0488—-0.0263）
Saint Kitts and Nevis	9083.32 (7692.64-10535.26)	14237.12	-0.77%（-0.93%—-0.62%）	-0.0248（-0.0263—-0.0233）	339.89 (52.40-744.11)	527.92	-0.64%（-3.99%—2.82%）	-0.0140（-0.0191—-0.009）
**Tropical Latin America**	**43632767.96（37739023.24-50335352.35）**	**18595.34**	**3.73%(0.24%—7.32%)**	**0.0852（0.0409—0.1295）**	**1594752.2（187099.65-3590842.76）**	**679.39**	**2.5%(-2.2%—8.1%)**	**0.0599（0.0118—0.108）**
Brazil	42324293.4	18631.33	3.84%(0.29%—7.56%)	0.0896	1546788.87	680.61	2.7%(-2.2%—8.5%)	0.0631

	（36640634.82-48830055.1）			（0.0435—0.1358）	（182557.33-3486212.83）			（0.0137—0.1125）
Paraguay	1308474.56（1094481.31-1547869.57）	17467.73	1.03%(0.62%—1.47%)	-0.0075（-0.008—-0.0069）	47963.33（4684.61-109466.2）	640.61	-4.9%(-9.9%—0.7%)	-0.0227（-0.0299—-0.0155）
**East Asia**	**191704023.87（166846937.79-221713278.72）**	**11798.36**	**11.68%(7.71%—15.46%)**	**0.2281（0.2146—0.2416）**	**7248392.37（1171602.56-15744802.47）**	**444.29**	**12.9%(5.4%—17.4%)**	**0.225（0.2106—0.2393）**
China	184752280.11（160836524.72-213633958.29）	11777.51	12%(7.86%—15.89%)	0.2356（0.2216—0.2497）	6988198.6（1133318.67-15186289.26）	443.65	13.3%(5.4%—18.1%)	0.2325（0.2177—0.2473）
Democratic People's Republic of Korea	3466449.5（2946693.03-4050958.14）	11798.27	0.88%(0.20%—1.65%)	-0.0752（-0.0763—-0.0741）	130252.74（18577.29-286441.69）	441.94	1.4%(-3.0%—6.8%)	-0.0665（-0.0704—-0.0627）
Taiwan	3485294.25（2990346.31-4096089.41）	13042.93	5.93%(-1.20%—12.66%)	0.1443（0.1374—0.1512）	129941.03（18548.69-286886.12）	485.07	1.4%(-6.6%—9.2%)	0.1305（0.1115—0.1496）
**Southeast Asia**	**119860813.04（103266385.91-139236439.49）**	**16180.66**	**0.86%(-0.08%—1.75%)**	**-0.0654（-0.0704—-0.0603）**	**4504042.89（522075.9-10047420.78）**	**607.13**	**3.4%(0.8%—7.2%)**	**-0.0528（-0.0596—-0.0459）**
Cambodia	2753302.73（2328218.69-3245974.24）	15875.26	0.69%(0.13%—1.24%)	-0.0497（-0.0504—-0.049）	102892.25（10642.45-238208.42）	592.98	12.7%(6.7%—21.7%)	-0.0159（-0.023—-0.0087）
Indonesia	48937911.94（41949667-56395685.92）	16269.49	2.57%(2.08%—3.07%)	-0.0168（-0.0173—-0.0164）	1845660.08（219017.34-4117372.94）	612.37	4.5%(1.3%—7.5%)	-0.0073（-0.011—-0.0036）
Lao People's Democratic Republic	1177649.71（992323.07-1391694.88）	15770.99	1.69%(1.21%—2.23%)	-0.0277（-0.0285—-0.0268）	44160.43（4561.15-101957.45）	591.40	9.7%(4.9%—15.5%)	-0.0055（-0.0116—0.0005）
Malaysia	4880619.18（4131860.86-5682692.53）	14260.88	5.02%(0.69%—10.30%)	0.0485（0.0466—0.0503）	184140.38（21534.19-412520.85）	537.85	2.0%(-4.8%—8.8%)	0.0434（0.035—0.0519）
Maldives	89867.04（75947.04-106939.04）	14991.69	-0.76%(-1.83%—0.14%)	-0.1378（-0.1443—-0.1313）	3371.51（362.99-7618.02）	562.08	8.8%(3.4%—17.1%)	-0.1124（-0.1307—-0.0941）
Mauritius	220838.94（187832.26-260053.23）	15779.33	2.04%(1.29%—2.83%)	-0.0026（-0.0031—-0.002）	8215.44（997.43-18211.45）	586.53	-1.9%(-7.3%—2.6%)	-0.011（-0.0252—0.0033）
Myanmar	9253223.58（7825988.17-10929907.46）	15924.77	2.74%(2.30%—3.21%)	0.0126（0.0121—0.0132）	346237.72（37501.13-792651.19）	595.18	7.5%(2.5%—12.9%)	0.0284（0.0186—0.0382）
Philippines	18754473.48（16081014.99-21707058.41）	16273.79	2.15%(1.76%—2.59%)	-0.0083（-0.0086—-0.008）	702200.78（79324.93-1581757.58）	609.09	3.7%(0.9%—6.4%)	-0.0001（-0.0022—0.0021）

Sri Lanka	3716036.99（3161618.06-4382234.56）	15890.54	3.63%(3.05%—4.19%)	0.0248（0.0237—0.0259）	138579.36（16225.29-312066.05）	592.23	1.2%(-3.4%—6.2%)	0.0231（0.0139—0.0323）
Seychelles	17550.82（14923.09-20744.8）	15527.62	1.31%(0.72%—1.90%)	-0.0465（-0.0485—-0.0445）	657.41（78.96-1469.09）	580.57	-7.0%(-13.2%—-0.4%)	-0.0611（-0.0715—-0.0507）
Thailand	12945212.84（11160063.88-15215918.42）	17536.75	-5.53%(-10.57%—-0.32%)	-0.2928（-0.3106—-0.2751）	481835.46（53844.11-1065035.2）	652.45	-2.3%(-9.7%—10.7%)	-0.2719（-0.3047—-0.239）
Timor-Leste	205542.73（172279.55-245709.81）	15767.47	2.69%(2.14%—3.22%)	0.0193（0.0172—0.0215）	7678.03（757.63-17586.01）	588.70	10.2%(5.5%—15.4%)	0.04（0.0254—0.0546）
Viet Nam	16741402.57（14213553.73-19746450.88）	15775.92	2.16%(1.54%—2.83%)	-0.0403（-0.0406—-0.04）	632131.85（72787.19-1418902.36）	594.74	6.4%(2.2%—12.1%)	-0.0208（-0.0251—-0.0165）
**Oceania**	**1864400.1（1571972-2212470.01）**	**14043.19**	**1.16%(0.87%—1.51%)**	**0.0045（0.004—0.0049）**	**68892.29（7961.58-154919.9）**	**519.02**	**1.7%(-2.4%—6.5%)**	**0.0111（0.0062—0.0159）**
American Samoa	7143.89（6082.79-8330.74）	14058.93	0.64%(-0.03%—1.25%)	0.005（0.0032—0.0068）	263.64（33.18-589.27）	518.16	-8.1%(-12.8%—-3.9%)	-0.0203（-0.0325—-0.0081）
Micronesia (Federated States of)	14755.01（12504.39-17328.75）	14078.19	1.33%(0.97%—1.77%)	0.0038（0.0032—0.0044）	546.5（65.44-1233.67）	520.87	0.6%(-3.7%—5.0%)	0.0013（-0.0072—0.0097）
Fiji	132120.48（112104.74-154785.08）	14089.00	0.98%(0.45%—1.60%)	-0.0022（-0.0029—-0.0016）	4886.83（592.98-10905.2）	520.45	-3.1%(-7.7%—1.3%)	-0.0108（-0.0192—-0.0023）
Guam	23087（19812.63-26899.13）	14056.48	1.61%(1.11%—2.15%)	0.0361（0.0334—0.0388）	861.2（114.36-1904.96）	523.53	-5.3%(-10.5%—-1.0%)	0.0181（0.006—0.0302）
Kiribati	16841.17（14230.46-19906.26）	14255.59	1.41%(1.05%—1.80%)	0.0105（0.0096—0.0114）	621.3（72.1-1400.23）	525.75	6.3%(1.2%—11.3%)	0.0275（0.0244—0.0306）
Marshall Islands	8079.43（6824.01-9508.69）	14055.76	1.34%(0.99%—1.75%)	0.003（0.0026—0.0033）	297.84（35.47-665.24）	517.35	-5.6%(-11.0%—-0.2%)	-0.017（-0.0186—-0.0154）
Northern Mariana Islands	7251.17（6207.36-8415.75）	13954.79	1.06%(0.49%—1.68%)	0.0191（-0.019—0.0573）	270.6（36.45-599.11）	519.07	-4.4%(-8.9%—-0.3%)	0.0073（-0.0376—0.0522）
Papua New Guinea	1388463.73（1168289.64-1655045.98）	14021.85	1.37%(0.99%—1.84%)	0.0032（0.0025—0.004）	51278.11（5869.94-115622.84）	518.10	4.3%(-1.1%—10.9%)	0.0216（0.0164—0.0267）
Samoa	27774.82（23530.95-3290	14039.23	1.27%(0.75%—1.80%)	0.0066（0.0063—0.	1026.51（118.57-2307.92	518.98	-2.0%(-6.2%—2.2%)	-0.0036（-0.0159—

	4.71）			0069）	）			0.0087）
Solomon Islands	90518.97 （76290.54-107911.99）	14096.00	1.74%(1.39%—2.14%)	0.0238 （0.0234—0.0241）	3351.56 （376.89-7611.02）	522.13	-0.3%(-5.2%—5.1%)	0.0213 （0.0156—0.0271）
Tonga	13923.49 （11801.44-16407.18）	14199.04	1.36%(0.97%—1.84%)	0.0036 （0.0027—0.0044）	516.61 （61.44-1162.93）	527.09	-4.9%(-9.9%—0.1%)	-0.0116 （-0.0189—-0.0043）
Vanuatu	41663.47 （35126.88-49512.83）	14129.10	1.79%(1.37%—2.25%)	0.0229 （0.0224—0.0235）	1542.35 （178.73-3488.55）	523.44	0.6%(-3.9%—5.0%)	0.0178 （-0.0012—0.0368）
Cook Islands	2631.1 （2261.29-3058.52）	14255.07	1.81%（1.53%—2.05%）	0.0589 （0.0523—0.0655）	97.34 （13.16-214.65）	527.28	1.1%（-2.73%—4.51%）	0.0352 （0.0226—0.0478）
Nauru	1500.44 （1262.88-1789.96）	14158.58	1.4%（1.06%—1.77%）	0.0447 （0.0438—0.0456）	55.43 （6.28-126.00）	522.68	0.82%（-2.72%—4.56%）	0.0272 （0.0137—0.0407）
Niue	244.36 （209.69-284.00）	14087.91	-0.06%(-0.15%—0.02%)	-0.002 （-0.0029—-0.0011）	9.04（1.20-19.92）	520.70	-0.62%（-4.58%—3.1%）	-0.0181 （-0.0257—-0.0105）
Palau	2774.53 （2377.78-3227.85）	13739.06	-2.13%(-2.47%—-1.76%)	-0.0712 （-0.0888—-0.0535）	102.99（14.24，225.87）	508.56	-2.5%（-5.52%—1.28%）	-0.082 （-0.1132—-0.0508）
Tokelau	193.94（164.72，226.23）	14069.17	—0.91% （-1.08%—-0.72%）	-0.0292 （-0.0461—-0.0123）	7.18（0.90-16.09）	521.32	-1.15%（-4.27%—2.63%）	-0.0343 （-0.0564—-0.0123）
Tuvalu	1726.79 （1464.36-2030.77）	13988.66	-2.6%（-2.97%—-2.16%）	-0.0851 （-0.0864—-0.0838）	64.19 （7.85-143.05）	519.83	-2.62%（-6%—1.42%）	-0.0847 （-0.1001—-0.0693）
**North Africa and Middle East**	**98207711.94 （83689909.19-114281975.93）**	**15304.13**	**2.04%(0.40%—3.70%)**	**0.0119 （0.0008—0.023）**	**3843164.9 （678199.99-8368174.04）**	**598.94**	**-1.6%(-4.9%—1.1%)**	**-0.001 （-0.0167—0.0147）**
Afghanistan	4189017.08 （3477675.54-5015573.95）	15219.55	-0.94%(-1.70%—-0.16%)	-0.0857 （-0.0961—-0.0753）	161054.37 （24435.29-362077.81）	585.63	-1.5%(-10.9%—17.2%)	-0.0796 （-0.0998—-0.0595）
Algeria	6774420.06 （5721042.04-7992299.03）	15170.66	1.50%(0.77%—2.27%)	-0.0041 （-0.0046—-0.0036）	265858.3 （47492.77-576873.28）	595.45	-0.1%(-4.2%—4.4%)	-0.0078 （-0.0138—-0.0018）
Bahrain	244147.1 （205557.98-287763.93）	14027.17	-1.33%(-1.83%—-0.82%)	-0.075 （-0.0858—-0.0642）	9642.09 （1786.58-20422.64）	551.51	-5.2%(-9.8%—-0.9%)	-0.0797 （-0.094—-0.0654）
Egypt	16377355.17 （14190620.99-18542239.89）	15670.29	4.81%(-2.04%—12.41%)	0.0701 （0.0648—0.0753）	641568.49 （106040.95-1401926.89）	615.17	1.3%(-7.2%—11.2%)	0.0657 （0.0437—0.0877）
Iran (Islamic	15229950.02	16492.36	3.60%(0.19%—6.98%)	0.0669	593558.97	641.56	1.7%(-2.8%—6.1%)	0.0429

Republic of)	(13250788.84-17521275.76)			(0.0275—0.1064)	(109714-1275796.68)			(0.0088—0.077)
Iraq	6291076.19 (5269686.9-7439825.15)	15038.58	1.12%(0.47%—1.77%)	-0.0108 (-0.0139—-0.0078)	244547.69 (40648.69-539194.1)	584.75	-1.5%(-7.2%—3.5%)	-0.018 (-0.0359—-0.0001)
Jordan	1920533.05 (1610796.7-2269549.3)	14843.49	0.02%(-0.53%—0.62%)	-0.0345 (-0.0397—-0.0293)	75356.33 (12871.42-165494.18)	582.47	-4.3%(-9.1%—-0.2%)	-0.0379 (-0.044—-0.0319)
Kuwait	818071.64 (696347.25-967626.2)	14984.55	4.38%(0.13%—8.76%)	0.1174 (0.1087—0.1262)	31838.22 (5526.85-68170.1)	581.90	-4.8%(-10.9%—1.5%)	0.0901 (0.0783—0.1019)
Lebanon	897712.63 (761686.34-1055678.91)	15238.44	0.74%(-0.13%—1.62%)	-0.0293 (-0.0338—-0.0248)	34908.06 (6348.07-74604.09)	592.73	-4.4%(-10.5%—3.1%)	-0.037 (-0.0585—-0.0156)
Libya	1160232.28 (979112.23-1366496.22)	15082.94	2.51%(1.82%—3.25%)	0.059 (0.0566—0.0615)	45394.34 (8168.74-98359.89)	589.10	-8.5%(-14.1%—-4.3%)	0.0319 (0.0177—0.0462)
Morocco	5872290.23 (4966991.88-6902142.14)	15201.02	1.68%(1%—2.40%)	-0.0119 (-0.013—-0.0107)	228925.34 (41248.93-496937.39)	592.13	-5.5%(-10.3%—-0.9%)	-0.0276 (-0.0409—-0.0142)
Oman	723114.08 (604453.46-855560.96)	14115.28	1.80%(1.20%—2.40%)	0.0095 (0.003—0.0159)	28570.5 (4860.51-60731.74)	557.10	0.6%(-3.8%—5.0%)	0.0092 (-0.0086—0.027)
Palestine	763180.59 (634681.44-905753.81)	15147.20	0.67%(-0.11%—1.42%)	-0.0397 (-0.0414—-0.038)	29676.87 (4856.62-65791.82)	590.22	-5.6%(-11.1%—-0.9%)	-0.054 (-0.0648—-0.0431)
Qatar	476078.81 (396809.82-565821.12)	13503.71	0.58%(0.06%—1.05%)	-0.0039 (-0.0184—0.0106)	18893.34 (3411.84-40347.85)	532.80	-3.5%(-8.2%—1.1%)	-0.016 (-0.0355—0.0034)
Saudi Arabia	6167269.33 (5209389.22-7309686.24)	14115.56	2.33%(1.93%—2.77%)	0.0072 (-0.0021—0.0165)	243297.04 (44016.79-519224.75)	555.39	0.5%(-3.8%—5.5%)	-0.0127 (-0.0183—-0.007)
Sudan	6389369.84 (5319940.61-7585089.46)	15216.83	1.68%(1.13%—2.23%)	-0.0082 (-0.0106—-0.0057)	248997.99 (39572.83-559045.59)	595.27	2.8%(-2.0%—7.9%)	-0.0049 (-0.0139—0.004)
Syrian Arab Republic	2274060.95 (1928131.53-2665063.8)	15609.45	3.79%(2.96%—4.60%)	0.1026 (0.0946—0.1106)	88757.38 (15804.76-193616.08)	606.42	-6.4%(-10.9%—-2.3%)	0.0672 (0.0462—0.0882)
Tunisia	1897431.27 (1617767.16-2219022.81)	15266.51	1.64%(1.31%—1.98%)	0.0146 (0.0141—0.0151)	74123.94 (14142.57-158744.08)	595.23	-8.7%(-14.0%—-3.8%)	-0.0121 (-0.0186—-0.0056)
Türkiye	13291383.92 (11475384.66-15372361.2)	14934.82	2.28%(-2.39%—6.82%)	0.0024 (-0.0003—0.005)	527554.78 (107507.38-1116476.56)	591.36	-1.2%(-7.0%—5.6%)	-0.0056 (-0.028—0.0168)

United Arab Emirates	1535297.11（1288571.48-1850848.61）	13611.91	0.75%(-0.01%—1.56%)	-0.0084（-0.014—-0.0029）	61170.85（11979.71-128408.15）	536.15	-1.7%(-7.6%—2.9%)	-0.0146（-0.027—-0.0021）
Yemen	4824121.81（4022568.32-5730646.53）	15221.95	1.37%(0.97%—1.78%)	0.0025（0.002—0.0029）	185885.46（28722.62-411118.16）	589.63	1.7%(-2.3%—6.3%)	0.0062（-0.0005—0.0129）
**South Asia**	**286372319.57（243859319.05-328165147.58）**	**14859.95**	**2.17%(-0.96%—5.08%)**	**0.0119（0.0066—0.0172）**	**10362368.63（1252047.21-23272970.57）**	**537.87**	**6.3%(1.8%—10.4%)**	**0.0208（0.0159—0.0257）**
Bangladesh	24942553.55（21005792.71-29258180.82）	14622.91	3.69%(3.15%—4.26%)	0.0425（0.0414—0.0437）	903347.14（108412.1-2037191.62）	529.83	8.4%(2.7%—14.1%)	0.0515（0.0408—0.0621）
Bhutan	117267.07（98755.74-138490.8）	14430.27	3.15%(2.35%—4.06%)	0.0128（0.0083—0.0173）	4273.88（499.58-9727.15）	526.35	7.0%(0.4%—15.2%)	0.0282（0.0232—0.0332）
India	223124203.28（189835219.6-254668065.16）	14909.22	1.88%(-2.08%—5.54%)	0.0114（0.0085—0.0143）	8096330.87（1015745.36-18217286.59）	540.99	7.4%(2.2%—12.7%)	0.029（0.0209—0.0372）
Nepal	4977231.95（4192665.7-5913099.14）	15717.90	4.05%(0.19%—8.27%)	0.033（0.031—0.0349）	180025.09（20901.71-408340.36）	568.37	8.8%(0.6%—17.1%)	0.0475（0.0347—0.0602）
Pakistan	33211063.73（27967357.92-38280810.58）	14570.79	2.49%(1.90%—3.10%)	0.0012（-0.0083—0.0107）	1178391.64（107593.49-2697294.48）	517.34	-2.9%(-17.9%—1.0%)	-0.0535（-0.0735—-0.0335）
**Southern Sub-Saharan Africa**	**10526173.86（9015592.98-12175770.27）**	**12876.73**	**1.69%(1.34%—2.06%)**	**-0.0077（-0.0083—-0.0071）**	**389158.81（65892.73-848009.31）**	**477.47**	**-9.8%(-13.1%—-7.0%)**	**-0.0434（-0.0504—-0.0363）**
Botswana	309365.49（259828.62-364189.16）	12415.02	1.17%(0.57%—1.80%)	-0.0308（-0.0313—-0.0302）	11451.71（1873.25-25553.4）	461.18	-8.4%(-13.8%—-3.1%)	-0.0591（-0.0757—-0.0424）
Lesotho	230765.83（193158.44-273822.12）	12483.69	0.29%(-0.36%—0.89%)	-0.0721（-0.0731—-0.0711）	8442.8（1319.23-18878.62）	458.70	-19.7%(-24.7%—-15.5%)	-0.137（-0.1521—-0.1218）
Namibia	298885.33（250471.6-354099.58）	12478.70	2.45%(1.86%—3.03%)	0.0062（0.0057—0.0068）	11093.72（1763.99-24924.2）	465.84	-4.5%(-10.1%—1.2%)	-0.0137（-0.0221—-0.0053）
South Africa	7754001.63（6677175.71-8973176.43）	12990.88	1.42%(1.06%—1.88%)	-0.0132（-0.0141—-0.0124）	286548.76（49985.77-622368.74）	480.97	-10.6%(-13.9%—-7.8%)	-0.0534（-0.0585—-0.0484）
Eswatini	139976.18（116936.7-166126.26）	12480.77	1.49%(0.97%—2.07%)	-0.0235（-0.0248—-0.0222）	5129.65（794.57-11639.24）	460.13	-18.4%(-23.6%—-13.4%)	-0.0943（-0.1074—-0.0812）
Zimbabwe	1793179.4（1493089.51-21	12527.60	2.75%(2.23%—3.31%)	0.0228（0.0221—0.	66492.17（10158.56-1516	467.74	-3.7%(-9.9%—1.5%)	0.0114（0.0025—

	34515.29）			0236）	65.47）			0.0202）
**Western Sub-Saharan Africa**	**64362036.94（54002945.39-76098769.29）**	**14870.35**	**1.61%(-0.49%—3.49%)**	**0.0156（0.0141—0.0172）**	**2380161.24（312311.14-5415290.49）**	**554.49**	**8.2%(4.4%—12.0%)**	**0.0375（0.0333—0.0417）**
Benin	1711349.62（1430768.98-2065088.85）	14559.28	0.42%(0.10%—0.76%)	-0.0212（-0.0221—-0.0203）	63208.85（8047.05-144774.32）	542.17	10.0%(4.0%—16.4%)	0.0073（-0.0004—0.015）
Burkina Faso	2863363.03（2391127.27-3457629.19）	14610.82	0.68%(0.37%—1.03%)	-0.0136（-0.0141—-0.0131）	106089.4（13364.76-243014.18）	546.10	13.2%(5.3%—21.7%)	0.0227（0.0023—0.0431）
Cameroon	4184985.44（3502856.11-5051871.32）	14498.08	1.41%(1.04%—1.80%)	-0.0131（-0.0133—-0.0128）	154722.15（19198.34-354562.89）	539.80	7.2%(1.1%—13.7%)	0.0089（-0.0097—0.0274）
Cabo Verde	85966.35（72988.86-101828.62）	14422.31	0.45%(-0.32%—1.18%)	-0.0789（-0.0802—-0.0776）	3211.92（466.38-7149.39）	539.81	-2.9%(-10.0%—3.0%)	-0.0833（-0.0925—-0.0741）
Chad	2076158.71（1720186.15-2510071.49）	14502.42	-0.18%(-0.45%—0.09%)	-0.0286（-0.0291—-0.0281）	76369.34（9317.21-176525.93）	538.42	3.2%(-2.2%—8.5%)	-0.0258（-0.0358—-0.0159）
Côte d'Ivoire	3662068.89（3064786.78-4430446.1）	14352.21	1.58%(1.06%—2.11%)	-0.0002（-0.0007—0.0003）	135560.58（17094.02-309402.14）	534.85	16.1%(8.6%—24.1%)	0.0478（0.0349—0.0607）
Gambia	319385.29（267438.61-385393.97）	14530.02	2.27%(1.91%—2.78%)	0.0255（0.0243—0.0267）	11750.38（1467.24-26671.26）	538.73	-2.4%(-7.9%—3.4%)	0.0129（0.0012—0.0246）
Ghana	4760614.3（3992648.62-5745529.96）	14609.79	2.21%(1.86%—2.60%)	0.0187（0.0178—0.0195）	176558.38（23094.52-399474.89）	545.15	6.2%(0.5%—12.2%)	0.0334（0.0203—0.0465）
Guinea	1712115.65（1431286.44-2067418.43）	14621.66	0.99%(0.73%—1.31%)	0.0022（0.001—0.0034）	63178.74（8033.16-144227.56）	544.31	2.4%(-3.8%—9.1%)	0.0106（-0.0043—0.0255）
Guinea-Bissau	270549.95（226456.5-326698.22）	14614.35	1.23%(0.94%—1.55%)	-0.0037（-0.0041—-0.0033）	9968.82（1255.73-22870.81）	542.99	6.2%(-0.6%—13.0%)	0.0064（-0.0125—0.0254）
Liberia	741785.32（619373.38-896169.36）	14413.91	0.68%(0.27%—1.07%)	-0.0128（-0.0349—0.0092）	26958.52（3441.14-60800.83）	526.60	7.8%(2.4%—14.8%)	0.0117（0.0004—0.023）
Mali	2924072.65（2427810.03-3538302.17）	14516.45	0.40%(0.20%—0.60%)	-0.0114（-0.013—-0.0098）	107593.93（13171.24-245928.84）	538.85	3.3%(-2.8%—9.0%)	-0.0018（-0.0104—0.0069）
Mauritania	576441.12（481549.75-694367.87）	14556.42	1.88%(1.50%—2.29%)	0.0046（0.0042—0.0049）	21417.08（2788.71-49077.08）	545.08	5.6%(-0.5%—11.4%)	0.0155（0.0066—0.0244）
Niger	2919359.24	14539.47	0.87%(0.64%—1.19%)	0.0115	108020.05	543.59	8.7%(3.1%—15.0%)	0.0352

	（2418795.4-3530584.13）			（0.0107—0.0124）	（12823.77-247684.76）			（0.0273—0.043）
Nigeria	31067866.75 （26146877.16-36096317.49）	15259.08	2.31%(-1.89%—6.38%)	0.0399 （0.0365—0.0432）	1149585.07 （154649.84-2624356.36）	569.67	9.3%(3.8%—15.3%)	0.0646 （0.057—0.0723）
Sao Tome and Principe	30685.36 （25661.4-37053.51）	14456.37	1.27%(0.74%—1.80%)	-0.0219 （-0.0227—-0.021）	1144.54 （153.15-2593.98）	542.03	0.8%(-5.3%—7.0%)	-0.0208 （-0.0284—-0.0132）
Senegal	2124776.01 （1778975.04-2557698.59）	14508.51	1.03%(0.70%—1.37%)	-0.0125 （-0.0133—-0.0117）	78587.73 （10289.54-178783.77）	540.26	4.2%(-2.9%—11.5%)	0.005 （-0.0034—0.0135）
Sierra Leone	1188125.46 （996359.29-1432068.18）	14478.41	0.76%(0.31%—1.18%)	-0.0097 （-0.0154—-0.004）	43906.96 （5565.78-100909.21）	539.02	7.0%(1.4%—12.5%)	0.0089 （-0.0077—0.0254）
Togo	1141616.82 （955308.01-1378419.43）	14598.14	1.12%(0.81%—1.47%)	-0.0052 （-0.0059—-0.0045）	42301.01 （5772.37-96101.02）	544.41	5.2%(-1.1%—12.0%)	0.0101 （0.0026—0.0176）
**Eastern Sub-Saharan Africa**	**34295581.08 （28749229.91-40314574.24）**	**9084.26**	**2.45%(1.40%—3.61%)**	**0.0326 （0.0301—0.0351）**	**1292755.84 （253046.25-2954612.51）**	**347.19**	**8.8%(3.8%—12.2%)**	**0.04 （0.0325—0.0474）**
Burundi	1035656.53 （866198.24-1224075.6）	9028.71	-0.28%(-0.74%—0.22%)	-0.0516 （-0.0547—-0.0484）	39199.15 （7847.21-89648.5）	347.39	3.6%(-2.8%—10.0%)	-0.045 （-0.0599—-0.0301）
Comoros	66936.17 （56466.53-78294.58）	9086.74	1.50%(0.91%—2.20%)	-0.0141 （-0.0145—-0.0136）	2573.01 （557.07-5738.14）	351.44	3.8%(-5.2%—13.2%)	-0.006 （-0.0231—0.0111）
Djibouti	112428.98 （95034.47-131863.25）	8909.93	1.44%(0.91%—2%)	-0.0173 （-0.0228—-0.0119）	4318.46 （909.44-9685.1）	345.08	0.2%(-5.6%—6.6%)	-0.0161 （-0.0278—-0.0044）
Eritrea	557907.98 （468792.03-657810.72）	9085.39	0.83%(0.41%—1.24%)	-0.0295 （-0.0305—-0.0285）	21234.27 （4352.76-48467.75）	349.48	15.0%(7.9%—22.0%)	0.004 （-0.0121—0.0202）
Ethiopia	8145627.79 （6882390.89-9470613.33）	8365.36	1.92%(-0.59%—4.54%)	0.0159 （0.0128—0.019）	301709.85 （53833.98-693817.61）	313.55	9.3%(-0.8%—14.7%)	-0.0117 （-0.0213—-0.0021）
Kenya	4547282.14 （3823894.04-5275130.63）	9671.53	1.48%(1.24%—1.74%)	0.0023 （0.0018—0.0028）	173532.56 （35638.52-387907.29）	373.46	1.0%(-4.0%—4.1%)	0.0011 （-0.0045—0.0067）
Madagascar	2351165.43 （1969322.58-2787780.89）	9122.41	2%(1.71%—2.32%)	0.0112 （0.0109—0.0115）	89242.73 （17765.32-204905.19）	351.38	7.8%(1.5%—13.7%)	0.0277 （0.0107—0.0448）
Malawi	1593524.75 （1331646.6-1890612.93）	9155.39	1.96%(1.51%—2.38%)	0.0103 （0.0081—0.0126）	60326.85 （11597.64-140067.62）	351.95	12.4%(4.8%—19.7%)	0.0425 （0.0239—0.061）

Mozambique	2404741.39（2008691.22-2847747.2）	9206.13	1.17%(0.85%—1.52%)	-0.0039（-0.005—-0.0029）	89499.48（17780.81-203346.2）	347.92	0.6%(-6.1%—7.7%)	-0.0079（-0.0246—0.0088）
Rwanda	1140900.56（957536.25-1346121.47）	9178.77	2.10%(1.74%—2.52%)	0.0004（-0.0064—0.0073）	43387.03（8833.26-97525.24）	353.02	4.7%(-1.7%—10.7%)	0.0065（-0.007—0.02）
Somalia	1599393.28（1335019.42-1895441.67）	9098.36	0.62%(0.48%—0.78%)	0.0154（0.0134—0.0175）	60301.86（11477.13-138448.98）	349.03	1.2%(-4.9%—7.7%)	0.0233（0.0107—0.036）
South Sudan	759706.12（635941.54-892834.52）	9129.91	3.24%(2.77%—3.69%)	0.0785（0.0748—0.0823）	28640.19（5590.93-65407.61）	347.88	4.1%(-1.7%—9.9%)	0.0797（0.0671—0.0923）
United Republic of Tanzania	4860792.73（4038448.94-5780151.93）	9368.60	6.06%(0.68%—11.63%)	0.1376（0.1251—0.15）	184988.18（36396.68-421095.89）	361.14	19.9%(9.9%—29.7%)	0.1707（0.139—0.2024）
Uganda	3340425.47（2789408.35-3959229.39）	9175.87	2.15%(1.85%—2.49%)	0.0204（0.0196—0.0213）	126088.97（24580.33-293840.84）	352.24	14.7%(7.6%—22.5%)	0.0566（0.0451—0.0681）
Zambia	1749234.38（1450044.86-2087025.79）	10080.75	1.32%(0.69%—1.98%)	-0.0107（-0.0314—0.0101）	66587.78（14069.93-150830.79）	390.81	3.2%(-3.9%—11.4%)	0.0139（-0.008—0.0358）
**Central Sub-Saharan Africa**	**15114474.24（12560503.64-17911094.32）**	**12396.15**	**0.56%(0.38%—0.76%)**	**-0.0088（-0.0092—-0.0085）**	**557712.62（84070.28-1267911.2）**	**461.37**	**10.4%(5.2%—15.3%)**	**0.0191（0.0088—0.0295）**
Angola	3462968.97（2878204.34-4111642.23）	12500.79	2.72%(2.34%—3.10%)	0.044（0.0431—0.0448）	127821.33（18995.35-293838.74）	465.75	3.8%(-2.5%—10.4%)	0.0443（0.0352—0.0535）
Central African Republic	618175.68（514266.28-733541.46）	12462.33	0.47%(0.26%—0.66%)	0.0014（-0.0004—0.0031）	22753.76（3455.62-51401.34）	461.90	7.1%(1.4%—13.8%)	0.0278（0.0131—0.0426）
Congo	651923.49（545310.03-773289.01）	12387.53	0.20%(-0.13%—0.50%)	-0.0194（-0.0198—-0.0189）	24191.05（3795.02-54393.32）	462.17	1.5%(-4.4%—7.1%)	-0.0177（-0.0269—-0.0085）
Democratic Republic of the Congo	9986213.01（8297767-11847755.53）	12358.98	-0.12%(-0.32%—0.10%)	-0.0243（-0.0248—-0.0238）	368336.9（55578.98-835163.56）	459.87	12.9%(6.6%—19.6%)	0.0163（0.0025—0.0302）
Equatorial Guinea	175468.24（145548.77-209982.41）	12281.69	-0.09%(-0.89%—0.68%)	-0.0788（-0.0827—-0.075）	6476.09（936.69-14750.3）	457.66	10.8%(2.1%—19.8%)	-0.0382（-0.0479—-0.0285）
Gabon	219724.84（184135.2-260555.21）	12471.35	2.03%(1.45%—2.54%)	0.0239（0.0227—0.0251）	8133.51（1285.27-18262.13）	463.95	1.3%(-4.8%—8.1%)	0.0225（0.0112—0.0338）

Supplementary Table 2. Prevalent Cases and YLDs for Tension-Type Headache in 2021, Percentage Change in Age-Standardized Rates by Location, and Average Annual Percentage Change

	Tension-type headache							
	Prevalence				YLD			
	2021 counts	The age-standardized prevalence rate	Percentage change in age-standardised rates, 1990–2021	AAPC	2021 counts	The age-standardized YLD rate	Percentage change in age-standardised rates, 1990–2021	AAPC
**Global**	**2011612877.49 （1776544390.83-2270860638.85）**	**24764.77**	**1.0%(0.2%—1.7%)**	**-0.0141 （-0.027—-0.0012）**	**4596785.25 （1347300.84-15012932.75）**	**55.69**	**-1.4%(-5.2%—2.3%)**	**-0.0733 （-0.0813—-0.0652）**
High SDI	362971307.86 （323194543.63-402243512.05）	30603.28	-0.9%(-2.1%—0.2%)	-0.0633 （-0.0699—-0.0568）	840608.35 （246202.29-2813491.75）	66.79	-6.2%(-11.3%—-2.9%)	-0.0484 （-0.0546—-0.0423）
High-middle SDI	336588442.89 （298434183.88-376777242.37）	23862.61	1.8%(0.2%—3.3%)	-0.0238 （-0.0439—-0.0036）	908608.93 （292109.64-2657935.17）	59.93	-2.0%(-7.4%—2.4%)	-0.1588 （-0.1868—-0.1308）
Middle SDI	599794101.09 （529384315.81-676818455.65）	23378.72	8.4%(7.2%—9.7%)	0.1892* （0.159—0.2195）	1362751.76 （402787.05-4457145.25）	51.54	5.1%(-2.3%—15.2%)	0.1507 （0.1207—0.1807）
Low-middle SDI	481761334.53 （419781066.26-552026462.92）	25022.79	2.0%(1.6%—2.5%)	0.0018 （-0.0035—0.0071）	999043.15 （274414.94-3523211.62）	52.99	5.5%(-1.3%—13.0%)	0.0296 （0.0207—0.0386）
Low SDI	228819981.11 （195204790.1-266944574.98）	22780.22	0.2%(-0.2%—0.6%)	-0.0346 （-0.0443—-0.025）	481767.17 （134432.76-1711203.56）	51.76	7.5%(1.8%—13.3%)	0.005 （-0.0041—0.014）
**High-income North America**	**135183092.58 （121040106.2-149990906.82）**	**34180.26**	**-2.5%(-5.0%—0.5%)**	**-0.0801 （-0.0829—-0.0773）**	**290084.27 （79671.12-1014112.13）**	**70.01**	**-13.4%(-18.2%—-8.8%)**	**-0.0586 （-0.0641—-0.0531）**
Canada	13236291.54 （11595928.59-14927389.12）	32911.94	0.4%(-0.1%—1.0%)	-0.0017 （-0.0021—-0.0013）	28647.48 （7501.25-99937.67）	67.31	-4.8%(-14.4%—5.5%)	0.0022 （-0.0124—0.0167）
Greenland	19577.58 （17086.95-22286.55）	32837.19	1.0%(0.4%—1.6%)	0.005 （0.0028—0.0073）	41.31 （10.89-148.25）	66.44	1.8%(-10.0%—16.8%)	0.0358 （0.0255—0.046）
United States of America	121925103.6 （109446201.37-135070007.56）	34320.58	-2.8%(-5.6%—0.4%)	-0.0884 （-0.0913—-0.0855）	261390.93 （72044.45-915566.57）	70.32	-14.2%(-18.9%—-9.6%)	-0.0643 （-0.0693—-0.0592）
**Australasia**	**8987985.7 （7908018.77-10153456.84）**	**27215.52**	**-0.3%(-1.2%—0.7%)**	**-0.0007 （-0.0017—0.0004）**	**21150.24 （6191.25-67175.8）**	**60.99**	**2.4%(-6.0%—10.9%)**	**0.0163 （0.0144—0.0181）**
Australia	7420120.93 （6494827.73-83	26964.15	-0.6%(-1.6%—0.6%)	-0.0003 （-0.0007—0	17462.93 （5142.89-55575.	60.38	1.7%(-8.1%—12.4%)	0.0145 （0.0121—

	99360.45）			.0001）	02）			0.017）
New Zealand	1567864.77（1403040.1-1746377.82）	28452.74	1.0%(-0.1%—2.0%)	-0.0047（-0.0136—0.0041）	3687.31（1089.25-11520.88）	64.05	6.0%(-2.9%—15.6%)	0.0199（0.0058—0.0339）
**High-income Asia Pacific**	**60741993.83（54074164.81-67510418.69）**	**29867.61**	**3.7%(1.1%—6.6%)**	**0.0267（-0.0133—0.0668）**	**139920.2（40610.47-440967.24）**	**63.72**	**2.3%(-2.6%—7.6%)**	**0.0087（0.0022—0.0151）**
Brunei Darussalam	141965.15（122121.7-164824.32）	28928.90	2.1%(1.4%—3.0%)	0.003（0.0016—0.0045）	303.29（81.68-1044.57）	60.40	0.6%(-9.8%—13.4%)	0.0194（0.012—0.0268）
Japan	41142903.91（36598391.65-45397123.31）	29705.70	1.6%(-1.1%—5.0%)	-0.0311（-0.0334—-0.0287）	96075.16（28637.8-300063.03）	64.56	1.3%(-3.3%—5.6%)	-0.001（-0.0069—0.0048）
Singapore	1808096.24（1577179.13-2063794.9）	28481.36	8.3%(3.1%—14.3%)	0.2644（0.2486—0.2801）	4156.48（1219.61-14018.4）	59.68	7.7%(-7.2%—19.6%)	0.1398（0.1101—0.1695）
Republic of Korea	17649028.52（15449503.81-19886009.48）	30412.35	11.2%(1.4%—22.5%)	0.1318（0.0758—0.1878）	39385.27（10774.25-130193.59）	62.39	8.8%(-6.5%—25.4%)	0.0845（0.0669—0.102）
**Western Europe**	**153919251.89（136293091.3-171722574.01）**	**32770.54**	**1.5%(0.6%—2.4%)**	**0.0086（0.0009—0.0162）**	**360779.02（103682.91-1263384.07）**	**71.93**	**-0.9%(-6.9%—3.0%)**	**0.0113（-0.016—0.0386）**
Andorra	31423.49（27452.8-35599.97）	32352.91	0.7%(0.1%—1.2%)	0.0036（0.0013—0.0059）	73.15（19.82-260.48）	69.04	-3.5%(-13.8%—6.3%)	0.0303（0.0023—0.0583）
Austria	3103357.82（2715444.43-3487542.35）	31759.67	0.6%(0.1%—1.3%)	-0.0024（-0.0054—0.0006）	7443.98（2141.41-26586.7）	71.22	0.0%(-11.7%—11.0%)	-0.0141（-0.0381—0.01）
Belgium	3969616.84（3489453.36-4488578.92）	32396.92	0.4%(-0.5%—1.3%)	-0.0011（-0.0017—-0.0005）	8994.79（2463.24-32639.7）	69.42	-3.6%(-14.6%—8.2%)	0.0021（-0.0017—0.0058）
Cyprus	486411.42（421730.38-553681.48）	32411.95	0.9%(0.4%—1.6%)	0.0016（0.001—0.0021）	1111.01（302.23-3982.28）	69.91	0.2%(-12.0%—10.9%)	0.0288（0.0201—0.0375）
Denmark	2116005.52（1858506.23-2388872.96）	33764.78	-1.9%(-6.0%—2.1%)	-0.0824（-0.0889—-0.076）	4632.65（1204.37-17116.71）	70.36	0.6%(-12.4%—15.1%)	-0.0307（-0.0523—-0.0092）
Finland	1917833.38（1686801.86-2164471.63）	32383.20	0.1%(-0.7%—0.7%)	-0.0041（-0.0047—-0.0036）	4336.41（1198.56-15480.41）	69.26	-0.3%(-10.5%—10.0%)	-0.0072（-0.023—0.0086）
France	21984828.03（19299481.34-24782861.99）	31418.48	0.9%(0.1%—1.8%)	-0.004（-0.0205—0.0125）	55822.97（16584.78-177931.9）	74.92	-0.7%(-10.2%—8.4%)	0.0103（-0.028—0.0486）
Germany	30566291.99	33107.73	3.2%(-0.8%—7.5%)	0.0615	71616.46	72.21	-2.5%(-16.0%—7.7%)	-0.0044

	（26922507.68-34354087.07）			（0.041—0.0819）	（20869.31-238118.17）			（-0.0386—0.0298）
Greece	3570883.28 （3140584.3-4030948.66）	32408.70	2.0%(1.3%—2.7%)	-0.0002 （-0.0007—0.0002）	8272.06 （2284.34-29200.64）	69.65	-3.7%(-16.8%—9.4%)	0.009 （-0.0037—0.0217）
Iceland	120532.39 （105593.34-136816.06）	32368.67	1.0%(0.3%—1.7%)	-0.0021 （-0.0027—-0.0015）	269.32 （73.19-973.8）	69.37	0.5%(-10.3%—11.4%)	0.0019 （-0.0223—0.0262）
Ireland	1696845.31 （1488430.77-1916001.36）	32406.02	0.9%(0.3%—1.5%)	0 （-0.0002—0.0003）	3837.44 （1033.02-13761.09）	69.58	-2.8%(-14.4%—7.5%)	0.0147 （0.0016—0.0277）
Israel	3094744.19 （2703221.12-3511141.26）	32400.54	1.1%(0.6%—1.8%)	-0.0019 （-0.0024—-0.0014）	6753.62 （1761.98-24927.52）	69.80	-1.0%(-10.6%—8.5%)	-0.0073 （-0.023—0.0083）
Italy	21839653.34 （19560998.04-23983501.55）	33572.31	1.1%(-1.0%—3.1%)	-0.0019 （-0.1237—0.12）	50176.42 （14083.58-183683.64）	71.46	3.2%(-4.1%—10.4%)	0.0227 （-0.0698—0.1152）
Luxembourg	228971.26 （201257.6-258806.73）	32279.96	1.0%(0.4%—1.6%)	-0.0037 （-0.0109—0.0035）	569.87 （173.89-1852.64）	74.98	2.0%(-8.9%—11.6%)	0.0011 （-0.027—0.0292）
Malta	154171.27 （135412.36-173757.3）	32376.71	0.8%(0.2%—1.3%)	-0.0037 （-0.0044—-0.003）	352.83 （96.9-1261.74）	69.25	-3.4%(-16.3%—8.1%)	-0.0172 （-0.0201—-0.0143）
Netherlands	6476061.47 （5683170.58—7328480.75）	34984.91	0.1%(-0.8%—0.9%)	0.0011 （-0.0264—0.0286）	13226.68 （3311.93—54846.54）	68.16	-4.5%(-18.1%—9.1%)	0.0014 （-0.0354—0.0382）
Norway	2063243.43 （1850471.07-2279055.63）	35492.44	1.6%(-1.5%—4.4%)	0.0343 （0.0305—0.0381）	4344.01 （1107.11-16723.17）	71.25	6.3%(-3.7%—16.4%)	0.111 （0.0929—0.1292）
Portugal	3747590.35 （3292299.65-4229981.77）	32429.23	1.4%(0.8%—2.1%)	-0.0009 （-0.0013—-0.0005）	8715.95 （2435.1-30614.89）	69.80	1.7%(-10.9%—13.2%)	0.0212 （0.009—0.0335）
Spain	15640493.7 （13676285.21-17704584.91）	31535.63	1.0%(0.3%—1.8%)	-0.0019 （-0.0091—0.0054）	39938.92 （11640.26-133191.27）	73.69	-1.2%(-12.7%—12.3%)	0.0302 （-0.038—0.0985）
Sweden	3771717.69 （3381333.58-4156615.55）	34297.27	0.7%(-1.8%—3.2%)	0.0022 （0.0005—0.004）	7850.08 （2084.21-28634.82）	68.31	-2.3%(-11.7%—7.0%)	-0.0053 （-0.0252—0.0146）
Switzerland	2884248.94 （2516060.47-3265068.87）	29829.20	5.8%(0.9%—12.0%)	0.1449 （0.1375—0.1523）	6877.39 （1979.57-23037.84）	66.36	5.4%(-7.7%—17.7%)	0.0812 （0.0615—0.1009）
United Kingdom	24294167.52 （21795300.59-26912634.21）	33487.02	1.4%(0.9%—1.8%)	0.0004 （-0.0001—0.0009）	55187.88 （15436.92-197681.55）	72.27	-2.7%(-9.3%—0.6%)	0.0089 （0.0064—0.0115）

Monaco	13089.19 (11419.18-14774.06)	32411.21	-0.06%（-0.47%—0.4%）	-0.0018（-0.0024—-0.0013）	30.62 (8.57-108.75)	69.94	0.01%（-10.19%—13.53%）	-0.0023（-0.0152—0.0106）
San Marino	11550.38 (10155.90-13020.42)	32430.84	0.08%（-0.34%—0.52%）	0.0026（0.0022—0.003）	26.84 (7.54-95.17)	70.07	0.79%（-8.88%—12.8%）	0.0257（0.0132—0.0381）
**Southern Latin America**	**18855579.24（16364520.66-21586397.96）**	**26264.27**	**2.6%(1.2%—4.0%)**	**0.0244（0.0233—0.0255）**	**44203.43（12332.92-143419.08）**	**59.96**	**-1.9%(-10.5%—5.9%)**	**0.0164（-0.0047—0.0376）**
Argentina	12609503.34（10934965.43-14451413.25）	26270.96	1.9%(0.9%—2.9%)	-0.0007（-0.0011—-0.0003）	29402.85（8173.11-95668.22）	60.02	-2.1%(-12.3%—8.7%)	0.0053（-0.0223—0.0329）
Chile	5297829.33（4624021.84-6065549.96）	26244.34	4.8%(0.4%—9.8%)	0.0986（0.0951—0.1021）	12547.33（3534.92-40314.55）	59.83	0.1%(-12.0%—11.1%)	0.0454（0.0269—0.064）
Uruguay	947205.93（827164.57-1075431.91）	26281.32	1.3%(0.3%—2.2%)	0.0005（0.0002—0.0007）	2250.82（669.85-7304.93）	60.02	-5.5%(-14.9%—5.3%)	0.0031（-0.0038—0.0101）
**Eastern Europe**	**69475455.82（62206601.56-76924171.47）**	**31243.12**	**1.9%(0.0%—3.9%)**	**0.01（0.0056—0.0144）**	**240060.24（86665.76-660402.84）**	**96.83**	**0.5%(-4.6%—4.2%)**	**0.0111（0.0015—0.0208）**
Belarus	3032617.46（2670381.18-3446845.49）	30340.65	1.5%(0.9%—2.2%)	-0.0005（-0.001—0）	9860.82（3328.76-28254.23）	88.01	-2.2%(-10.3%—7.5%)	0.001（-0.0104—0.0124）
Estonia	423964.58（373686.42-481706.88）	30335.97	1.9%(1.3%—2.5%)	-0.0009（-0.0014—-0.0005）	1350（466.04-3954.34）	87.75	1.2%(-7.8%—10.1%)	-0.0095（-0.0224—0.0033）
Latvia	602991.7（532225.52-685044.74）	30338.15	1.6%(0.8%—2.3%)	-0.0007（-0.001—-0.0005）	1952.02（673.71-5673.06）	87.86	1.9%(-9.0%—11.0%)	-0.0017（-0.0085—0.0052）
Lithuania	924765.79（816741.2-1037971.84）	31729.84	2.3%(1.4%—3.3%)	0.0138（0.0016—0.0259）	2618.43（870.43-8137.19）	81.03	-2.0%(-12.4%—7.5%)	0.0012（-0.0123—0.0147）
Republic of Moldova	1202123.83（1055850.88-1368268.37）	30334.65	1.9%(1.2%—2.7%)	-0.0005（-0.0011—0）	3869.56（1331.83-11073.73）	87.90	2.3%(-7.3%—10.1%)	-0.0071（-0.0183—0.004）
Russian Federation	48225991.18（43156672.65-53273636.2）	31147.27	2.3%(-0.5%—5.2%)	0.0174（0.0105—0.0243）	171991.22（63374.06-460776.63）	99.72	0.7%(-4.5%—4.9%)	0.0034（-0.0052—0.012）
Ukraine	15063001.3（13449448.58-16721250.84）	31872.74	1.0%(0.4%—1.5%)	-0.0008（-0.001—-0.0007）	48418.19（16949.09-137600.83）	91.72	-1.4%(-9.1%—6.2%)	-0.0045（-0.0142—0.0052）
**Central Europe**	**38175566.57（33813707.07-4**	**30488.91**	**2.3%(1.9%—2.8%)**	**0.0027（0.0015—0.**	**101909.16（32757.21-3107**	**74.70**	**3.2%(-3.4%—8.2%)**	**0.0185（0.0146—**

	**2723788.04）**			**0039）**	**11.1）**			**0.0223）**
Albania	864333.17（756269.08-980673.58）	29929.00	2.8%(2.0%—3.6%)	-0.0008（0.0001—0.0014）	2251.74（715.54-7082.91）	72.99	1.8%(-8.7%—12.1%)	0.0351（0.0236—0.0466）
Bosnia and Herzegovina	1077398.6（944877.29-1215194.37）	29958.94	2.5%(1.8%—3.3%)	-0.0013（-0.0024—-0.0003）	2867.17（931.51-8679.88）	72.69	-1.1%(-13.3%—8.5%)	0.0092（-0.0071—0.0255）
Bulgaria	2209582.32（1929372.7-2478332.4）	29955.10	1.5%(0.9%—2.2%)	-0.0011（-0.0014—-0.0008）	5906.65（1871.44-17850.31）	72.81	-1.5%(-12.4%—7.9%)	0.0018（-0.0209—0.0245）
Croatia	1372487.35（1199050.64-1544565.33）	29953.92	2.3%(-1.7%—6.0%)	-0.0072（-0.0809—0.0666）	3657.1（1163.29-11350.3）	73.08	1.2%(-9.4%—11.0%)	0.0023（-0.0188—0.0233）
Czechia	3421199.19（2998773.73-3833393.42）	29944.15	1.7%(1.1%—2.3%)	-0.0047（-0.0051—-0.0043）	9054.47（2849.09-27930.03）	72.75	0.7%(-9.6%—10.2%)	-0.0039（-0.0158—0.0079）
Hungary	3127052.91（2742043.15-3503148.54）	29976.16	2.1%(1.4%—2.9%)	-0.0022（-0.0028—-0.0017）	8340.21（2696.69-25492.29）	73.07	6.0%(-4.6%—16.6%)	0.0169（0.0071—0.0266）
North Macedonia	718468.34（631223.15-814777.23）	29923.19	2.4%(1.7%—3.1%)	-0.0028（-0.0034—-0.0022）	1892.63（606.06-5825.83）	72.65	-2.9%(-13.2%—7.0%)	0.0015（-0.0055—0.0084）
Montenegro	198591.57（174528.53-224394.71）	29955.13	1.7%(1.1%—2.4%)	-0.0014（-0.0022—-0.0006）	520.5（164.52-1621.39）	73.04	-5.7%(-16.0%—4.4%)	-0.0024（-0.0091—0.0043）
Poland	13140254.87（11726101.46-14550075.33）	31533.24	1.8%(1.3%—2.3%)	-0.0029（-0.0035—-0.0023）	35440.63（11577.11-107825.89）	78.06	4.1%(-3.6%—9.6%)	0.0176（0.0126—0.0226）
Romania	6117197.6（5362639.88-6865043.34）	29957.47	2.5%(1.9%—3.2%)	-0.0019（-0.0024—-0.0014）	16296.43（5103.52-49964）	73.08	5.4%(-5.8%—15.3%)	0.0185（0.0118—0.0253）
Serbia	2937511.06（2582145.71-3314003.05）	29928.17	3.2%(2.4%—4.0%)	-0.0041（-0.005—-0.0033）	7706.93（2462.84-23767.31）	73.08	-0.3%(-10.7%—8.5%)	0.007（-0.0009—0.0149）
Slovakia	1768339.92（1551603.5-1994579.56）	29962.05	1.8%(1.2%—2.4%)	-0.003（-0.0033—-0.0027）	4705.6（1515.18-14451.14）	73.13	2.5%(-8.1%—13.5%)	0.0099（0.0052—0.0147）
Slovenia	667491.14（581256.3-748537.64）	29927.39	1.7%(1.0%—2.4%)	-0.0073（-0.0081—-0.0065）	1785.77（582.53-5402.57）	72.89	3.5%(-6.9%—14.1%)	0.0011（-0.0171—0.0193）
**Central Asia**	**29097629.69（25144968.37-33291691.11）**	**30274.55**	**1.6%(1.2%—1.9%)**	**-0.0017（-0.002—-0.0015）**	**64830.56（17776.03-228393.97）**	**66.89**	**0.5%(-5.6%—5.8%)**	**-0.0041（-0.0102—0.002）**
Armenia	967732.16	30276.13	1.6%(1.1%—2.2%)	-0.0009	2265.77	67.18	1.5%(-10.2%—11.8%)	0.0114

	（843392.46-1098759.41）			（-0.0014—-0.0004）	（647.55-7564.05）			（-0.0043—0.0271）
Azerbaijan	3385802.19（2936242.38-3858984.76）	30266.52	1.7%(1.1%—2.4%)	-0.0028（-0.0032—-0.0024）	7766.18（2160.09-26962.56）	67.04	-1.1%(-13.5%—12.6%)	-0.0111（-0.0198—-0.0024）
Georgia	1140978.63（1001451.59-1289365.37）	30274.88	0.8%(0.1%—1.5%)	-0.0023（-0.0025—-0.002）	2683.73（806.52-9086.85）	66.84	-6.8%(-17.9%—4.9%)	-0.0239（-0.0517—0.004）
Kazakhstan	5754105.77（4995971.47-6570472.25）	30289.04	2.1%(1.2%—2.8%)	-0.0013（-0.0015—-0.001）	12971.26（3567.84-44685.6）	67.00	1.4%(-10.0%—14.4%)	0.0046（-0.0042—0.0135）
Kyrgyzstan	2022325.21（1744686.36-2324835.63）	30280.71	1.4%(0.8%—2.0%)	-0.001（-0.0015—-0.0005）	4392.7（1146.89-15716.69）	67.08	3.8%(-8.0%—14.1%)	0.0072（-0.0032—0.0176）
Mongolia	983728.42（845953.31-1128245.12）	30281.81	2.4%(1.8%—3.0%)	0.0012*（0.0009—0.0015）	2168.19（587-7637.52）	66.78	0.1%(-10.8%—11.4%)	0.0198（0.0167—0.0229）
Tajikistan	2934517.29（2517479.42-3388486.22）	30255.25	1.4%(0.8%—2.0%)	-0.0024（-0.0028—-0.002）	6248.01（1599.96-22618.08）	66.73	0.3%(-10.7%—10.9%)	-0.0025（-0.0298—0.0248）
Turkmenistan	1563260.26（1344355.31-1790338.42）	30262.16	1.7%(1.0%—2.4%)	-0.0027（-0.0031—-0.0023）	3428.71（915.53-12240.14）	66.70	2.0%(-8.9%—16.5%)	-0.0003（-0.0038—0.0031）
Uzbekistan	10345179.75（8935412.33-11858961.37）	30269.39	1.5%(0.8%—2.2%)	-0.0016（-0.0019—-0.0013）	22906（6278.37-80976.37）	66.79	1.0%(-10.2%—10.5%)	0.0022（-0.0107—0.0151）
**Central Latin America**	**64199061.3（56253635.22-73519762.68）**	**24421.55**	**2.6%(2.2%—2.9%)**	**-0.0006（-0.0009—-0.0002）**	**141537.6（40933.41-477891.95）**	**53.44**	**1.8%(-3.7%—7.2%)**	**0.0175（0.012—0.023）**
Colombia	12356130.69（10673352.68-14396068.47）	23732.07	2.7%(1.9%—3.5%)	-0.0008（-0.0016—0）	27416.94（7966.17-92257.2）	51.70	3.8%(-7.8%—16.3%)	0.0272（0.0171—0.0373）
Costa Rica	1200333.35（1040235.79-1392871.87）	23742.75	1.8%(1.1%—2.6%)	0.0022（0.0018—0.0026）	2681.47（771.46-8939.74）	51.76	-4.4%(-13.5%—6.3%)	0.0278（0.0198—0.0358）
El Salvador	1549535.09（1323115.46-1822955.11）	23763.66	3.2%(2.5%—4.0%)	0.0017（0.0014—0.0021）	3328.99（915.97-11609.42）	51.86	7.8%(-3.2%—20.1%)	0.0405（0.0273—0.0536）
Guatemala	3738899.74（3163002.12-4421953.71）	23746.01	2.9%(2.2%—3.7%)	0.0007（0.0002—0.0013）	7724.34（2011.81-28453.06）	51.17	5.5%(-6.9%—19.7%)	0.0366（0.0226—0.0506）
Honduras	2377931.43（2002879.93-2815723.45）	23745.73	2.1%(1.3%—2.9%)	0.0012（0.0009—0.0014）	4897.45（1399.6-18208.7）	51.26	-3.8%(-14.8%—7.7%)	0.0135（-0.0012—0.0281）

Mexico	33820938.76（29932448.95-38345886.12）	25068.15	2.7%(2.2%—3.2%)	0（-0.0003—0.0002）	75259.03（22296.88-252604.32）	55.21	1.6%(-3.5%—7.1%)	0.0161（0.0087—0.0235）
Nicaragua	1597628.64（1357900.91-1889558.52）	23736.01	2.6%(1.8%—3.4%)	-0.002（-0.0021—-0.0018）	3367.83（907.42-12141.42）	51.30	3.0%(-8.2%—15.1%)	0.0213（0.0171—0.0256）
Panama	1037926.03（888007.44-1210721.91）	23715.99	2.2%(1.4%—3.0%)	0.0001（-0.0004—0.0007）	2252.29（618.15-7661.06）	51.32	-2.5%(-12.1%—8.5%)	0.0115（0.0032—0.0198）
Venezuela (Bolivarian Republic of)	6519737.56（5640313.77-7494465.54）	23756.85	2.2%(1.4%—3.0%)	-0.0041（-0.0044—-0.0038）	14609.26（4174.99-49321.47）	51.53	0.4%(-9.5%—9.4%)	0.0117（0.0032—0.0202）
**Andean Latin America**	**13910973.59（11804067.48-16155018.88）**	**20715.91**	**2.4%(-0.6%—5.5%)**	**0.0449（0.0355—0.0543）**	**32175.3（9600.31-117717.6）**	**48.09**	**1.9%(-7.7%—12.1%)**	**0.0341（0.022—0.0462）**
Bolivia (Plurinational State of)	2393952.24（2027937.59-2787789.95）	20238.68	1.2%(0.6%—1.8%)	0.0034（0.0027—0.0041）	5495.53（1676.43-20527.87）	47.30	0.0%(-11.5%—10.7%)	-0.011（-0.0279—0.0059）
Ecuador	3428315.91（2904183.22-4019443.8）	18708.72	8.6%(4.3%—13.6%)	0.2103（0.2019—0.2188）	8327.97（2680.54-30777.56）	45.90	1.3%(-12.2%—16.2%)	0.0964（0.0757—0.1171）
Peru	8088705.44（6856982.77-9417969.08）	21862.34	0.8%(-3.8%—5.5%)	0.0044（0.0023—0.0066）	18351.8（5377.78-66021.56）	49.43	3.2%(-9.5%—15.6%)	0.0225（0.0142—0.0308）
**Caribbean**	**11656582.16（10065531.46-13542557.84）**	**23730.24**	**1.1%(0.8%—1.4%)**	**-0.0007*（-0.0009—-0.0005）**	**25614.29（7423.31-86995.7）**	**51.21**	**-3.7%(-8.7%—1.6%)**	**0.0013（-0.0023—0.0048）**
Antigua and Barbuda	22930.44（19836.58-26458.33）	23728.21	1.1%(0.4%—1.8%)	-0.0034（-0.0038—-0.0029）	52.08（15.08-169.67）	51.47	-3.9%(-15.3%—6.4%)	-0.0105（-0.0207—-0.0004）
Bahamas	99724.39（86074.3-115670.06）	23748.98	0.8%(0.2%—1.4%)	-0.0004（-0.0009—0.0001）	222.89（64.31-731.54）	51.73	-4.4%(-15.8%—6.2%)	0.006（-0.0004—0.0124）
Barbados	77189.68（66749.24-88590.77）	23735.71	0.8%(0.3%—1.4%)	-0.0024（-0.0027—-0.0022）	180.17（54.27-568.13）	51.68	-5.1%(-15.4%—4.5%)	-0.0128（-0.0281—0.0024）
Belize	104325.73（88394.57-123407.78）	23720.48	1.5%(0.9%—2.3%)	0.0004（0—0.0008）	219.03（59.12-780.19）	51.26	-5.4%(-16.4%—5.1%)	0.0187（0.0152—0.0222）
Bermuda	16424.83（14181.68-18740.76）	23741.33	1.1%(0.5%—1.7%)	-0.001（-0.0014—-0.0007）	39.53（11.87-121.65）	51.87	-2.2%(-13.4%—10.3%)	0.0001（-0.012—0.0122）
Cuba	2888554.43（2486441.29-33	23717.59	2.2%(1.4%—3.0%)	0.0002（-0.0002—0	6759.85（1969.57-21769.	51.43	-0.3%(-11.2%—10.8%)	0.0148（0.0115—

	17748.89）			.0005）	7）			0.018）
Dominica	17093.29（14813.32-19854.52）	23714.53	1.0%(0.4%—1.5%)	-0.0043（-0.0046—-0.0041）	37.98（10.9-127.03）	51.14	-6.0%(-17.2%—4.6%)	-0.0288（-0.0326—-0.025）
Dominican Republic	2670258.88（2283640.8-3127352.94）	23723.71	1.9%(1.3%—2.5%)	-0.0021（-0.0025—-0.0017）	5740.95（1619.43-20061.2）	51.15	-3.6%(-14.0%—6.5%)	-0.0142（-0.0167—-0.0117）
Grenada	25879.71（22321.53-30085.85）	23710.78	1.4%(0.7%—2.0%)	-0.0048（-0.0052—-0.0043）	57.15（15.94-191.75）	50.96	-3.3%(-14.1%—8.2%)	-0.0212（-0.0282—-0.0142）
Guyana	183852.44（156891.87-215489.57）	23737.03	1.6%(1.0%—2.3%)	-0.0002（-0.0006—0.0003）	391.34（107.85-1357.45）	50.81	-3.6%(-13.7%—7.7%)	0.0141（0.0106—0.0175）
Haiti	2973615.72（2509332.6-3536365.34）	23736.29	0.7%(0.2%—1.3%)	-0.0023（-0.003—-0.0017）	6106.73（1742.54-22941.52）	50.76	1.6%(-8.1%—13.7%)	0.0178（0.0105—0.0251）
Jamaica	714407.4（614540.81-833780.47）	23722.86	1.0%(0.3%—1.6%)	-0.003（-0.0037—-0.0023）	1570.02（451-5337.95）	51.53	-3.4%(-13.8%—7.6%)	-0.005（-0.0307—0.0206）
Puerto Rico	860383.64（749129.12-984538.36）	23740.88	1.0%(0.2%—1.9%)	-0.0003（-0.0006—0.0001）	1998.37（584.08-6427.68）	51.78	-1.9%(-13.2%—9.1%)	0.0032（-0.0263—0.0327）
Saint Lucia	46001.09（39733.08-53005.55）	23718.12	1.3%(0.6%—2.0%)	-0.0051（-0.0056—-0.0045）	104.93（30.5-341.51）	51.11	-1.7%(-13.4%—9.7%)	-0.0102（-0.0262—0.0058）
Saint Vincent and the Grenadines	28536.94（24701.56-33014.01）	23704.30	1.9%(1.1%—2.6%)	-0.0047（-0.0052—-0.0042）	63.5（17.79-209.65）	50.94	-5.2%(-15.8%—6.0%)	-0.0114（-0.0151—-0.0078）
Suriname	142389.39（122948.94-165372.79）	23735.03	1.4%(0.8%—2.1%)	0.0029（0.0026—0.0032）	313.6（91.33-1057.01）	51.11	-7.6%(-17.2%—3.7%)	0.0058（-0.0024—0.0141）
Trinidad and Tobago	353485.07（305667.38-406113.47）	23723.31	1.4%(0.6%—2.2%)	-0.0007（-0.0011—-0.0004）	802.75（239.05-2633.91）	51.15	-5.2%(-14.8%—5.7%)	0.0009（-0.0049—0.0067）
United States Virgin Islands	21811.66（18843.13-24852.42）	23738.92	0.8%(0.2%—1.3%)	-0.0018（-0.0022—-0.0015）	51.6（15.39-160.72）	51.63	-6.2%(-17.4%—4.4%)	-0.0132（-0.0263—-0.0001）
Saint Kitts and Nevis	15245.88 (13106.12-17573.99)	23738.23	-0.04%（-0.48%—0.35%）	-0.0012（-0.0016—-0.0009）	34.99 (10.23-112.90)	51.214085177	-0.02%（-9.22%—12.21%）	0.0033（-0.0001—0.0067）
**Tropical Latin America**	**68927383.26（61158241.36-77275223.47）**	**29005.61**	**1.6%(-0.6%—3.9%)**	**0.0148（-0.043—0.0726）**	**137481.22（36765.57-495028.56）**	**55.97**	**0.8%(-5.3%—6.3%)**	**0.0258（-0.0016—0.0533）**
Brazil	66988588.42	29095.41	1.7%(-0.6%—4.1%)	0.0178	133775.26	56.12	0.9%(-5.1%—6.4%)	0.028

	(59513587.49-75079315.71)			(-0.0418—0.0775)	(35756.65-480933.9)			(0—0.0561)
Paraguay	1938794.85 (1642790.59-2260984.77)	26396.49	1.2%(0.6%—1.9%)	-0.0007 (-0.0012—-0.0003)	3705.95 (962.01-13884.76)	51.27	-4.2%(-17.3%—10.0%)	0.014 (0.0108—0.0173)
**East Asia**	**293499971.57 (259912831.84-331480658.61)**	**18489.51**	**11.9%(7.9%—16.2%)**	**0.2424 (0.1758—0.3091)**	**740187.45 (232174.55-2249350.4)**	**43.40**	**9.5%(1.7%—24.6%)**	**0.1473 (0.1347—0.1598)**
China	283814151.04 (251438661.81-320431556.78)	18525.07	12.3%(8.1%—16.8%)	0.2516 (0.1822—0.321)	716164.94 (224403.14-2174717.29)	43.50	9.8%(1.8%—25.4%)	0.1515 (0.1388—0.1642)
Democratic People's Republic of Korea	5018898.89 (4305080.02-5845854.19)	17560.91	2.5%(1.5%—3.4%)	-0.0253 (-0.0259—-0.0247)	12198.72 (3565.2-39609.21)	40.51	3.3%(-7.1%—14.8%)	-0.0119 (-0.0157—-0.0081)
Taiwan	4666921.63 (4033587-5379560.7)	17557.81	1.7%(0.5%—2.5%)	0.013 (0.0126—0.0133)	11823.79 (3743.6-35485.04)	41.13	-0.6%(-12.3%—12.8%)	0.0698 (0.0532—0.0865)
**Southeast Asia**	**183412192.54 (159923055.59-207822999.95)**	**25256.68**	**3.0%(2.5%—3.5%)**	**0.0024 (0.0022—0.0026)**	**377303.17 (103758.82-1323330.62)**	**51.03**	**6.0%(0.0%—12.6%)**	**0.0265 (0.0203—0.0326)**
Cambodia	4188882.33 (3556166.6-4849992.68)	24628.97	1.9%(1.3%—2.7%)	-0.0098 (-0.0101—-0.0095)	8215.77 (2132.03-30950.54)	48.94	13.9%(2.0%—28.6%)	0.0166 (-0.0023—0.0356)
Indonesia	75310226.24 (66121275.8-84705049.26)	25774.93	3.0%(2.5%—3.5%)	-0.004 (-0.0044—-0.0036)	156759.37 (43311.15-541443.71)	52.56	5.3%(-1.3%—12.5%)	0.0177 (0.0112—0.0241)
Lao People's Democratic Republic	1786179.24 (1505804.55-2076696.77)	24566.31	2.4%(1.7%—3.0%)	-0.0063 (-0.0068—-0.0058)	3487.72 (915.73-13089.58)	48.96	10.7%(-2.8%—23.9%)	0.019 (0.0072—0.0308)
Malaysia	8207794.25 (6982466.09-9462340.08)	24519.06	3.3%(-0.9%—7.5%)	-0.0051 (-0.0061—-0.0041)	16492.33 (4278.76-59105.84)	48.99	1.0%(-10.5%—14.1%)	0.011 (-0.0107—0.0327)
Maldives	138987.07 (116424.37-162994.31)	24278.21	2.7%(1.8%—3.6%)	-0.027 (-0.0282—-0.0258)	280.77 (75.15-1046.43)	48.16	12.4%(-1.0%—25.4%)	-0.0058 (-0.0139—0.0023)
Mauritius	344114.42 (298128.43-392826.48)	24573.74	2.1%(1.2%—3.0%)	-0.0013 (-0.002—-0.0006)	719.49 (202.68-2446.86)	48.84	-1.8%(-12.8%—9.3%)	0.0013 (-0.0093—0.0119)
Myanmar	14083390.84 (12001829.73-16114929.58)	24636.82	2.5%(1.9%—3.1%)	0.0045 (0.0043—0.0047)	28147.31 (7370.18-103171.99)	49.08	8.2%(-4.0%—22.4%)	0.0458 (0.0237—0.0679)
Philippines	29182745.6 (25474185.57-32906718.16)	25783.43	2.4%(2.0%—2.8%)	-0.001 (-0.0012—-0.0008)	58363.9 (15786.76-207311.36)	52.38	4.2%(-0.4%—9.0%)	0.017 (0.0137—0.0204)

Sri Lanka	5772366.93（4994847-6593052.92）	24617.15	3.1%(2.4%—3.8%)	0.0079（0.0076—0.0083）	11817.22（3197.16-42494.12）	49.12	1.4%(-11.0%—13.6%)	0.0268（0.0199—0.0336）
Seychelles	27194.46（23462.59-31089.22）	24479.93	2.4%(1.7%—3.1%)	-0.0118（-0.0124—-0.0113）	56.6（15.44-195.29）	48.90	-5.9%(-18.4%—6.3%)	-0.0219（-0.0293—-0.0145）
Thailand	18206201.03（15772052.52-20749332.89）	24604.29	3.4%(2.7%—4.1%)	0.0014（0.0008—0.002）	39021.68（11121.81-129338.28）	49.25	6.9%(-3.4%—20.8%)	0.027（-0.0023—0.0563）
Timor-Leste	319576.82（267872.47-374377.82）	24554.54	2.2%(1.5%—2.9%)	0.0041（0.0036—0.0045）	599.7（146.64-2311.03）	48.69	10.1%(-2.1%—24.2%)	0.0343（0.0083—0.0604）
Viet Nam	25588712.12（21974227.6-29356400.06）	24581.87	3.1%(2.4%—3.8%)	-0.0101（-0.0104—-0.0099）	52815.04（14438.94-189611.67）	49.38	8.1%(-4.2%—20.2%)	0.0186（0.0132—0.0239）
**Oceania**	**2877452.51（2425463.29-3386549.14）**	**22142.50**	**1.1%(0.6%—1.5%)**	**0.0011（0.0007—0.0015）**	**5814.79（1591.93-21508.33）**	**46.21**	**1.8%(-7.9%—10.6%)**	**0.0112（0.0086—0.0139）**
American Samoa	11189.66（9641.02-12900.55）	22152.00	0.5%(-0.3%—1.2%)	0.0012（0.0007—0.0017）	23.56（6.4-83.04）	46.26	-8.0%(-18.1%—2.0%)	-0.0166（-0.0471—0.0139）
Micronesia (Federated States of)	22753.48（19356.94-26612.16）	22170.51	1.3%(0.7%—1.9%)	0.0021（0.0014—0.0028）	47.11（13.01-168.68）	46.47	1.0%(-10.6%—14.8%)	0.0122（0.0043—0.0201）
Fiji	204510.42（175138.47-236948.64）	22173.81	1.1%(0.4%—1.8%)	0.0009（0.0006—0.0011）	430.22（119.91-1523.95）	46.41	-2.9%(-13.1%—7.7%)	-0.003（-0.0166—0.0106）
Guam	36712.14（31712.67-41861.98）	22159.46	0.8%(0.1%—1.5%)	0.0094（0.0087—0.01）	80.09（22.8-269.33）	46.94	-5.6%(-15.4%—5.8%)	0.0205（0.0128—0.0282）
Kiribati	25822.87（21861.72-30240.84）	22252.77	1.2%(0.7%—1.9%)	0.0038（0.0034—0.0042）	52.43（14.18-191.37）	46.50	6.1%(-6.0%—19.6%)	0.0212（0.0155—0.0269）
Marshall Islands	12402.18（10513.89-14473.24）	22147.43	1.2%(0.7%—1.8%)	-0.0003（-0.0005—-0.0001）	25.47（7.24-91.93）	46.15	-5.3%(-16.7%—6.0%)	-0.0049（-0.0241—0.0142）
Northern Mariana Islands	11246.56（9685.9-12901.62）	22111.12	0.6%(-0.1%—1.2%)	0.0094（0.0008—0.018）	24.89（7.09-84.14）	46.70	-4.3%(-13.5%—6.9%)	0.0166（-0.022—0.0552）
Papua New Guinea	2139597.99（1798969.39-2524412.06）	22129.08	1.3%(0.7%—1.8%)	0.0002（-0.0004—0.0007）	4296.59（1168.09-16055.7）	46.12	4.4%(-9.0%—17.7%)	0.0172（0.0137—0.0207）
Samoa	43641.39（36950.91-5075	22147.15	1.1%(0.5%—1.8%)	0.0012（0.001—0.0	88.46（22.77-324.45）	46.47	-1.9%(-14.1%—9.5%)	0.0005（-0.003—0

	4.58）			014）				.0041）
Solomon Islands	139901.61（117340.63-164312.28）	22166.62	1.3%(0.8%—1.9%)	0.0088（0.0083—0.0092）	280.04（78.1-1060.98）	46.35	-0.1%(-10.9%—13.9%)	0.0245（0.0185—0.0306）
Tonga	21838.8（18532.45-25315.85）	22214.78	1.3%(0.7%—2.0%)	0.0016（0.0012—0.0021）	44.61（12.11-162.63）	46.81	-4.6%(-15.6%—8.3%)	-0.0013（-0.0079—0.0053）
Vanuatu	64424.73（54313.48-75428.07）	22181.20	1.3%(0.7%—1.9%)	0.008（0.0075—0.0085）	129.67（34.68-481.53）	46.41	0.3%(-11.4%—13.2%)	0.0197（0.0017—0.0377）
Cook Islands	4165.80（3595.12-4739.29）	22226.16	0.48%（0.02%—0.95%）	0.0156（0.0145—0.0168）	9.90（2.71-30.64）	46.86	0.46%(-10.33%—10.94%)	0.0122（-0.0153—0.0397）
Nauru	2315.70（1947.16-2729.54）	22210.98	0.53%（0.04%—1.08%）	0.0170（0.0167—0.0173）	4.64（1.23-17.55）	46.50	0.59%（-8.82%—12.32%）	0.024（0.0202—0.0277）
Niue	386.42（334.25，440.97）	22182.00	-0.02%（-0.47%—0.4%）	-0.0006（-0.0011—-0.0001）	0.84（0.25，2.86）	46.66	0.09%(-10.18%—12.65%)	-0.0038（-0.0074—-0.0003）
Palau	4331.42（3720.52-4942.71）	22029.78	-0.53%（-1.08%—0.06%）	-0.0173（-0.0208—-0.0139）	9.74（2.81-32.72）	46.09	-1.01%（-12.37%—10.22%）	-0.0339（-0.0578—-0.0099）
Tokelau	309.35（266.03-355.44）	22166.23	-0.22%（-0.62%—0.24%）	-0.0068（-0.0095—-0.004）	0.65（0.18-2.30）	46.52	-0.18%（-10.97%—10.39%）	0.0009（-0.0253—0.0272）
Tuvalu	2712.46（2318.80-3154.33）	22139.86	-0.67%(-1.18%—-0.19%)	-0.0217（-0.0223—-0.021）	5.62（1.58，19.89）	46.46	-0.93%（-11.19%—8.89%）	-0.0292（-0.0529—-0.0056）
**North Africa and Middle East**	**150059430.59（129299778.38-172131899.92）**	**24090.29**	**2.4%(1.1%—3.7%)**	**0.0307（0.0226—0.0389）**	**424576.65（139454.67-1253875.68）**	**67.78**	**-1.5%(-10.6%—5.4%)**	**0.0104（0.002—0.0189）**
Afghanistan	6238846.12（5140825-7393954.11）	23578.38	2.1%(1.3%—2.8%)	0.0131（0.0116—0.0146）	16062.91（4933.36-50943.69）	65.57	0.4%(-12.6%—19.3%)	-0.0166（-0.0284—-0.0048）
Algeria	10273555.77（8781010.85-11980460.4）	23517.68	1.6%(0.7%—2.5%)	0.0022（-0.0026—-0.0017）	29488.28（9845.79-86751.09）	66.65	0.3%(-9.6%—10.1%)	0.0046（-0.0056—0.0147）
Bahrain	391731.76（329947.42-458797.69）	23559.15	0.8%(0.2%—1.5%)	-0.0055（-0.0083—-0.0027）	1125.17（367.09-3364.26）	64.36	-4.1%(-14.7%—5.1%)	-0.0328（-0.0499—-0.0157）
Egypt	25795119.63（21998153.51-29905928.26）	25845.30	5.9%(0.5%—12.5%)	0.0998（0.0951—0.1044）	67961.5（20728.58-212870.2）	68.90	0.5%(-11.6%—11.5%)	0.0502（0.0379—0.0625）
Iran (Islamic	24471146.9	27244.00	1.8%(0.0%—3.5%)	0.0951	69616.55	74.74	0.6%(-9.7%—7.4%)	0.0457

Republic of)	(21659838.49-27457285.61)			(-0.0046—0.1949)	(22337.78-206301.03)			(0.0334—0.058)
Iraq	9487509.36 (8005870.27-11070066.97)	23545.34	1.5%(0.7%—2.3%)	0.0007 (0—0.0015)	26056.17 (8444.13-79837.66)	65.89	-1.1%(-13.1%—9.2%)	0.0044 (-0.0051—0.0139)
Jordan	2924668.24 (2481117.99-3411638.67)	23532.88	1.2%(0.4%—1.9%)	0.0027 (0.0023—0.0031)	8117.34 (2569.63-24852.19)	65.89	-3.8%(-14.1%—5.4%)	-0.0169 (-0.032—-0.0018)
Kuwait	1202810.88 (1013103.88-1415709.23)	23548.04	-0.3%(-3.9%—3.6%)	-0.0295 (-0.0356—-0.0233)	3460.02 (1123.07-10207.24)	63.16	-6.4%(-17.8%—4.6%)	0.0393 (0.0252—0.0533)
Lebanon	1377054.96 (1181449.04-1599794.73)	23534.28	1.7%(0.7%—2.6%)	0.0027 (0.0019—0.0035)	3959.25 (1303.08-11664.51)	66.55	-3.6%(-15.1%—7.6%)	0.0001 (-0.0362—0.0363)
Libya	1732718.38 (1473084.52-2020501.58)	23542.45	0.6%(-0.2%—1.3%)	-0.0012 (-0.0021—-0.0002)	5038.78 (1696.03-14678.28)	66.22	-9.0%(-19.6%—0.5%)	0.0188 (-0.0061—0.0437)
Morocco	8952877.69 (7725830.82-10404956.58)	23523.54	2.1%(1.2%—3.0%)	0.0002 (-0.0003—0.0007)	25583.89 (8438.52-74630.66)	66.29	-4.9%(-15.2%—4.5%)	0.0024 (-0.0053—0.01)
Oman	1148073.56 (951232.46-1353429.71)	23580.68	1.5%(0.8%—2.2%)	-0.0012 (-0.0021—-0.0004)	3240.81 (1053.23-9699.15)	64.81	0.9%(-12.2%—11.6%)	0.0184 (0.0007—0.0361)
Palestine	1142578.51 (959157.69-1352235.83)	23528.29	1.9%(1.0%—2.7%)	-0.0011 (-0.0019—-0.0004)	3090.36 (954.02-9464.47)	66.30	-4.6%(-16.4%—5.7%)	-0.016 (-0.0264—-0.0056)
Qatar	785757.44 (638136.01-935428.85)	23526.77	0.7%(0.1%—1.4%)	0.0004 (-0.0013—0.0022)	2248.08 (741.11-6479.06)	63.20	-2.9%(-13.9%—6.8%)	0.0018 (-0.0172—0.0208)
Saudi Arabia	9198589.78 (7776891.23-10733004.61)	22434.33	1.9%(1.3%—2.6%)	-0.0189 (-0.0289—-0.0089)	27608.02 (8989.43-79078.05)	63.98	0.9%(-11.4%—10.5%)	-0.0049 (-0.0121—0.0022)
Sudan	9474023.54 (7936915.25-11198665.23)	23470.17	1.8%(1.1%—2.5%)	-0.0048 (-0.0053—-0.0043)	25471.44 (7888.28-78904.39)	66.39	3.4%(-9.1%—14.4%)	0.017 (0.0096—0.0244)
Syrian Arab Republic	3423414.55 (2954160.55-3950273.21)	23519.33	0.6%(-0.2%—1.4%)	0.0008 (-0.0006—0.0021)	9772.35 (3176.4-28560.94)	67.11	-7.2%(-18.3%—1.8%)	0.0436 (0.0247—0.0626)
Tunisia	2920816.05 (2526502.76-3371004.16)	23526.35	1.2%(0.7%—1.8%)	0.0022 (0.0018—0.0025)	8537.4 (2768.57-24868.56)	66.63	-8.4%(-19.7%—0.4%)	0.0023 (-0.0083—0.0128)
Türkiye	19195886.34 (16675901.77-22120726.69)	21659.04	3.4%(-0.6%—7.4%)	0.0298 (-0.0091—0.0687)	61026.19 (20824.09-162820.08)	66.80	-0.4%(-11.1%—9.8%)	0.0194 (-0.0166—0.0553)

United Arab Emirates	2594811.47（2110326.46-3103190.43）	23538.75	0.8%(0.0%—1.6%)	-0.0083（-0.0096—-0.007）	7728.79（2460.67-22149.12）	63.46	-1.6%(-13.1%—9.9%)	-0.0025（-0.0201—0.015）
Yemen	7187478.55（6037041.17-8443389.05）	23532.90	1.3%(0.7%—1.8%)	-0.0009（-0.0014—-0.0003）	18987.33（5828.37-59468.52）	66.08	2.1%(-10.2%—12.0%)	0.0265（0.017—0.036）
**South Asia**	**483244864.79（423704615.12-550135164.09）**	**25405.47**	**1.8%(1.5%—2.2%)**	**0（-0.0087—0.0088）**	**954549.29（250693.32-3465828.86）**	**50.98**	**5.9%(-1.0%—13.8%)**	**0.0118（0.001—0.0226）**
Bangladesh	41769701.85（35462421.73-48926057.12）	24771.44	2.2%(1.5%—2.9%)	-0.0049（-0.0054—-0.0045）	81382.88（21253.16-297617.76）	48.87	8.7%(-3.0%—21.9%)	0.0663（0.0416—0.091）
Bhutan	197371.55（167533.67-232593.86）	24804.70	2.8%(1.8%—3.7%)	0.0002（0—0.0004）	381.98（98.96-1434.02）	48.58	6.9%(-4.7%—24.6%)	0.0321（0.0285—0.0356）
India	373405604.67（329155714.75-422556131.86）	25298.04	1.5%(1.2%—1.8%)	-0.0028（-0.0152—0.0095）	758396.08（204152.45-2689270.98）	51.88	7.7%(0.4%—15.1%)	0.0421（0.0311—0.053）
Nepal	7729040.28（6506174.31-9165779.44）	24613.27	1.9%(-2.3%—6.1%)	-0.0345（-0.0366—-0.0323）	15473.53（3992.24-57363.77）	50.69	8.1%(-6.0%—23.5%)	0.0333（0.0063—0.0604）
Pakistan	60143146.44（52453133.63-68878998.64）	26707.03	2.4%(1.8%—3.0%)	-0.0066（-0.0135—0.0004）	98914.82（21954.54-418386.26）	46.10	-8.8%(-25.2%—3.4%)	-0.2528（-0.2671—-0.2384）
**Southern Sub-Saharan Africa**	**19579978.23（17105205.49-22446316.6）**	**24193.08**	**1.9%(1.5%—2.3%)**	**-0.0012（-0.0015—-0.0009）**	**45063.53（13615.37-144888.95）**	**57.04**	**-9.3%(-14.9%—-4.8%)**	**-0.0239（-0.0316—-0.0161）**
Botswana	568777.6（486942.05-669055.22）	23234.87	2.1%(1.4%—2.8%)	-0.002（-0.0022—-0.0017）	1289.1（380.01-4263.34）	54.15	-7.7%(-18.3%—1.6%)	-0.0247（-0.0342—-0.0153）
Lesotho	425794.65（361236.49-503366.56）	23245.90	2.4%(1.6%—3.2%)	-0.0043（-0.0047—-0.0038）	929.29（261.38-3157.46）	53.73	-18.9%(-27.8%—-10.7%)	-0.0828（-0.0928—-0.0727）
Namibia	550115.32（467216.43-649779.3）	23242.32	2.3%(1.5%—3.0%)	0.0005（0.0004—0.0006）	1226.78（339.68-4156.51）	54.53	-4.1%(-15.5%—6.2%)	0.0041（-0.016—0.0242）
South Africa	14475342.22（12693192.54-16417847.52）	24528.71	1.8%(1.4%—2.2%)	-0.0012（-0.0014—-0.001）	33890.82（10290.21-106850.22）	57.93	-10.0%(-15.3%—-5.5%)	-0.0311（-0.0394—-0.0227）
Eswatini	257677.38（217806.96-305251.57）	23237.60	2.2%(1.5%—2.9%)	-0.0017（-0.0023—-0.0011）	561.45（163.09-1909.44）	54.04	-17.5%(-26.3%—-8.6%)	-0.0496（-0.0539—-0.0454）
Zimbabwe	3302271.06（2790081.26-39	23244.83	2.1%(1.4%—2.8%)	0.0012（0.0009—0.	7166.09（2047.06-24476.	54.40	-3.9%(-14.9%—7.4%)	0.0042（-0.0107—

	09670.06）			0014）	62）			0.019）
**Western Sub-Saharan Africa**	**105712751.72（90445006.66-122433717.63）**	**25108.38**	**1.1%(0.9%—1.4%)**	**-0.0004（-0.0041—0.0034）**	**222777.31（62638.96-792248.22）**	**57.49**	**8.6%(2.5%—14.7%)**	**0.0492（0.0433—0.0551）**
Benin	2800066.95（2364067.79-3304937.3）	24550.25	1.0%(0.5%—1.6%)	-0.001（-0.0014—-0.0007）	5808.13（1636.42-21576.55）	55.55	10.2%(-0.5%—22.4%)	0.0158（0.0122—0.0193）
Burkina Faso	4676265.49（3942505.24-5512968.81）	24561.03	1.1%(0.6%—1.6%)	-0.0002（-0.0006—0.0002）	9756.89（2684.41-36310.27）	55.77	13.7%(-1.0%—27.3%)	0.0315（0.0154—0.0475）
Cameroon	6862598.47（5788689.1-8076567.17）	24535.48	1.8%(1.2%—2.4%)	-0.0015（-0.0017—-0.0012）	14319.28（3780.44-51048.74）	55.45	7.3%(-4.9%—19.9%)	0.0221（0.0118—0.0324）
Cabo Verde	143866.17（123573.24-167969.9）	24530.58	2.7%(1.9%—3.6%)	-0.0069（-0.0072—-0.0067）	322.01（90.97-1075.87）	55.63	-2.0%(-12.5%—8.3%)	-0.0375（-0.0413—-0.0337）
Chad	3394718.73（2845497.74-4024876.01）	24531.89	0.6%(0.1%—1.2%)	-0.0039（-0.004—-0.0037）	6857.96（1809.15-26176.12）	54.94	3.3%(-10.0%—15.6%)	-0.0109（-0.0183—-0.0036）
Côte d'Ivoire	6056057.26（5105231.41-7100109.44）	24501.69	1.5%(0.9%—2.2%)	-0.0011（-0.0014—-0.0008）	12696.17（3495.16-45978.41）	55.02	16.3%(3.5%—30.3%)	0.0413（0.0376—0.0449）
Gambia	522253.47（440704.44-615055.43）	24545.08	1.6%(1.0%—2.2%)	0.0026（0.0022—0.003）	1084.33（294.65-3935.24）	55.39	-2.1%(-14.6%—9.5%)	0.0272（0.019—0.0354）
Ghana	7796168.88（6602979.81-9150195.06）	24555.80	1.7%(1.1%—2.2%)	0.0022（0.0015—0.0029）	16762.55（4810.37-58400.17）	55.95	6.3%(-5.1%—18.8%)	0.0331（0.0189—0.0473）
Guinea	2796790.24（2364581.85-3296479.78）	24553.71	0.9%(0.4%—1.5%)	0.0003（0—0.0006）	5810.42（1530.14-21242.87）	55.55	2.6%(-8.8%—14.1%)	0.0149（-0.0088—0.0386）
Guinea-Bissau	440352.48（370231.14-518968.15）	24557.15	1.3%(0.8%—1.8%)	-0.0003（-0.0008—0.0001）	913.12（254.59-3394.69）	55.56	6.5%(-3.9%—18.1%)	0.0205（0.0161—0.0249）
Liberia	1219320.58（1026314.34-1429511.25）	24518.93	0.8%(0.3%—1.4%)	-0.0015（-0.0037—0.0007）	2521.19（697.04-9253.55）	54.33	8.4%(-2.4%—20.8%)	0.0238（0.0059—0.0417）
Mali	4782271.65（4023725.67-5650515.2）	24533.94	0.7%(0.3%—1.1%)	-0.0015（-0.002—-0.001）	9772.23（2656.87-36313.63）	55.09	3.5%(-7.4%—13.9%)	0.0119（0.0019—0.0219）
Mauritania	946874.5（802649.4-1112625.99）	24545.61	1.7%(1.2%—2.3%)	0.0002（-0.0001—0.0004）	2007.6（561.32-7049.02）	55.94	5.9%(-5.1%—18.3%)	0.019（-0.0098—0.0478）
Niger	4767964.3	24545.47	0.5%(0.1%—1.1%)	0.0007	9652.65	55.43	8.6%(-1.6%—22.1%)	0.0372

	（3990759.46-5671222.98）			（0.0002—0.0013）	（2633.05-38579.54）			（0.0284—0.046）
Nigeria	51144630.66（44479733.4-58495139.86）	25749.89	1.2%(0.8%—1.7%)	0.0043（-0.0019—0.0105）	108948.73（30762.2-380646.5）	59.81	10.0%(2.2%—17.3%)	0.0846（0.0772—0.0921）
Sao Tome and Principe	50554.86（42999.04-59221.17）	24532.89	1.9%(1.2%—2.5%)	-0.0018（-0.0022—-0.0014）	108.9（30.78-373.73）	55.73	1.1%(-9.6%—11.6%)	-0.0039（-0.007—-0.0009）
Senegal	3494948.03（2962793.15-4108860.63）	24528.55	1.3%(0.9%—1.8%)	-0.0031（-0.0034—-0.0029）	7365.08（1983.62-26606.43）	55.39	4.4%(-7.2%—15.9%)	0.0231（0.0191—0.0271）
Sierra Leone	1948920.18（1646341.3-2300172.14）	24535.33	1.1%(0.4%—1.6%)	-0.0004（-0.0011—0.0002）	4071.31（1151.31-14863.55）	55.22	7.2%(-3.0%—17.7%)	0.0141（0.0105—0.0177）
Togo	1866895.38（1580732.12-2188166.84）	24549.89	1.3%(0.8%—1.8%)	-0.0006（-0.0009—-0.0002）	3996.18（1141.35-14295.86）	55.90	5.8%(-6.1%—17.7%)	0.0195（-0.003—0.0419）
**Eastern Sub-Saharan Africa**	**72013735.88（60934221.55-85227433.29）**	**18801.27**	**-0.6%(-1.4%—0.3%)**	**-0.0631（-0.0662—-0.06）**	**166767.07（50404.43-560675.01）**	**48.12**	**6.4%(0.8%—12.7%)**	**-0.0297（-0.0408—-0.0187）**
Burundi	2254001.22（1892014.97-2688235.84）	19503.76	1.3%(0.8%—1.9%)	-0.0001（-0.0003—0.0001）	5144.82（1492.1-18101.64）	49.23	4.0%(-6.1%—16.4%)	-0.0299（-0.0358—-0.0241）
Comoros	142901.37（121912.82-168215.83）	19504.26	2.0%(1.3%—2.8%)	0.0012（0.0009—0.0016）	351.5（109.37-1113.03）	49.80	3.7%(-7.8%—17.0%)	0.003（-0.0217—0.0276）
Djibouti	242163.34（206120.56-285662.79）	19489.09	1.9%(1.2%—2.7%)	-0.0019（-0.0022—-0.0016）	586.4（177.71-1918.38）	49.20	0.3%(-10.0%—12.2%)	0.0038（-0.0072—0.0149）
Eritrea	1198900.46（1011128.59-1419206.29）	19509.71	1.8%(1.2%—2.4%)	0.0016（0.0012—0.002）	2825.94（861.06-9565.08）	49.61	15.2%(3.2%—27.7%)	0.0201（0.0159—0.0243）
Ethiopia	15804443.25（13305706.99-18652059.48）	15855.33	-7.7%(-10.9%—-4.3%)	-0.2982（-0.3067—-0.2896）	36133.82（10976.55-124315.72）	40.27	0.3%(-8.1%—9.4%)	-0.2764（-0.2919—-0.261）
Kenya	9877023.1（8572582.74-11302288.33）	20758.45	1.4%(1.1%—1.7%)	-0.0002（-0.0003—0）	23442.21（7144.43-74981.17）	53.60	1.4%(-4.5%—7.3%)	0.0145（0.007—0.0219）
Madagascar	5046562.2（4239617.73-6029981.4）	19501.66	1.7%(1.2%—2.1%)	0.0003（-0.0001—0.0006）	11815.93（3452.46-39780.29）	49.90	8.0%(-2.4%—20.4%)	0.0388（0.0227—0.0548）
Malawi	3446062.62（2886804.59-4116394.32）	19504.05	1.6%(1.0%—2.3%)	0（-0.0002—0.0002）	7888.77（2313.22-26783.85）	49.79	11.9%(-0.9%—25.6%)	0.0394（0.0281—0.0507）

Mozambique	5188149.73（4347386.27-6212738.49）	19504.07	1.3%(0.8%—1.8%)	0.0011（0.0008—0.0014）	11703.7（3525.76-40138.83）	49.23	1.0%(-10.9%—12.4%)	0.0026（-0.0234—0.0287）
Rwanda	2430652.18（2051533.09-2885789.17）	19506.59	2.0%(1.5%—2.7%)	-0.0001（-0.0007—0.0005）	5799.35（1744.49-19400.17）	50.12	5.2%(-4.8%—18.9%)	0.0205（0.0063—0.0347）
Somalia	3505207.29（2938257.15-4166552.66）	19507.50	0.2%(-0.2%—0.6%)	0.002（0.0017—0.0022）	7813.46（2408.69-29169.75）	49.29	1.1%(-8.0%—13.5%)	0.0188（0.0035—0.034）
South Sudan	1644370.56（1387374.53-1952436.51）	19484.86	0.7%(0.2%—1.4%)	-0.0009（-0.0012—-0.0007）	3795.43（1125.47-13115.17）	49.21	3.3%(-5.7%—15.5%)	0.0593（0.0526—0.0659）
United Republic of Tanzania	10210203.09（8620784.36-12145846.14）	19499.54	1.8%(1.3%—2.3%)	-0.0001（-0.0002—0.0001）	23874.63（6965.88-79104.74）	49.85	15.8%(3.2%—28.8%)	0.0478（0.0236—0.072）
Uganda	7243366.49（6048172.16-8627045.41）	19499.97	1.5%(1.0%—2.0%)	-0.0004（-0.0008—0）	16438.77（4936.75-56222.79）	49.87	14.9%(1.5%—30.1%)	0.0622（0.0427—0.0817）
Zambia	3717034.57（3078176.76-4440450.44）	21241.53	1.3%(0.8%—1.8%)	-0.02（-0.0325—-0.0074）	9007.17（2762.36-29349.93）	57.44	4.9%(-6.2%—19.2%)	0.0577（0.0165—0.0988）
**Central Sub-Saharan Africa**	**28081944.01（23624227.76-33293382.86）**	**23232.00**	**0.8%(0.5%—1.2%)**	**-0.0004（-0.0008—0）**	**60000.45（16931.68-208886.1）**	**53.98**	**10.6%(1.7%—20.8%)**	**0.0286（0.0212—0.0361）**
Angola	6412912.2（5383565.47-7594832.51）	23244.25	1.4%(0.8%—2.0%)	0.0027（0.0023—0.0032）	13649.23（3799.95-47603.44）	54.40	4.0%(-8.5%—17.2%)	0.0536（0.0338—0.0734）
Central African Republic	1137707.16（957908.89-1349721.72）	23237.18	0.4%(0.0%—0.9%)	-0.0003（-0.0005—-0.0001）	2446.33（697-8587.6）	53.86	7.1%(-3.0%—18.6%)	0.0313（0.0243—0.0382）
Congo	1202872.73（1021112.69-1417173.79）	23228.55	0.8%(0.3%—1.3%)	-0.0017（-0.0021—-0.0013）	2666.69（773.03-9168.65）	54.11	1.5%(-9.1%—12.2%)	-0.0055（-0.0123—0.0013）
Democratic Republic of the Congo	18594997.52（15632801.39-22060421.47）	23228.07	0.6%(0.2%—1.1%)	-0.0011（-0.0013—-0.0008）	39640.45（11287.13-140089.88）	53.83	13.1%(0.9%—26.4%)	0.0233（0.0136—0.0331）
Equatorial Guinea	329721.99（275516.7-394425.48）	23202.58	2.2%(1.3%—3.0%)	-0.0064（-0.0067—-0.0061）	694.09（192.1-2446.55）	54.15	12.9%(-3.5%—29.2%)	0.0254（0.0177—0.0331）
Gabon	403732.42（343107.65-476119.9）	23246.38	1.3%(0.6%—2.0%)	0.0012（0.0006—0.0018）	903.66（266.03-3000.04）	54.44	1.5%(-9.9%—13.6%)	0.0256（0.0181—0.0332）

Supplementary Figure 1. Percentage Change in Age-Standardized YLDs and Prevalence Rates for Migraine by Region（1990-2021）

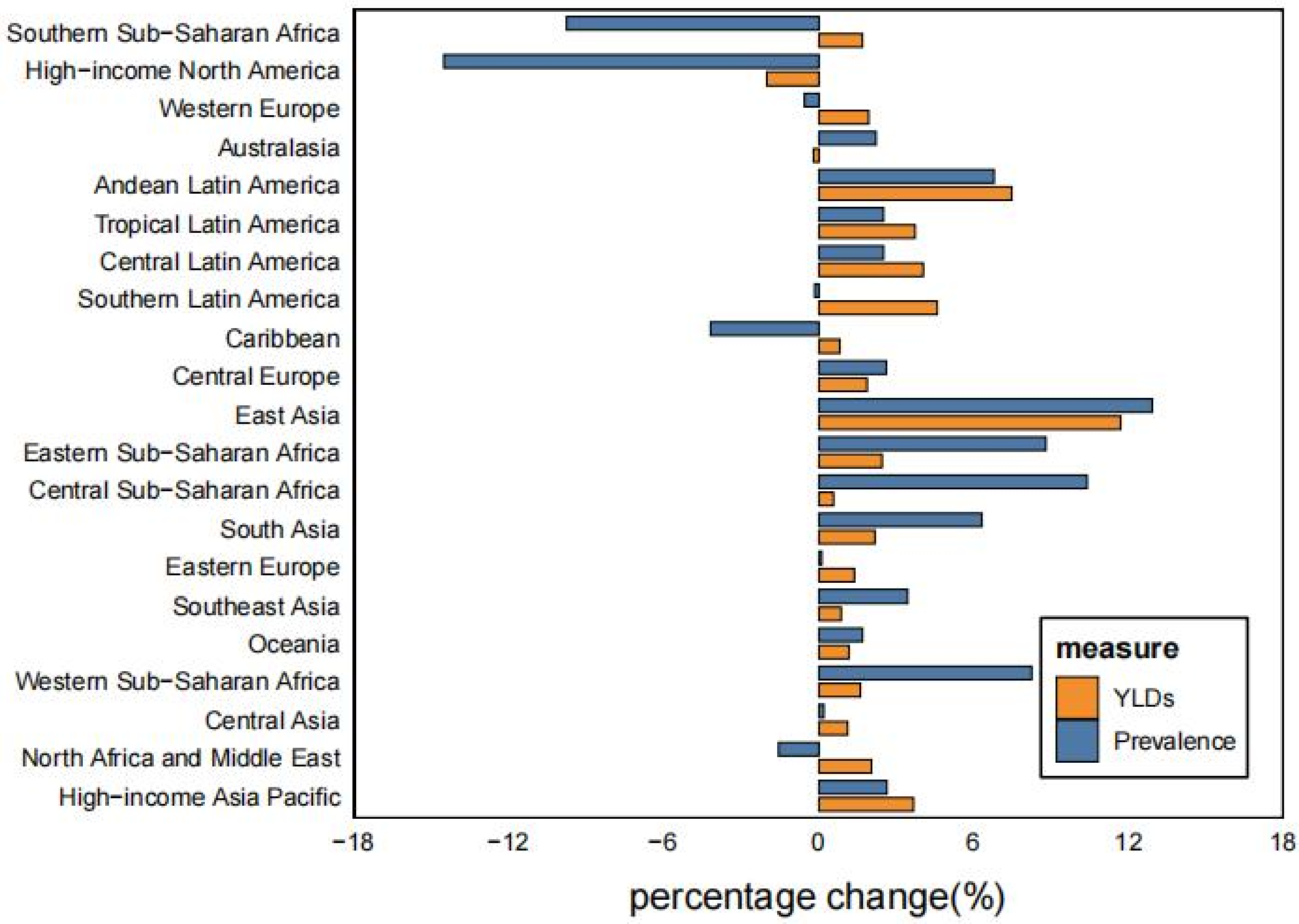

Supplementary Figure 2. Percentage Change in Age-Standardized YLDs and Prevalence Rates for Migraine by SDI quintiles level（1990-2021）

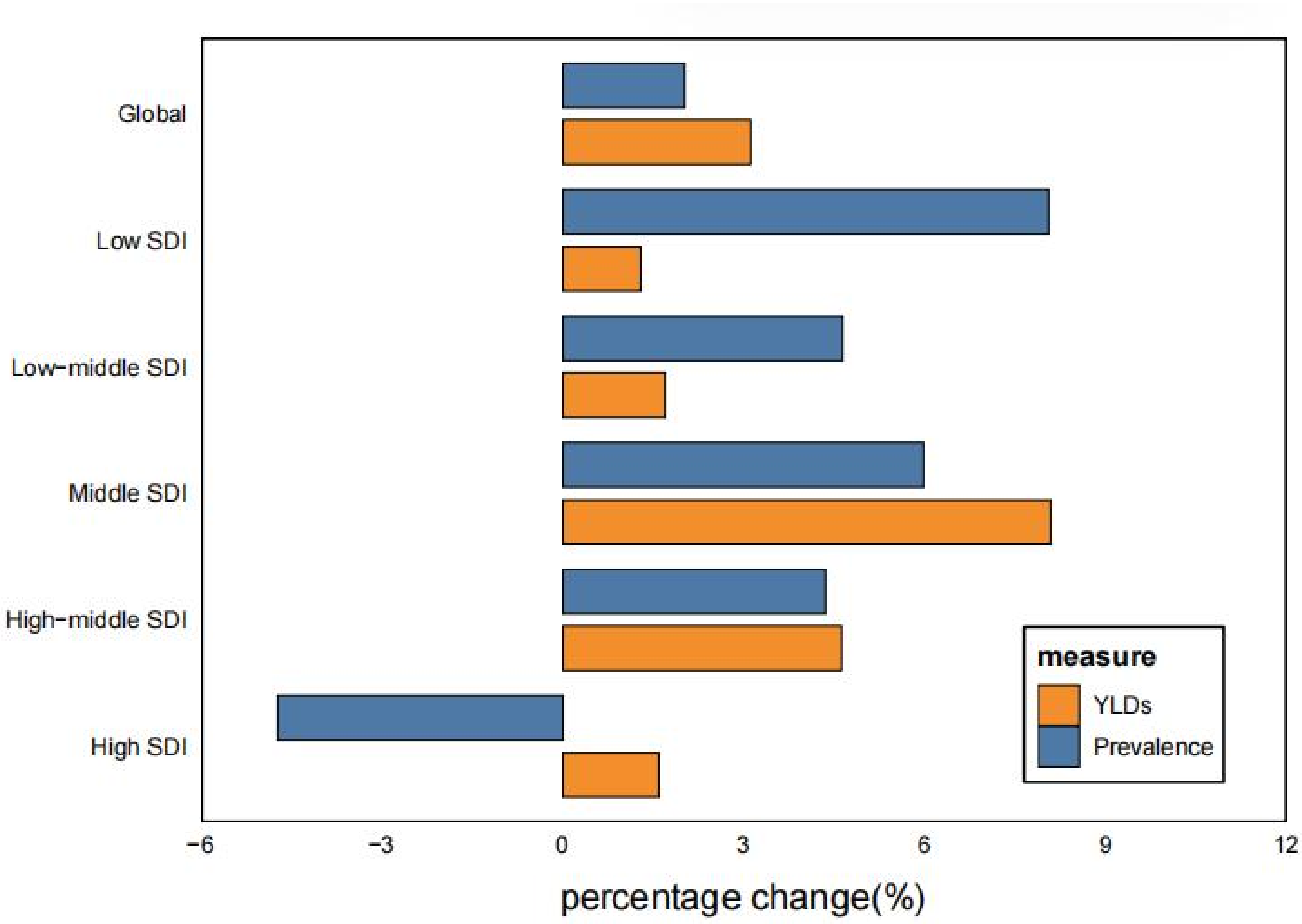

Supplementary Figure 3. Percentage Change in Age-Standardized YLDs and Prevalence Rates for Tension-Type Headache by Region（1990-2021）

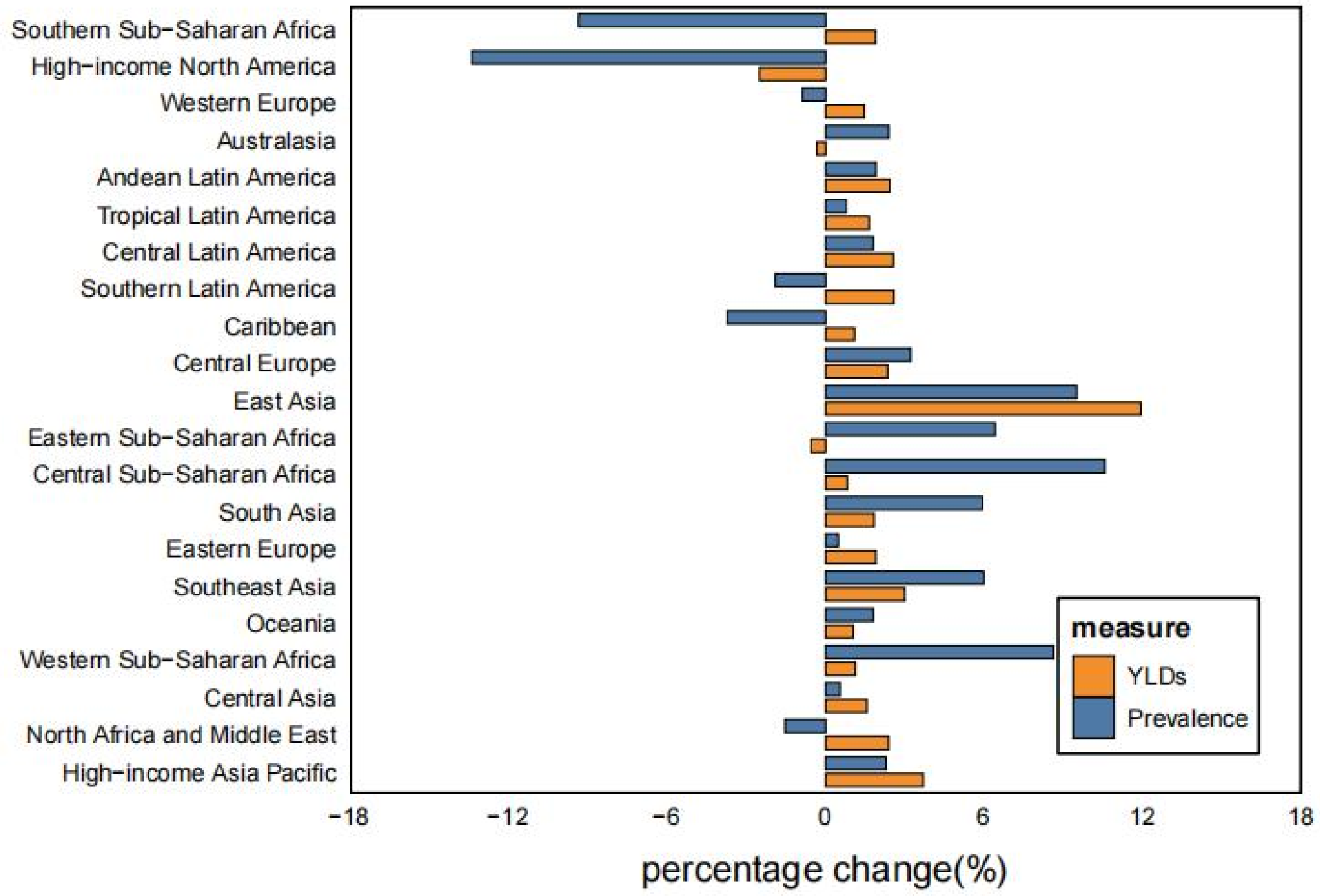

Supplementary Figure 4. Percentage Change in Age-Standardized YLDs and Prevalence Rates for Tension-Type Headache by SDI quintiles level（1990-2021）

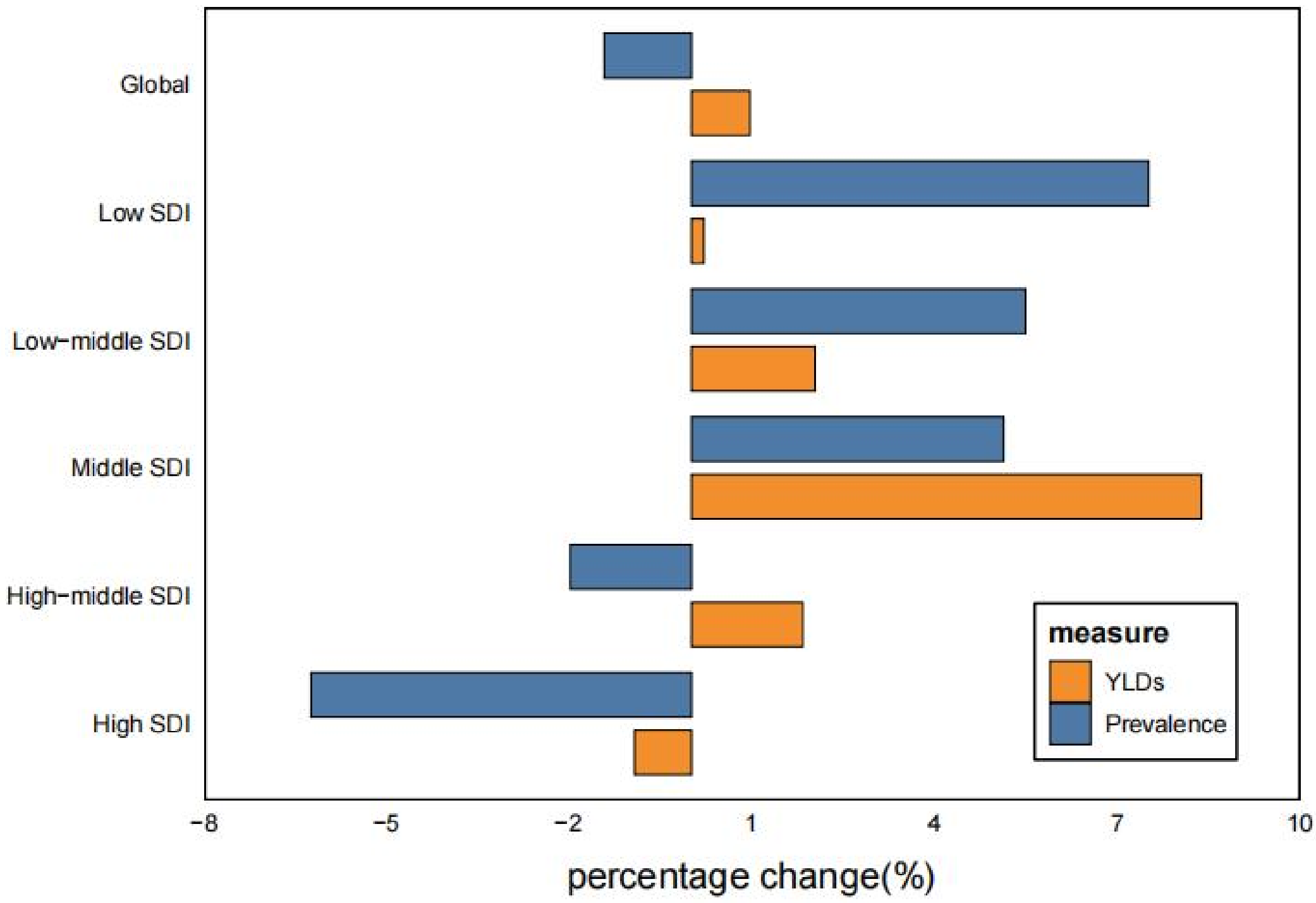

Supplementary Figure 5. Percentage Change in All-Age YLDs and Prevalence Rates for Migraine by Region（1990-2021）

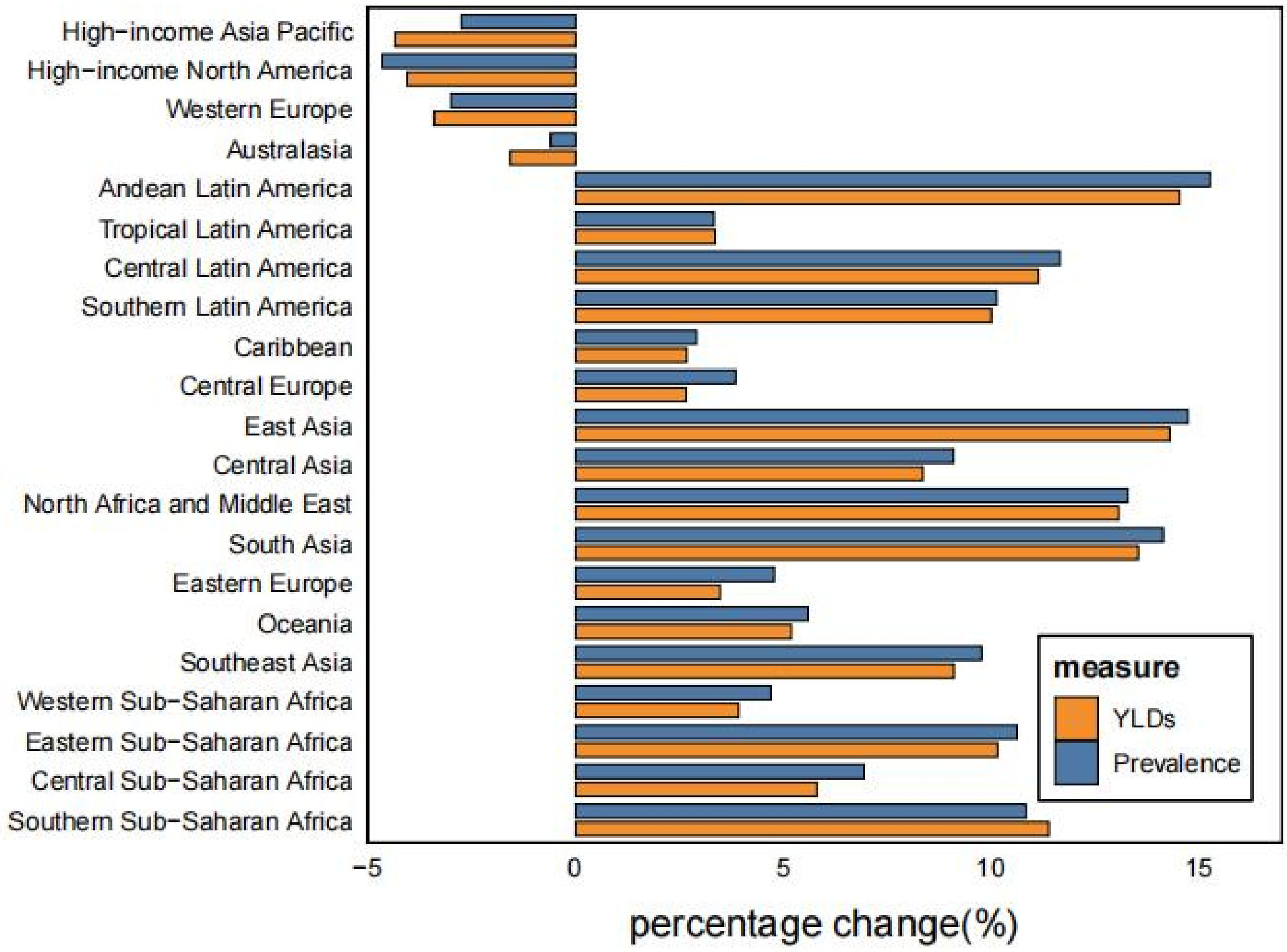

Supplementary Figure 6. Percentage Change in All-Age YLDs and Prevalence Rates for Migraine by SDI quintiles level （1990-2021）

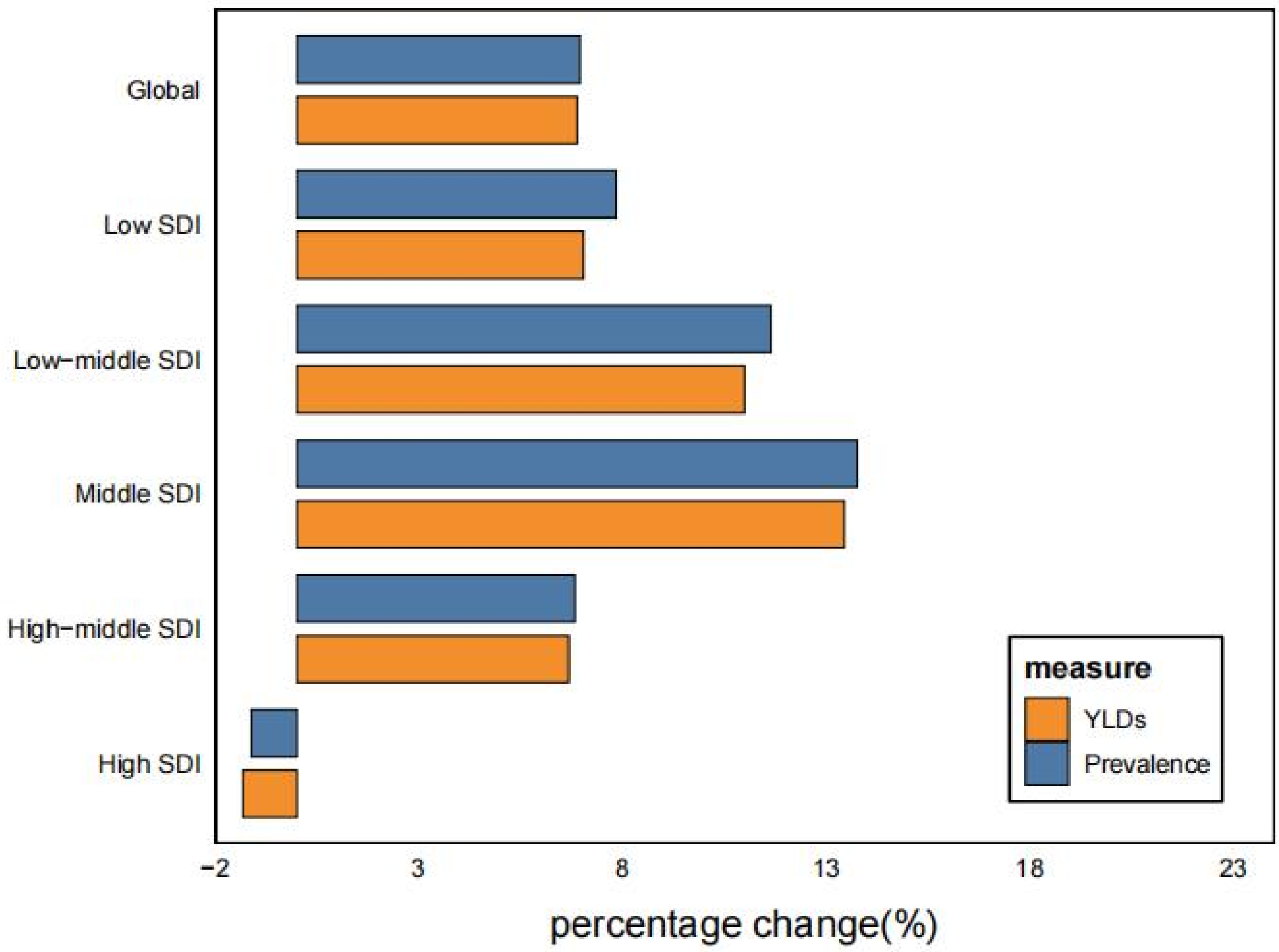

Supplementary Figure 7. Percentage Change in All-Age YLDs and Prevalence Rates for Tension-Type Headache by Region（1990-2021）

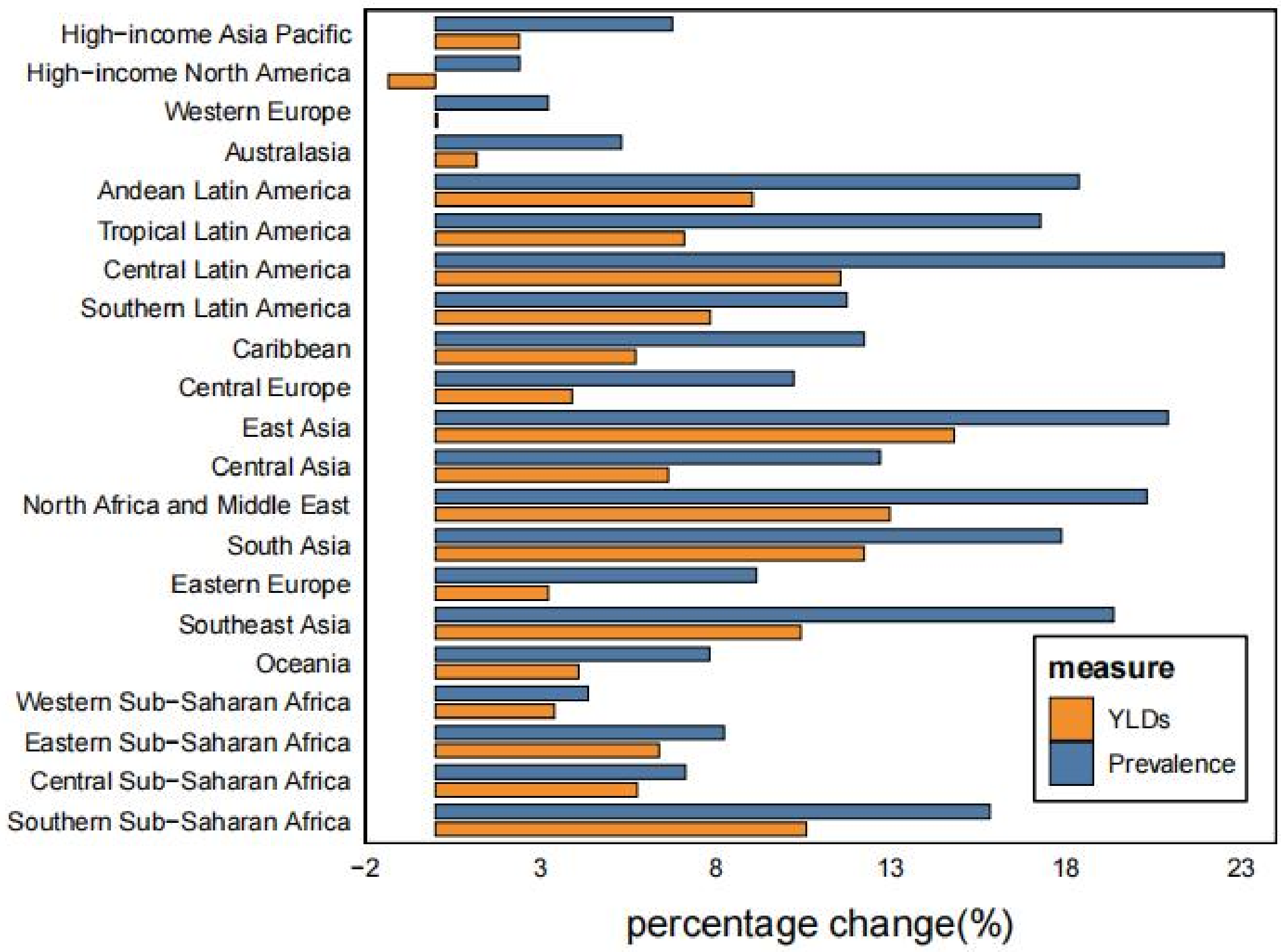

Supplementary Figure 8. Percentage Change in All-Age YLDs and Prevalence Rates for Tension-Type Headache by SDI quintiles level （1990-2021）

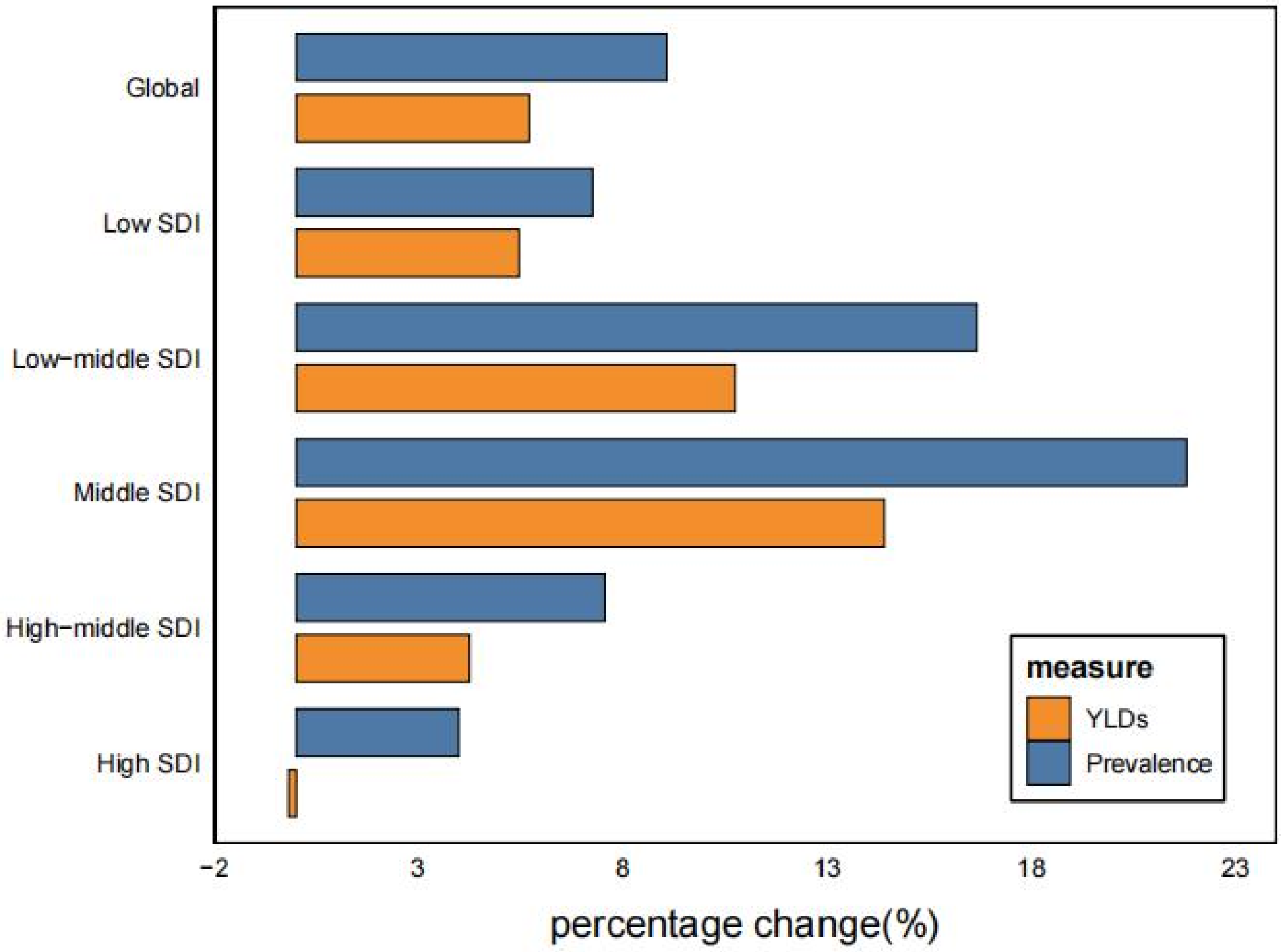

Supplementary Figure 9. Prevalence of Migraine by Gender and Age Group

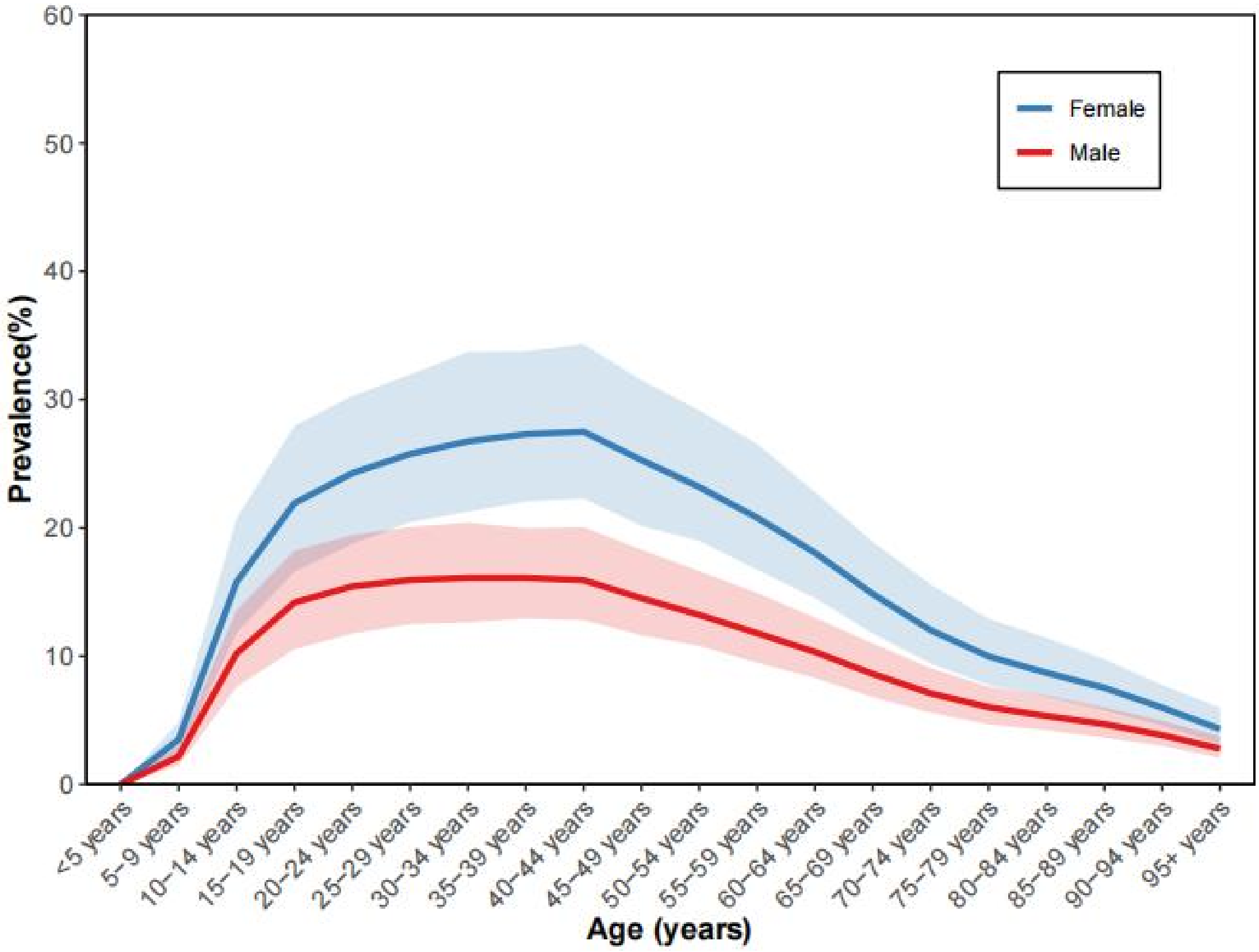

Supplementary Figure 10. YLDs of Migraine by Gender and Age Group

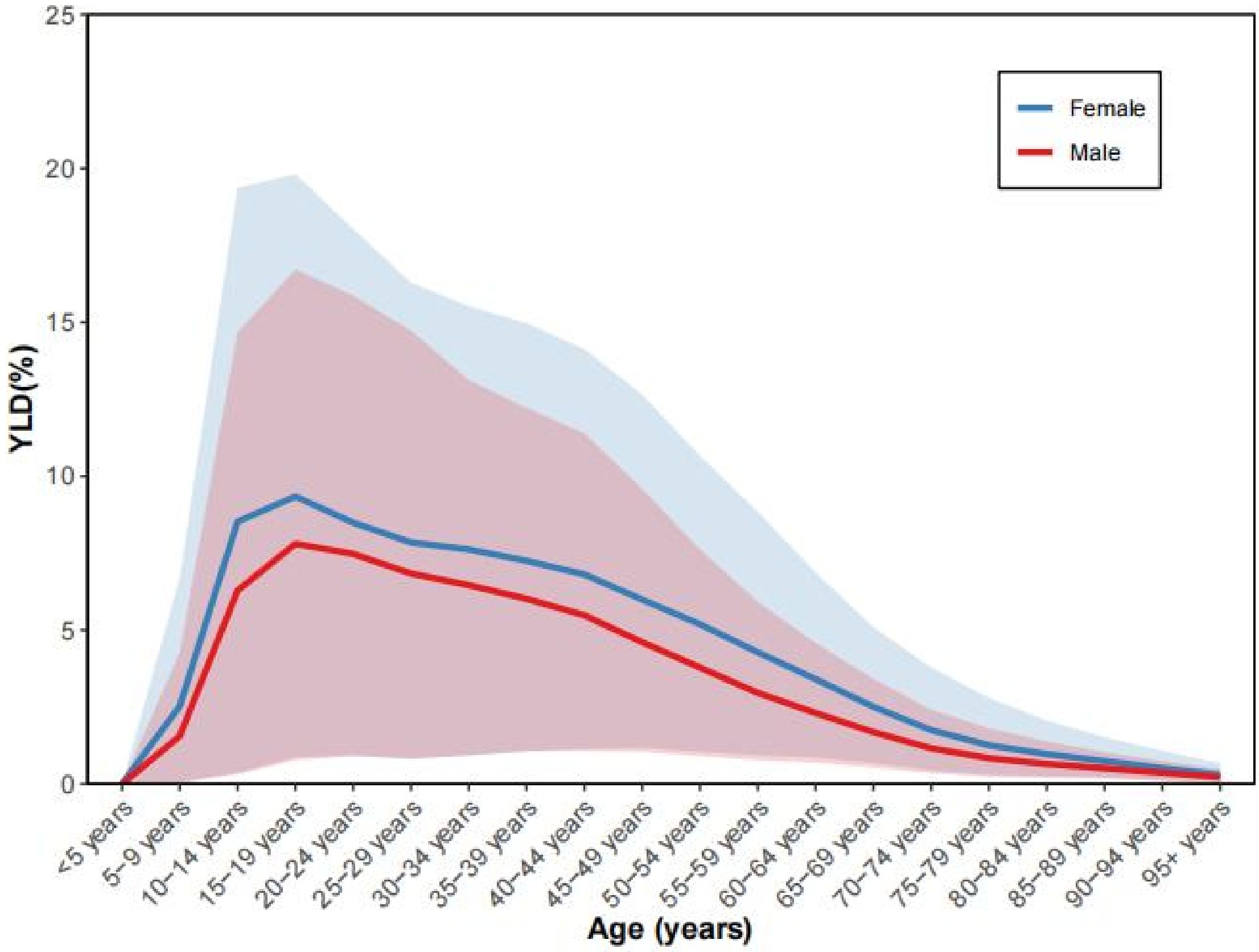

Supplementary Figure 11. Prevalence of Tension-Type Headache by Gender and Age Group

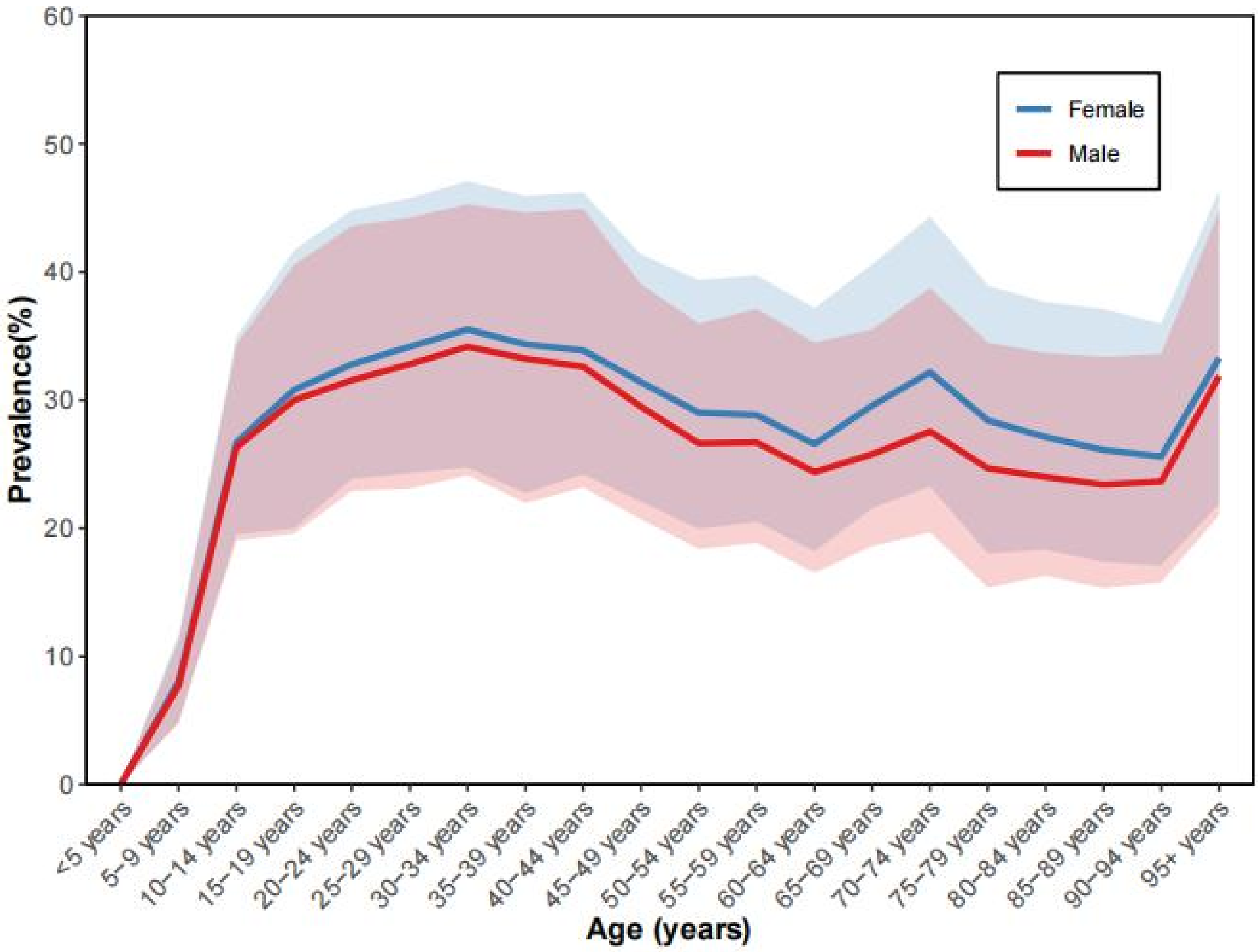

Supplementary Figure 12. YLDs of Tension-Type Headache by Gender and Age Group

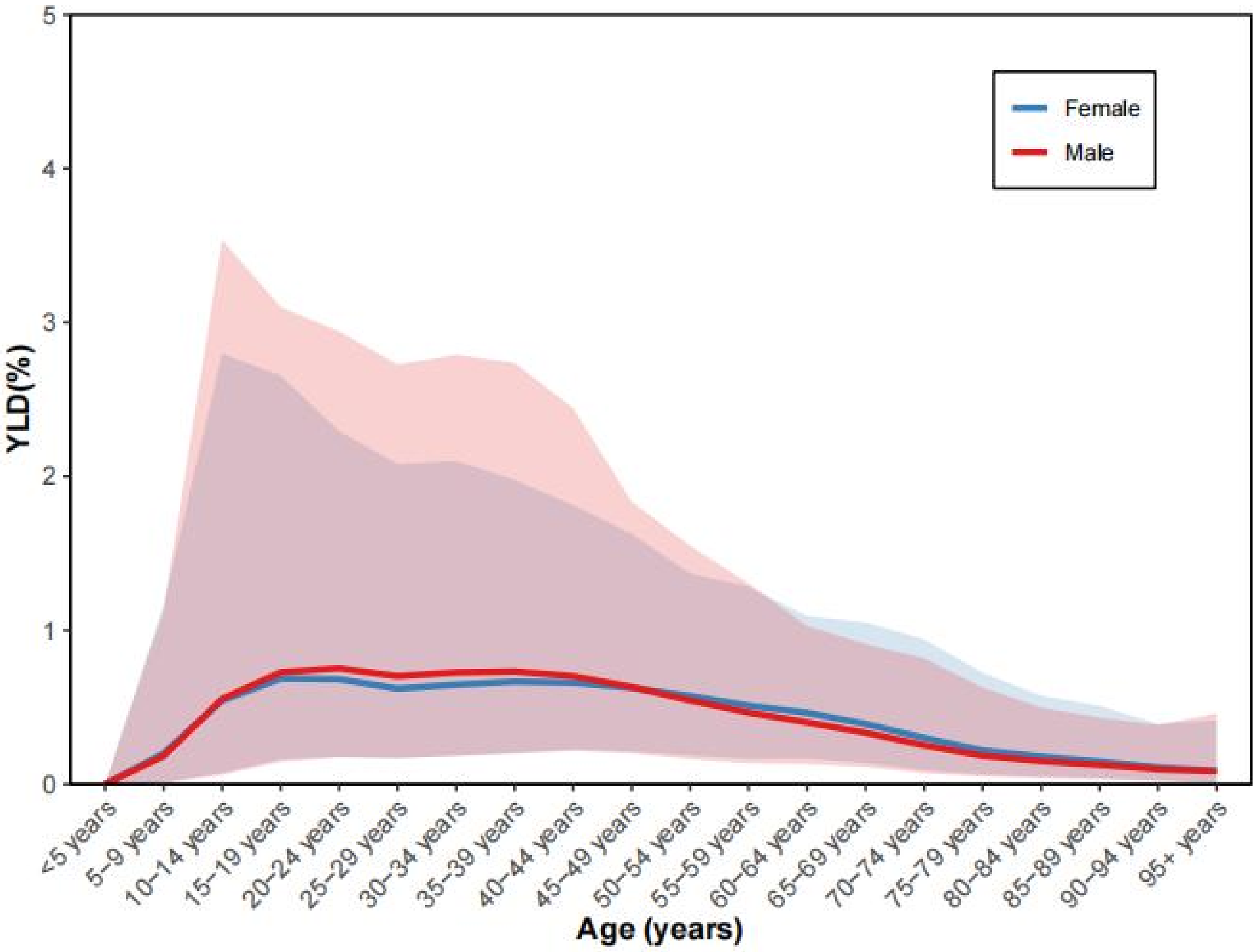

Supplementary Figure 13. Age-Period-Cohort (APC) Model Analysis of Migraine Incidence Number: Epidemiological Characteristics of Age Effects, Period Trends, and Cohort Differences

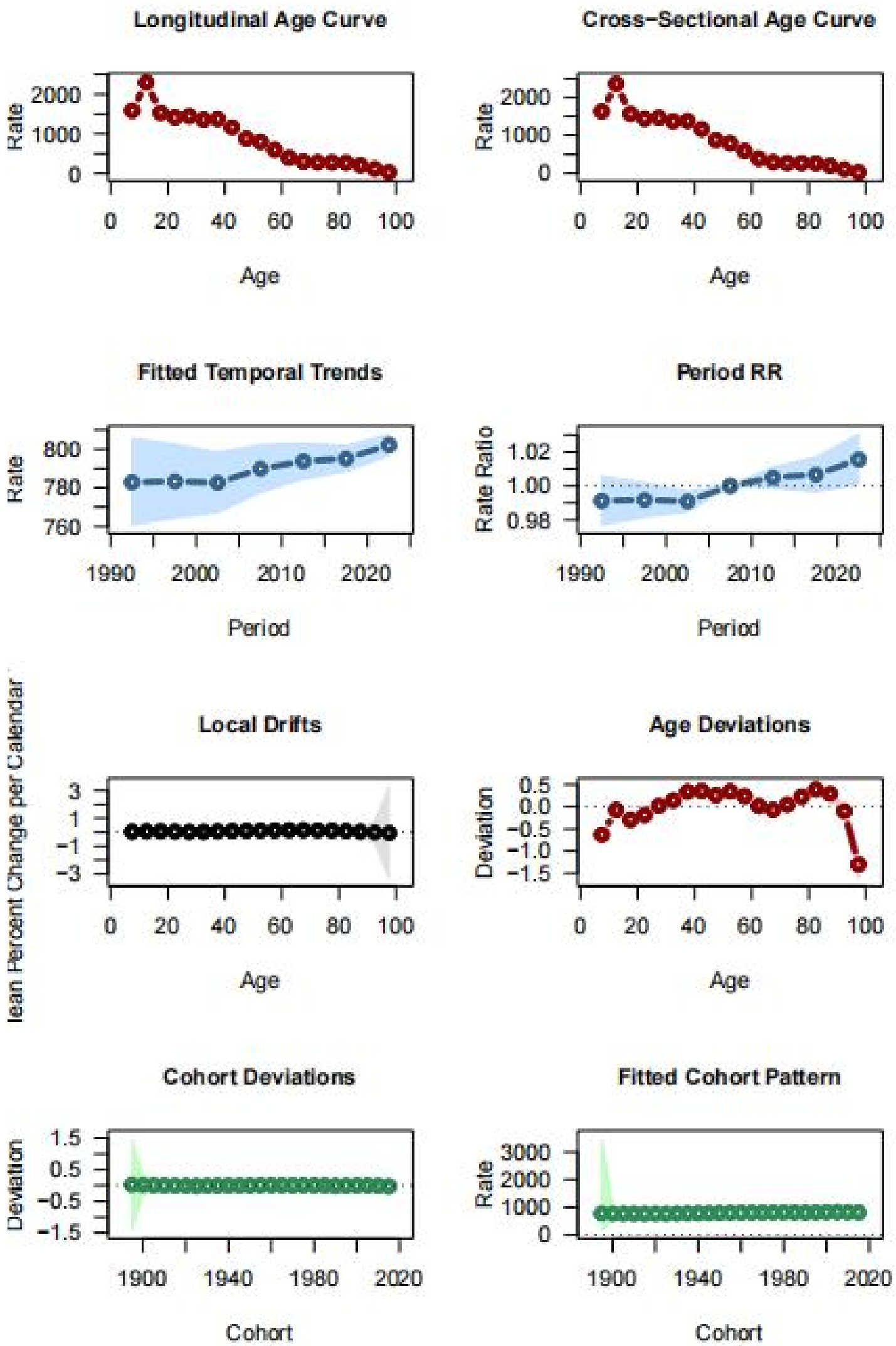

Supplementary Figure 14. Age-Period-Cohort (APC) Model Analysis of Migraine Incidence Rate: Epidemiological Characteristics of Age Effects, Period Trends, and Cohort Differences

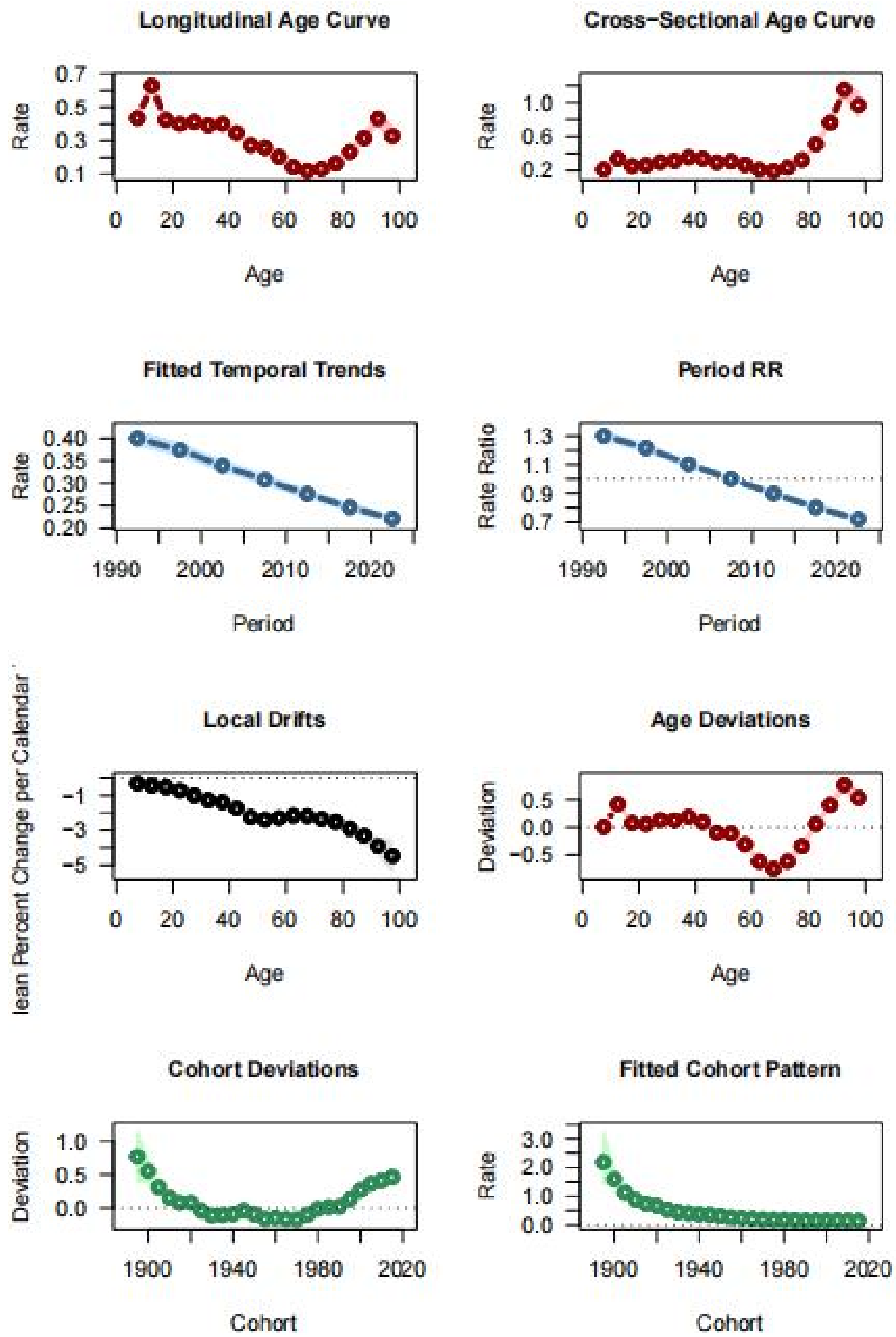

Supplementary Figure 15. Age, Period, and Cohort Effects on Migraine Incidence Rate: A Comprehensive Analysis from 1900 to 2020

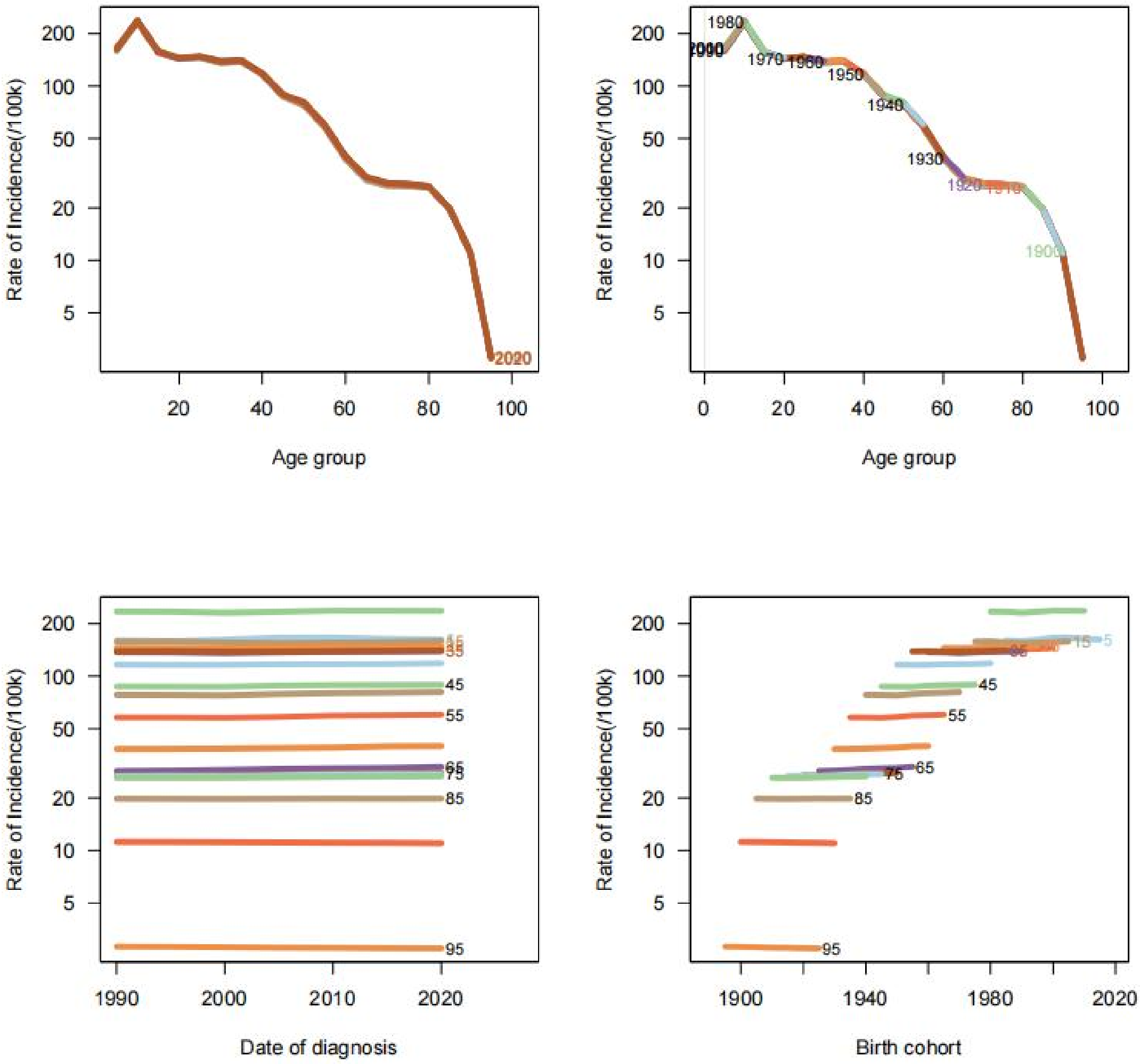

Supplementary Figure 16. Age-Period-Cohort (APC) Model Analysis of Migraine Incidence Percent: Epidemiological Characteristics of Age Effects, Period Trends, and Cohort Differences

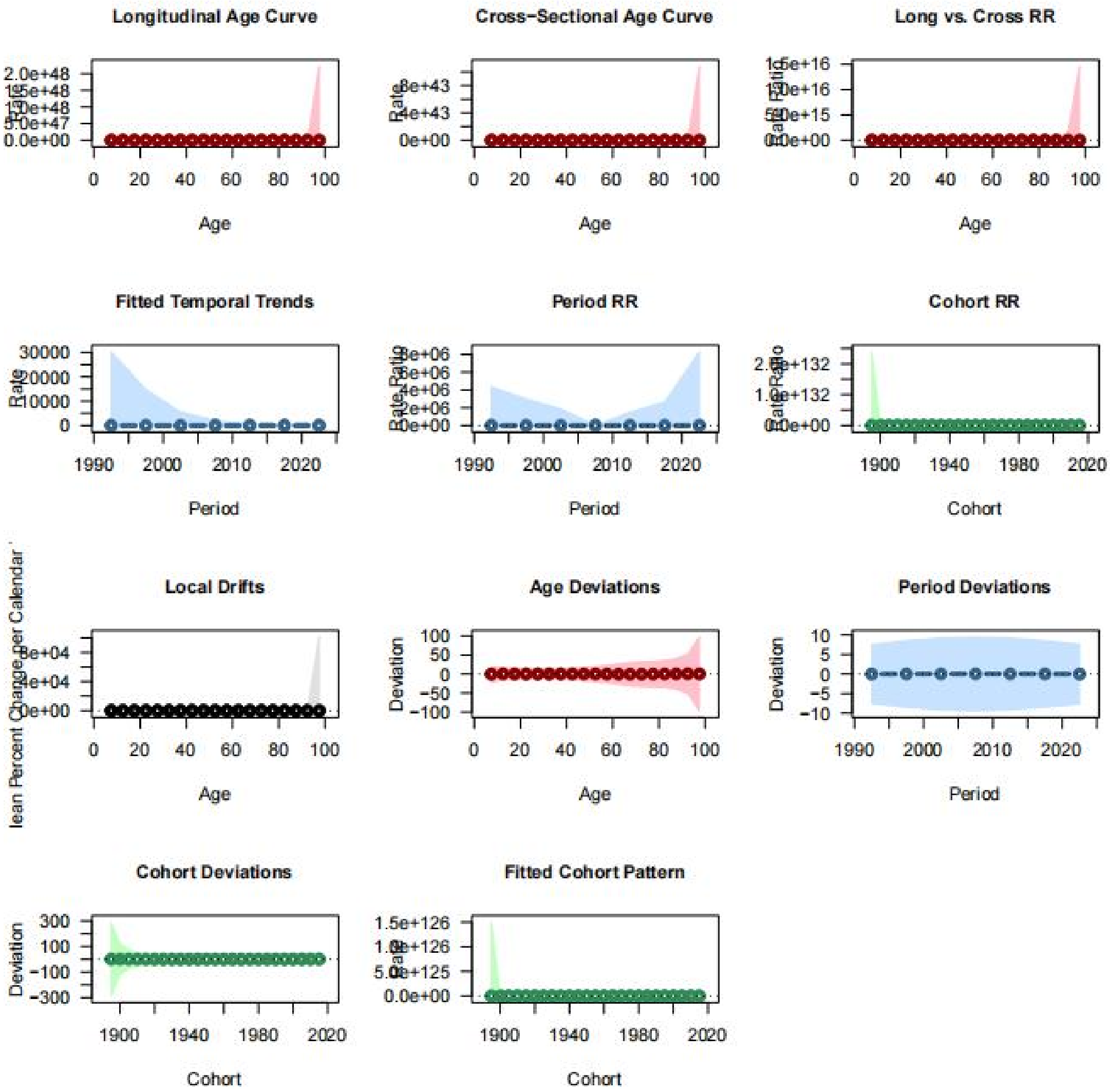

Supplementary Figure 17. Age, Period, and Cohort Effects on Migraine Incidence Percent: A Comprehensive Analysis from 1900 to 2020

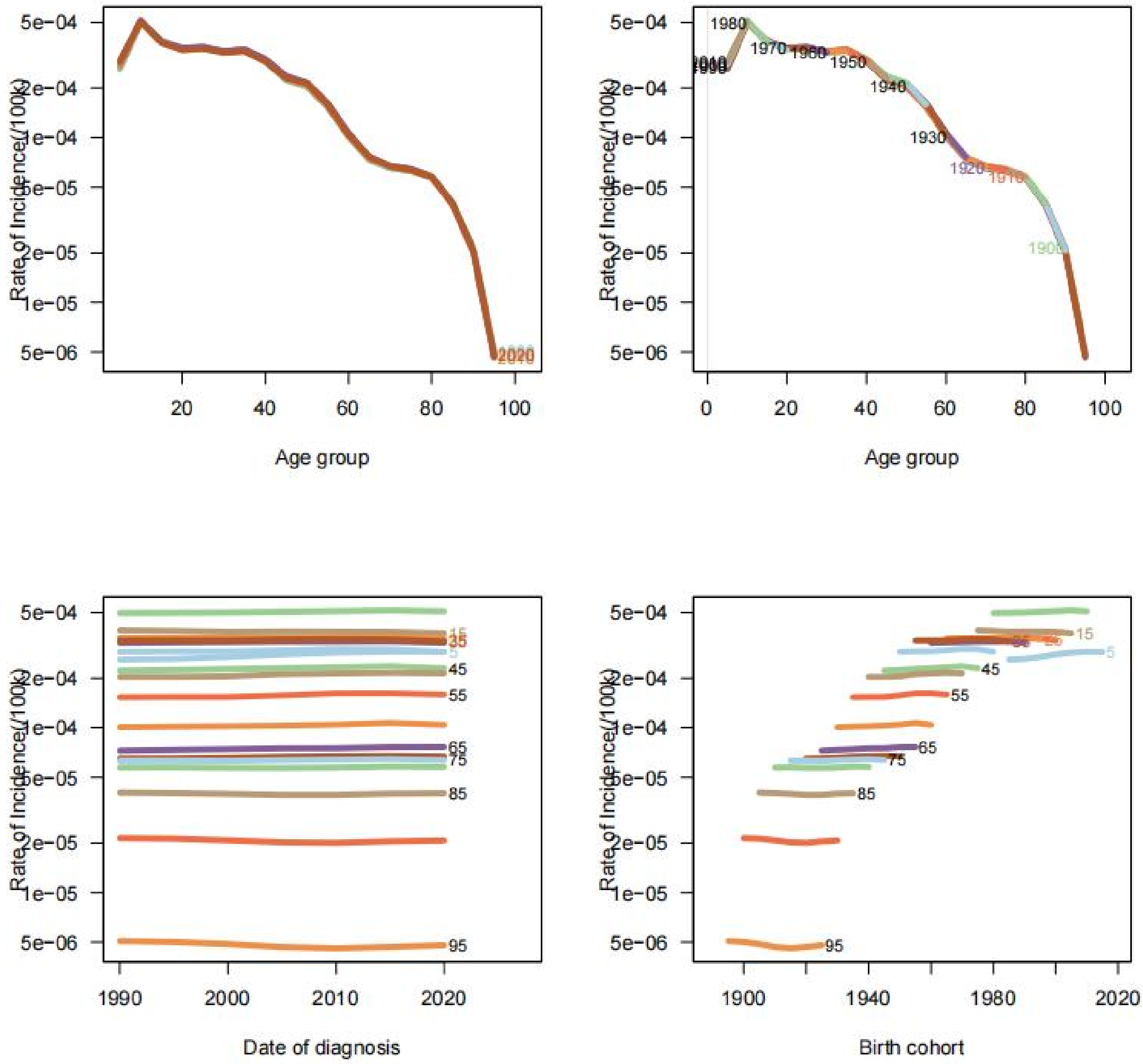

Supplementary Figure 18. Age-Period-Cohort (APC) Model Analysis of Tension-Type Headache Incidence Number: Epidemiological Characteristics of Age Effects, Period Trends, and Cohort Differences

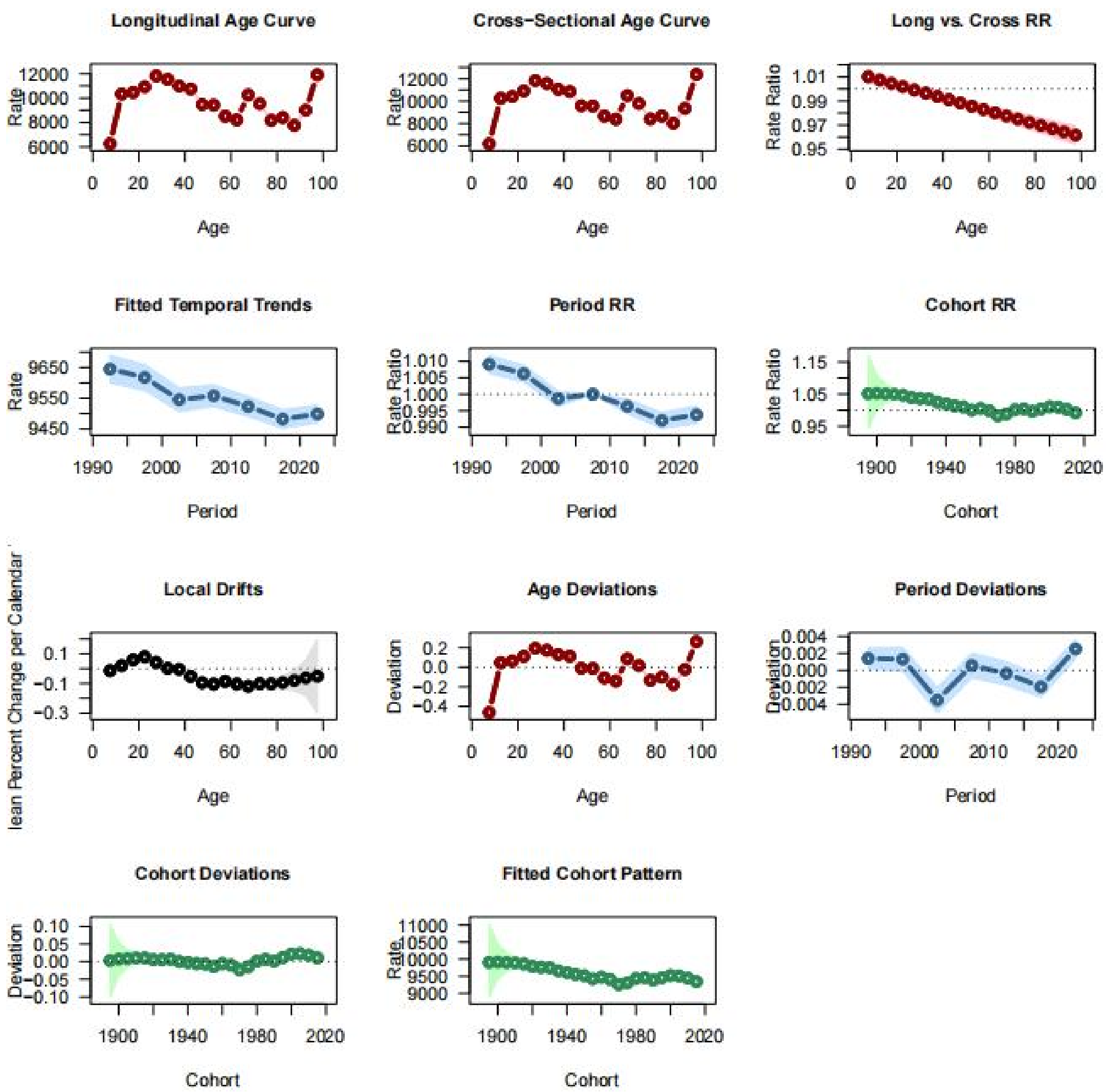

Supplementary Figure 19. Age, Period, and Cohort Effects on Tension-Type Headache Incidence Number: A Comprehensive Analysis from 1900 to 2020

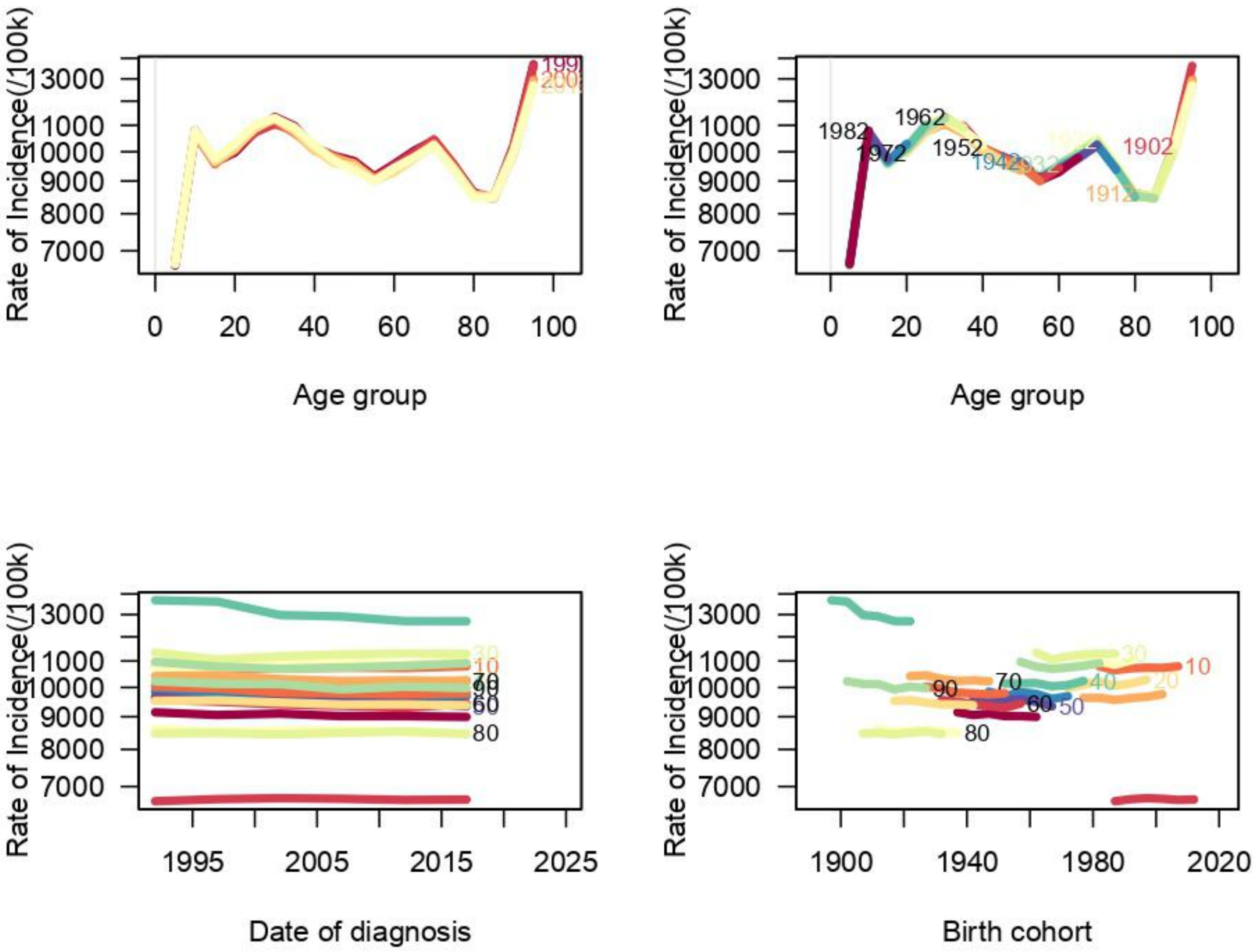

Supplementary Figure 20. Age-Period-Cohort (APC) Model Analysis of Tension-Type Headache Incidence Rate: Epidemiological Characteristics of Age Effects, Period Trends, and Cohort Differences

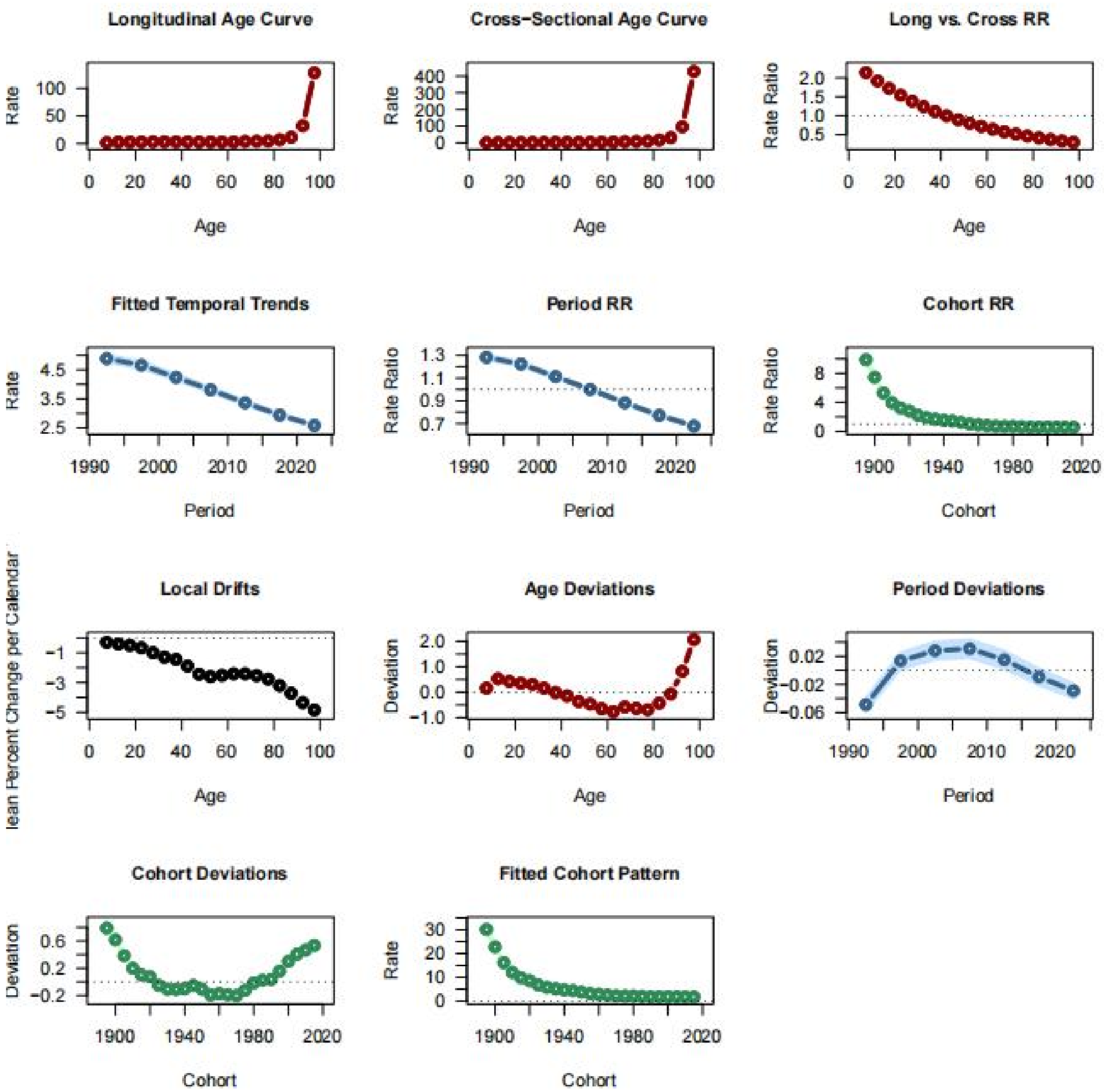

Supplementary Figure 21. Age, Period, and Cohort Effects on Tension-Type Headache Incidence Rate: A Comprehensive Analysis from 1900 to 2020

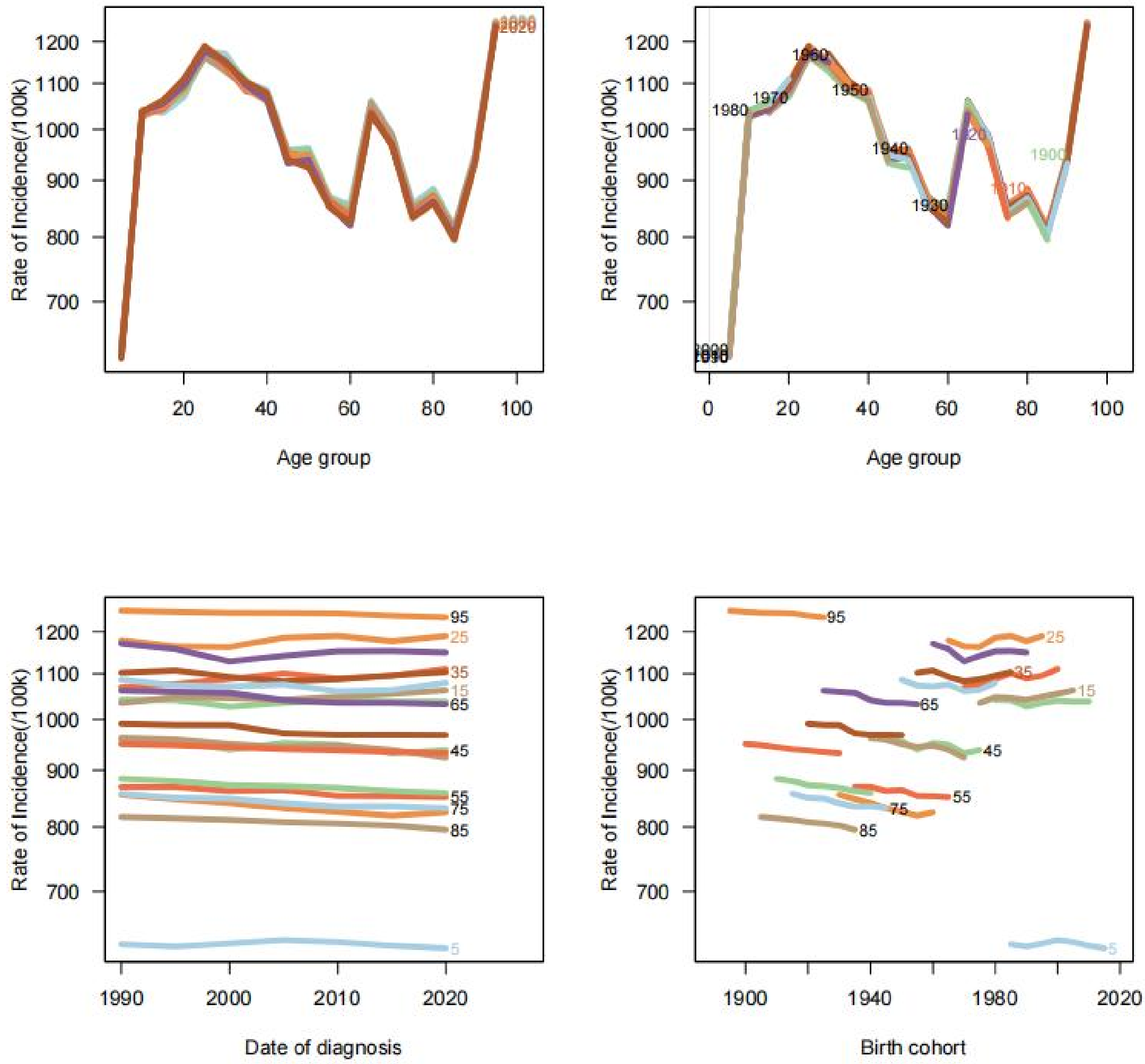

Supplementary Figure 22. Age-Period-Cohort (APC) Model Analysis of Tension-Type Headache Incidence Percent: Epidemiological Characteristics of Age Effects, Period Trends, and Cohort Differences

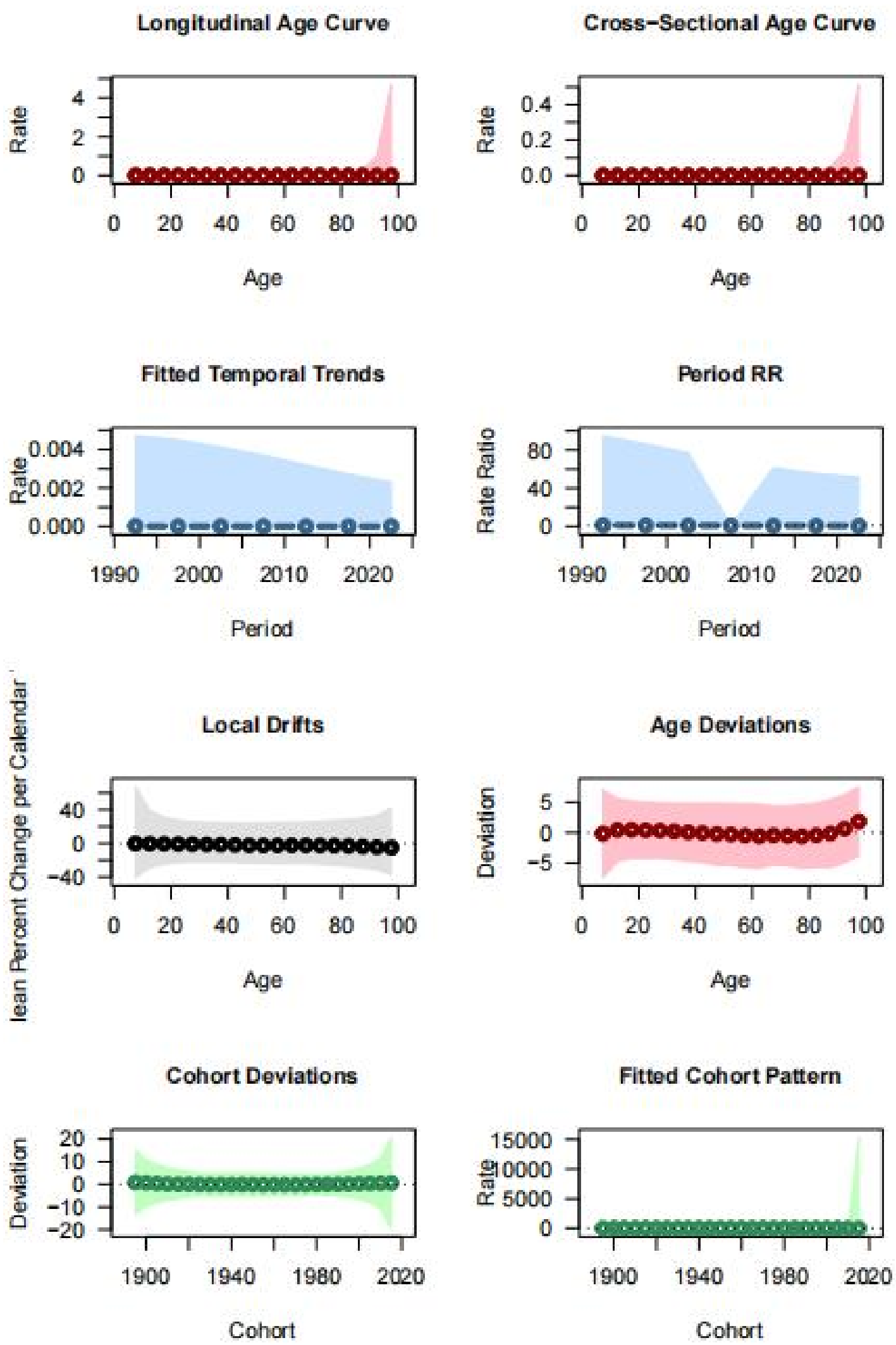

Supplementary Figure 23. Age, Period, and Cohort Effects on Tension-Type Headache Incidence Percent: A Comprehensive Analysis from 1900 to 2020

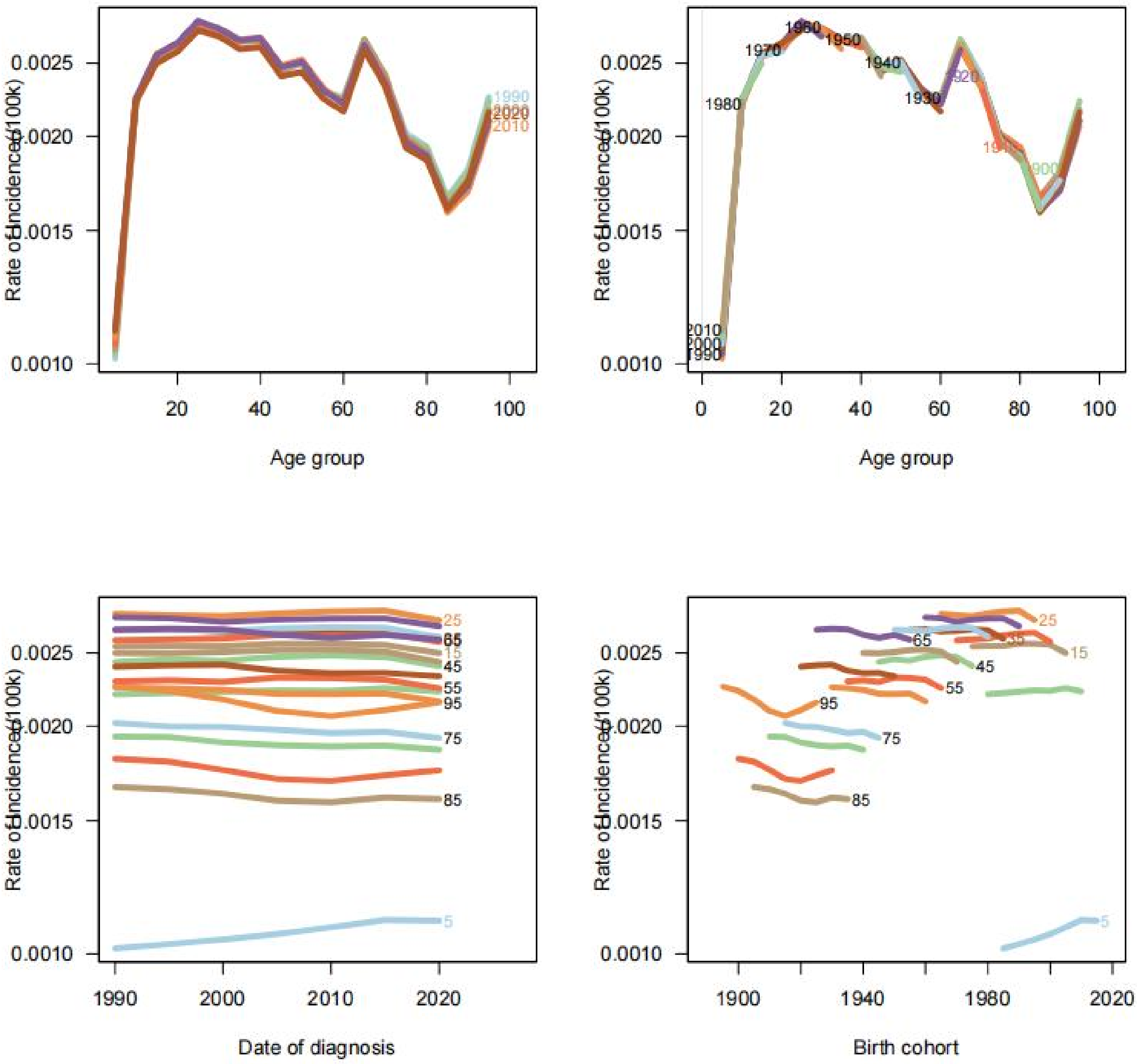

Supplementary Figure 24. Age-standardised disability-adjusted-life-years (DALY) rates for Migraine by 21 Global Burden of Disease regions by Socio-demographic Index (SDI), 1990–2021

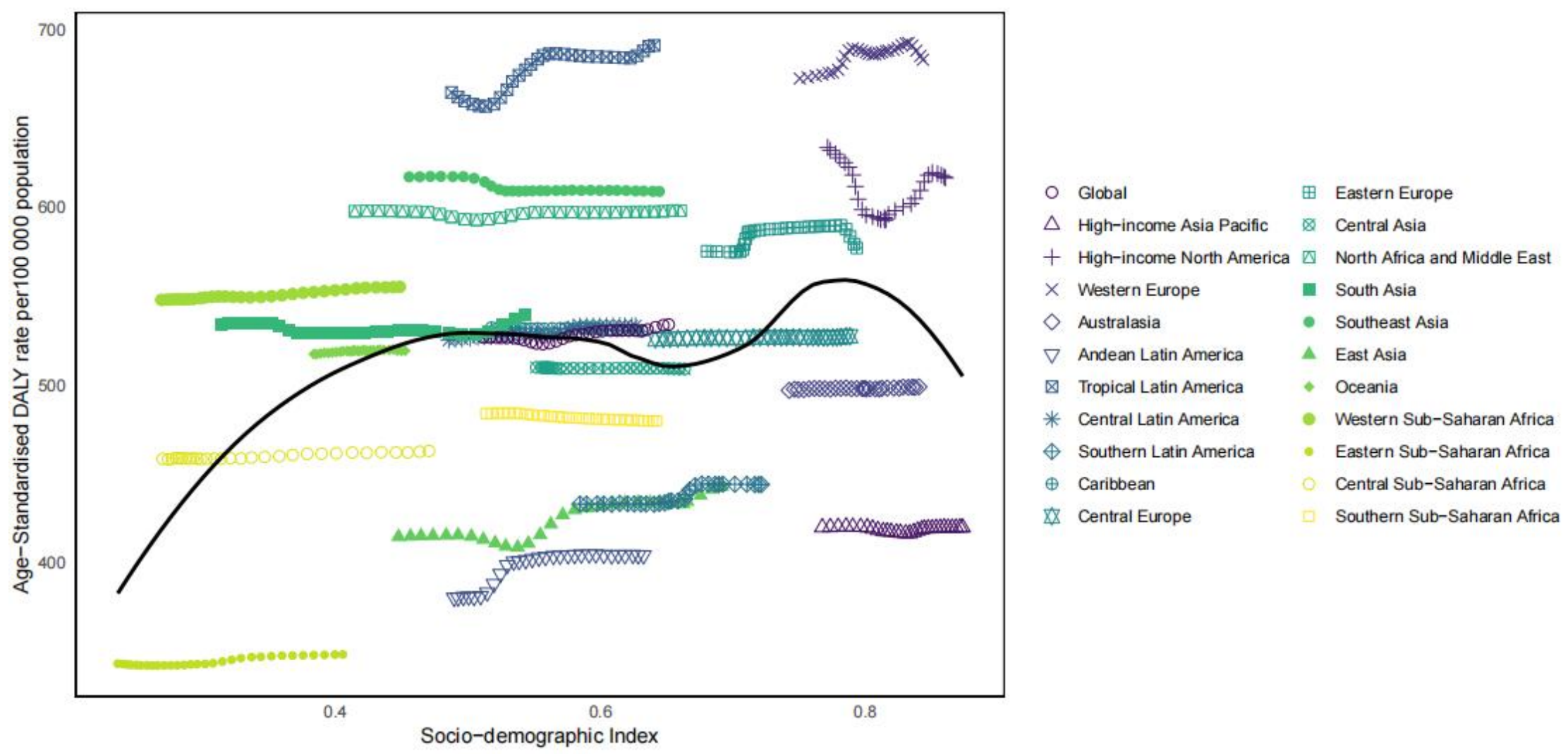

Supplementary Figure 25. Age-standardised disability-adjusted-life-years (DALY) rates for Tension-Type Headache by 21 Global Burden of Disease regions by Socio-demographic Index (SDI), 1990–2021

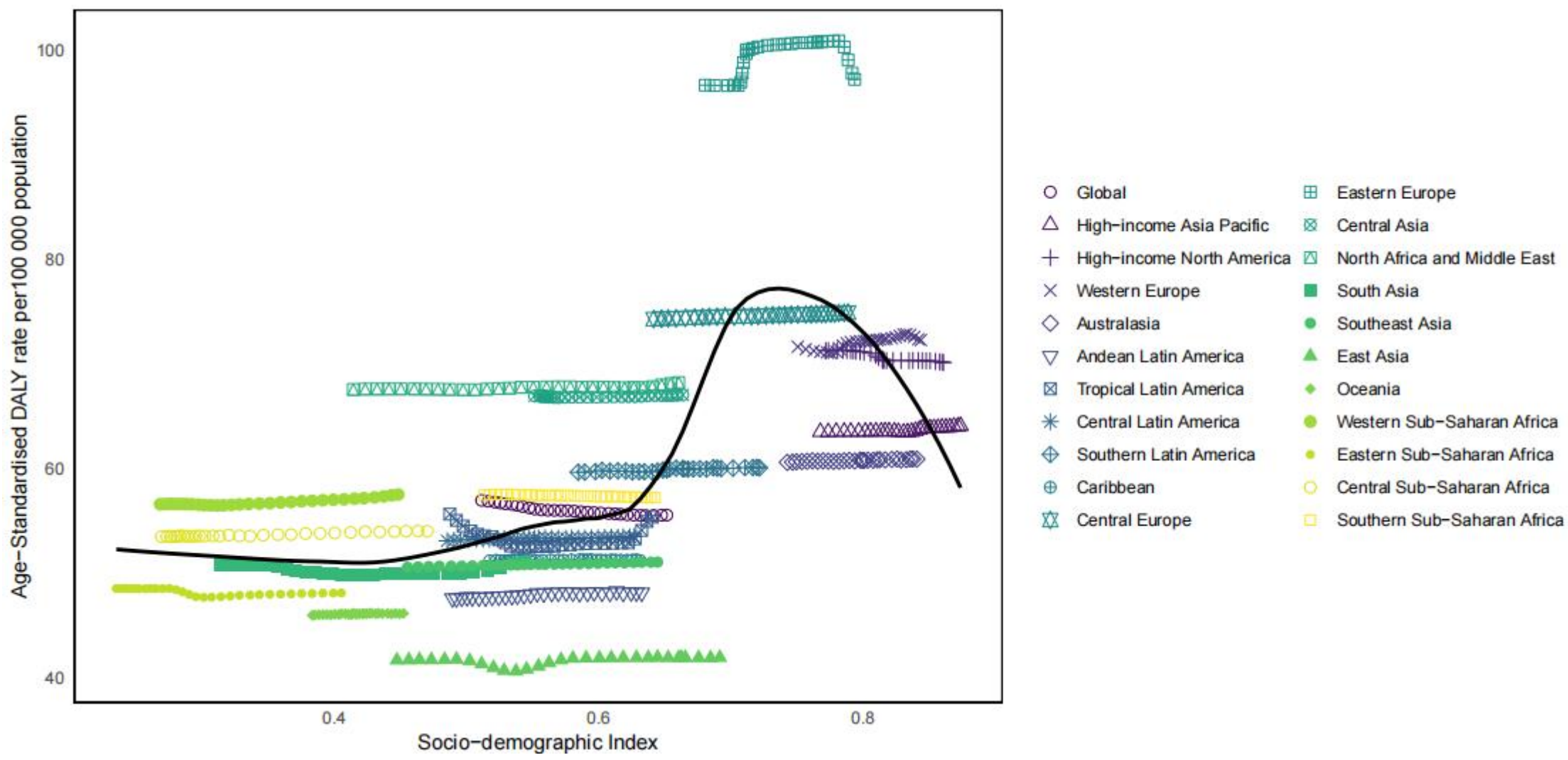

Supplementary Figure 26. Forecasting YLDs for Migraine in the Global Female Population Across All Age Groups Using the Holt-Winters Algorithm

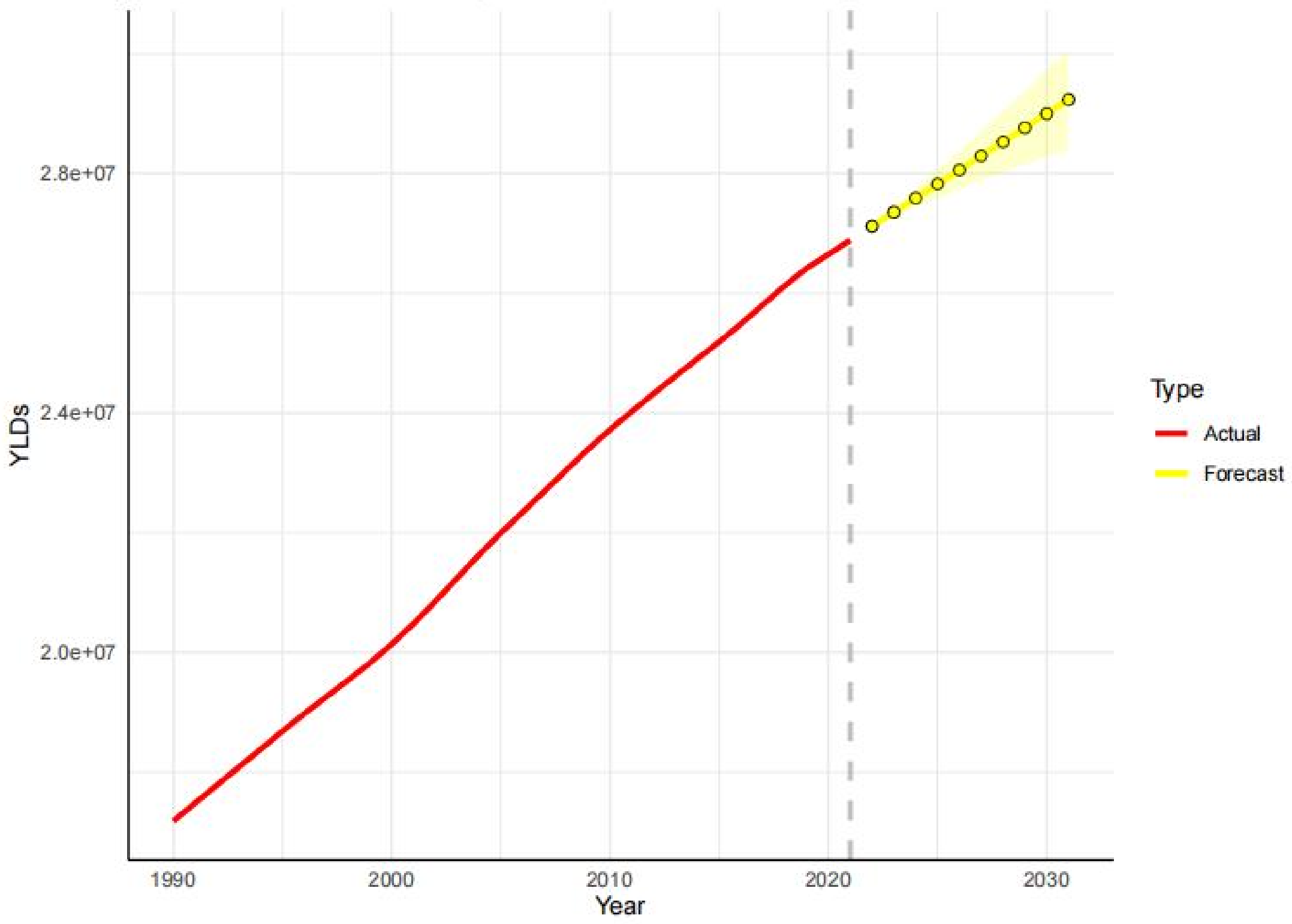

Supplementary Figure 27. Forecasting YLDs for Migraine in the Global Female Population Across All Age Groups Using the ARIMA Algorithm

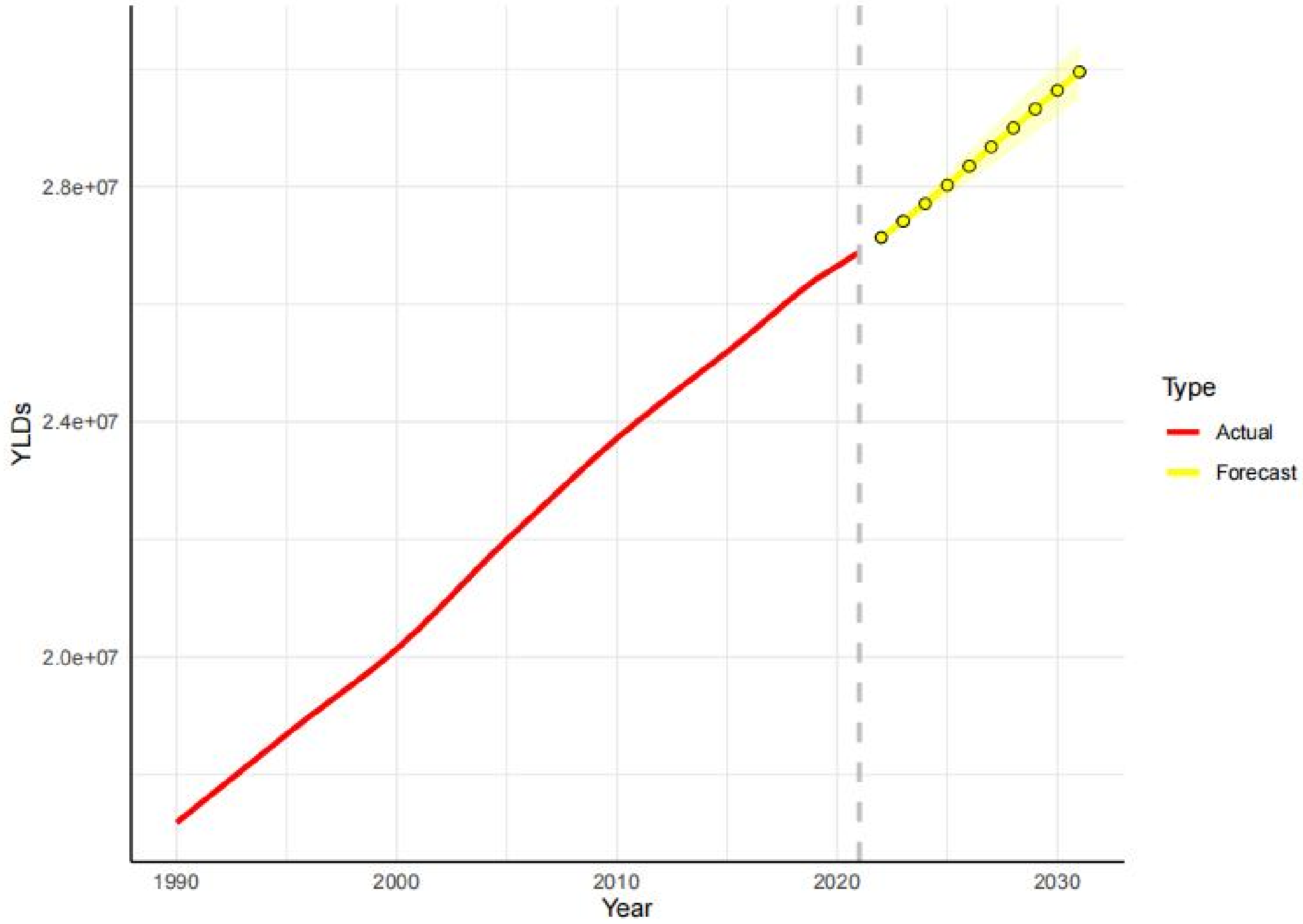

Supplementary Figure 28. Forecasting Incidence for Migraine in the Global Female Population Across All Age Groups Using the Holt-Winters Algorithm

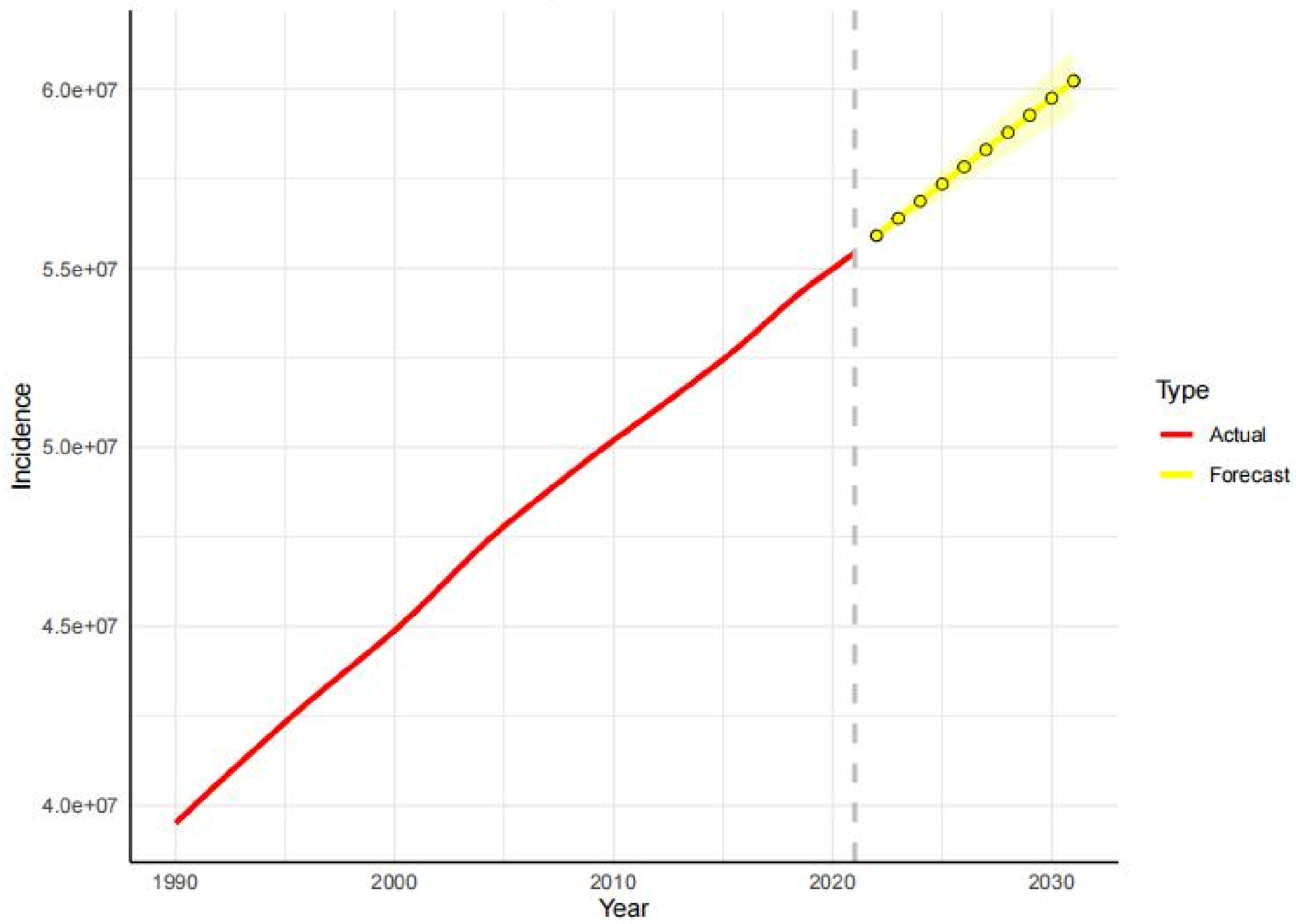

Supplementary Figure 29. Forecasting Incidence for Migraine in the Global Female Population Across All Age Groups Using the ARIMA Algorithm

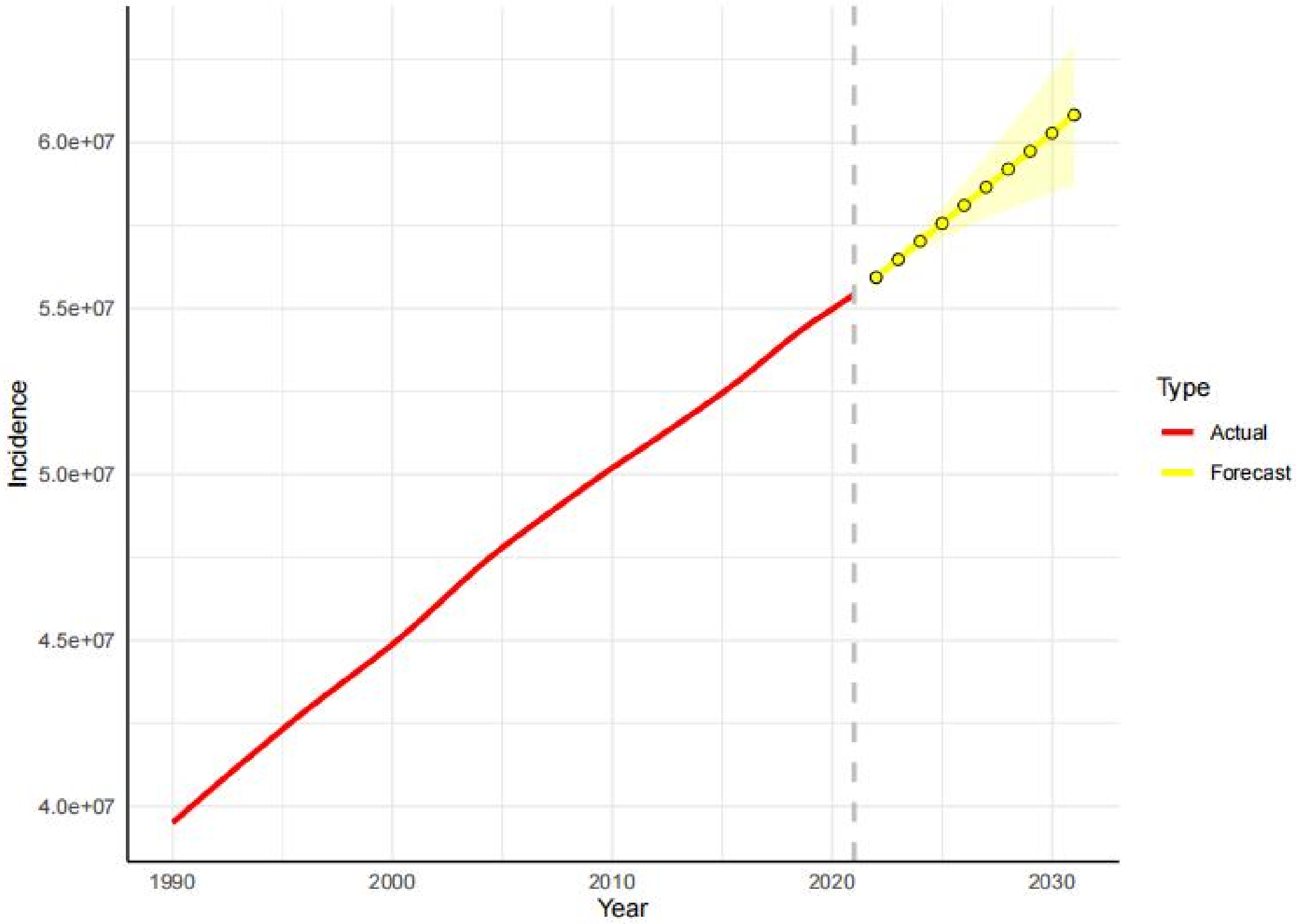

Supplementary Figure 30. Forecasting YLDs for Tension-Type Headache in the Global Female Population Across All Age Groups Using the Holt-Winters Algorithm

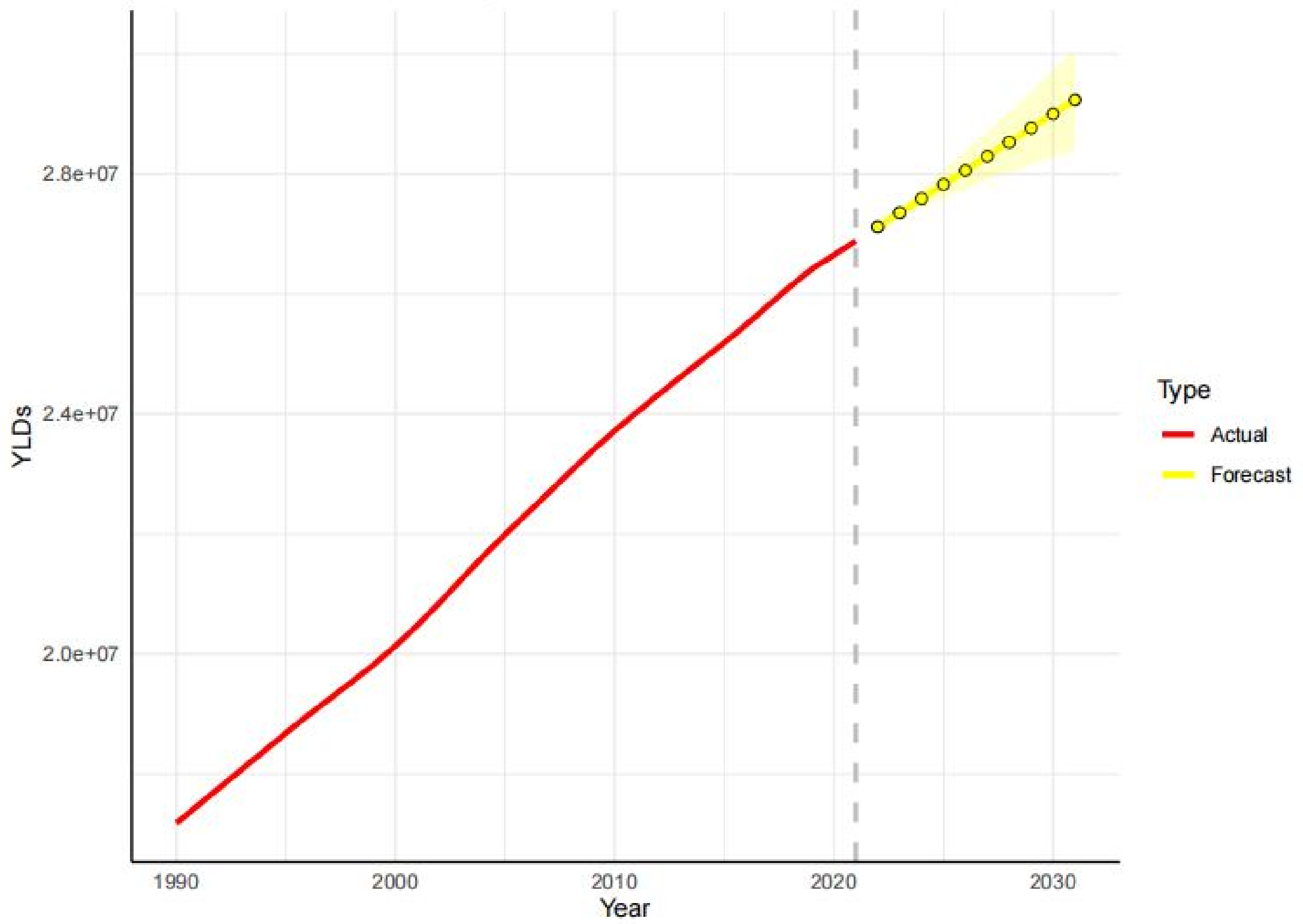

Supplementary Figure 31. Forecasting YLDs for Tension-Type Headache in the Global Female Population Across All Age Groups Using the ARIMA Algorithm

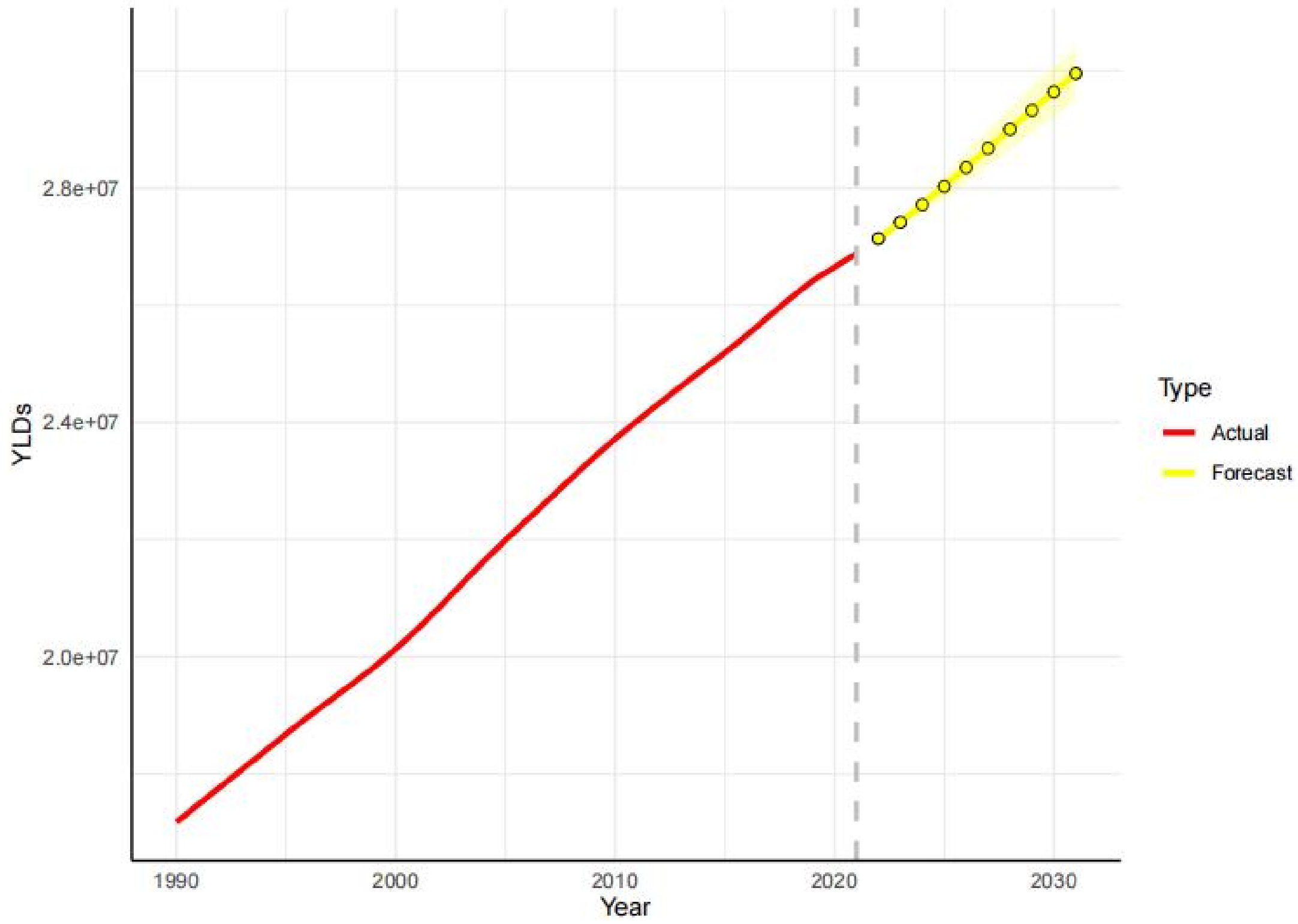

Supplementary Figure 32. Forecasting Incidence for Tension-Type Headache in the Global Female Population Across All Age Groups Using the Holt-Winters Algorithm

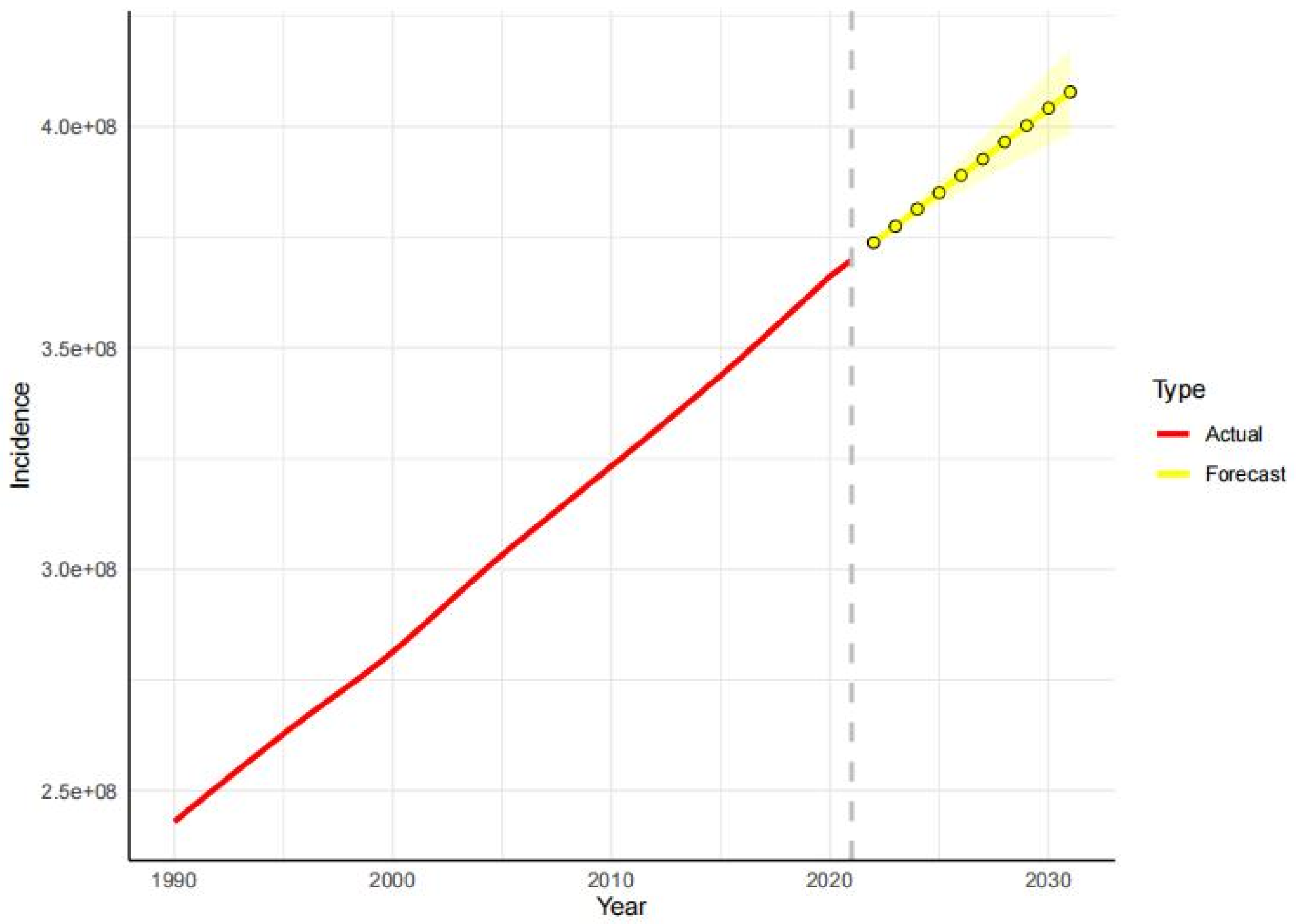

Supplementary Figure 33. Forecasting Incidence for Tension-Type Headache in the Global Female Population Across All Age Groups Using the ARIMA Algorithm

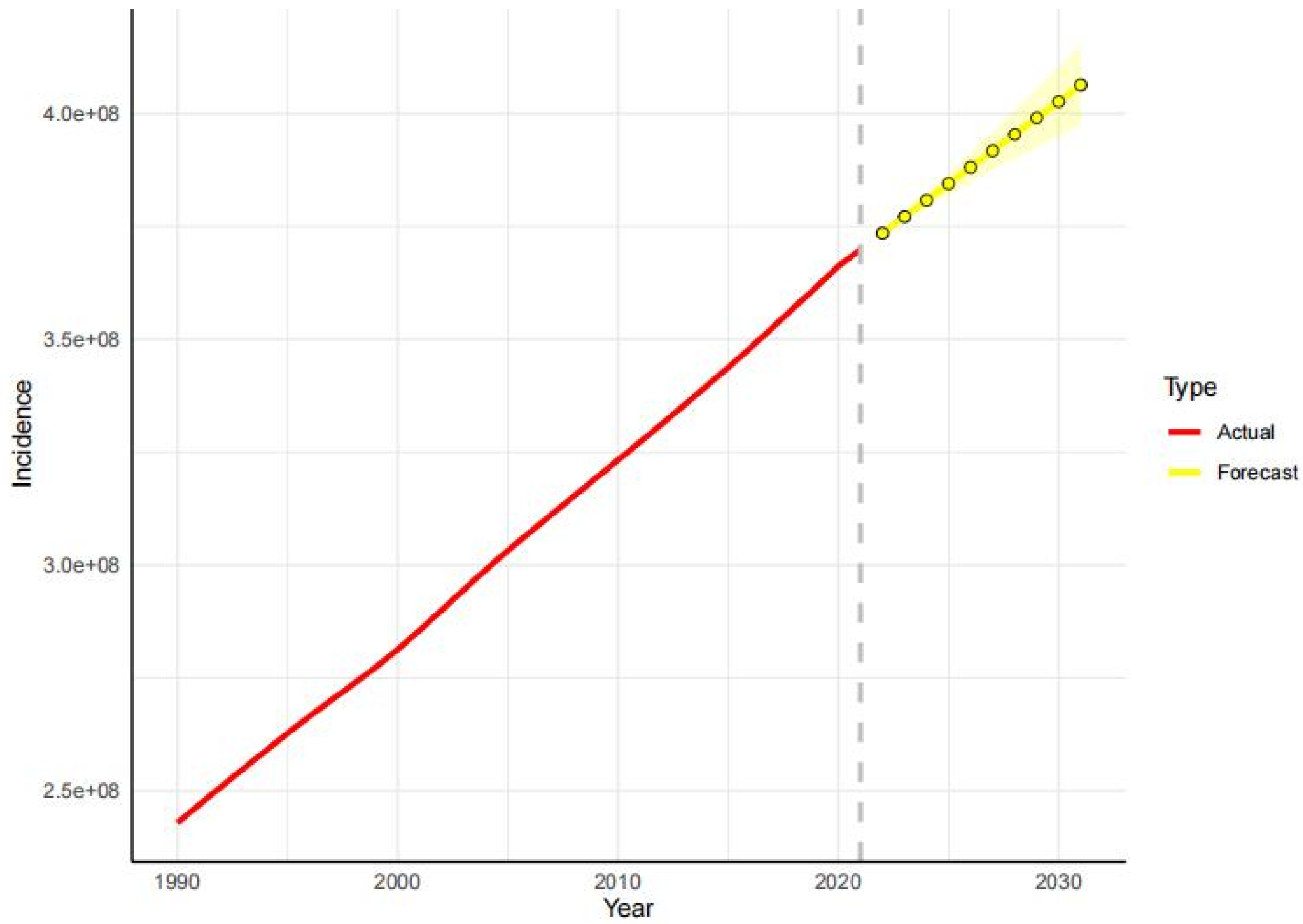

Supplementary Figure 34. The Crude Incidence of Migraine by Socio-demographic Index (SDI) in 1990 and 2021

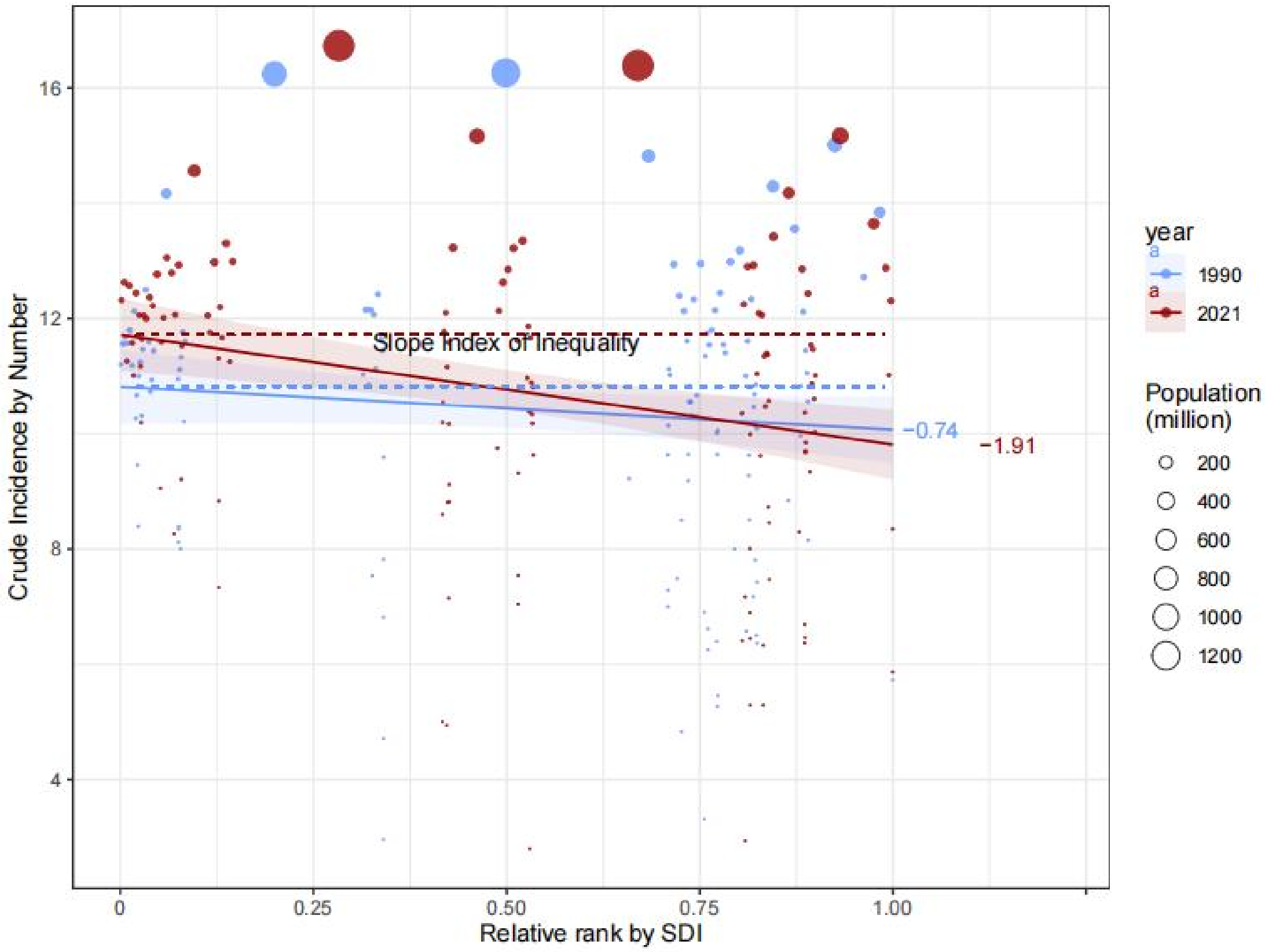

Supplementary Figure 35. **The Cumulative Fraction of Migraine Incidence by Cumulative Fraction of Population Ranked by SDI in 1990 and 2021**

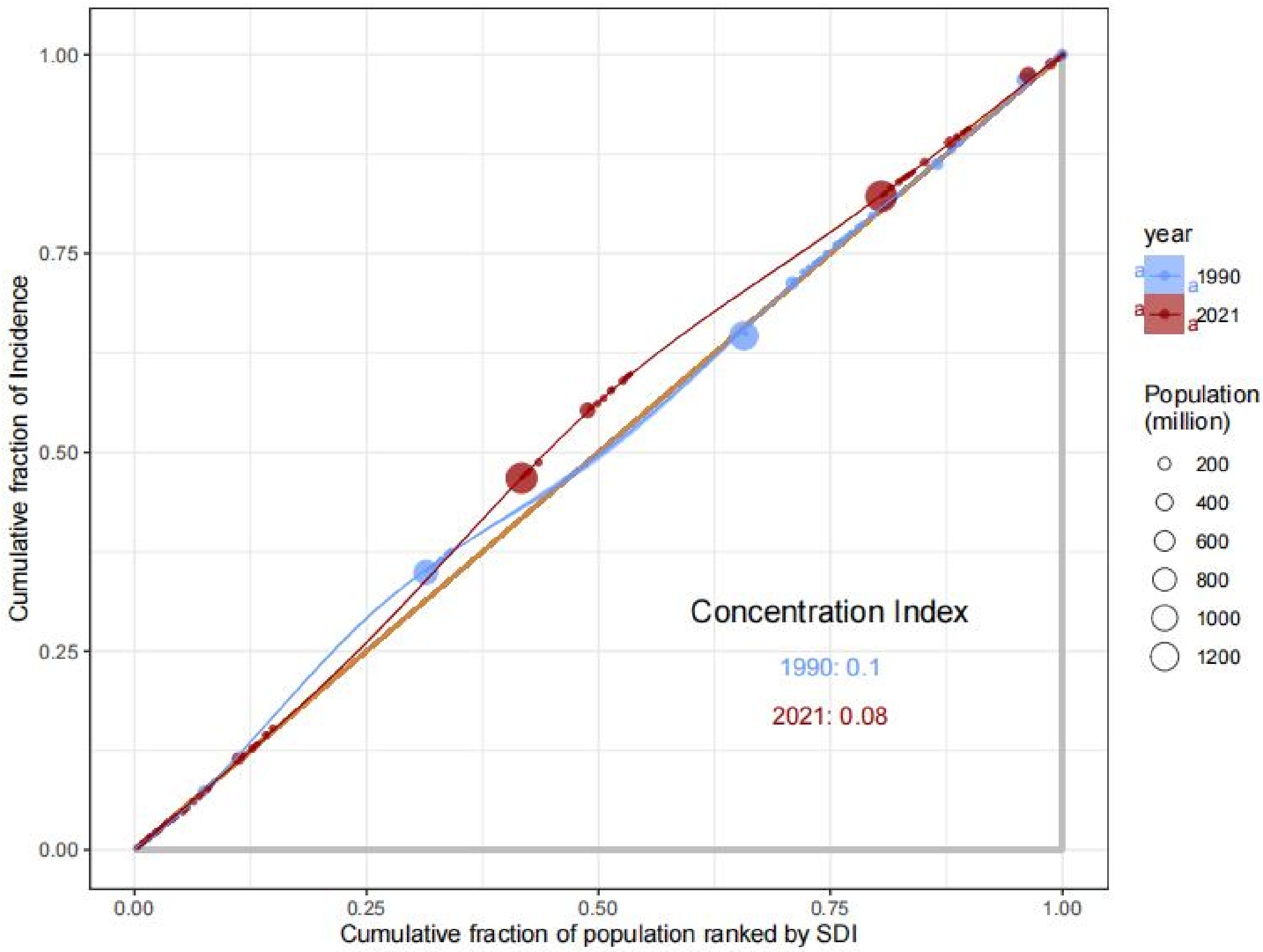

Supplementary Figure 36. **The Slope Index of Inequality (SII) for Migraine Incidence from 1990 to 2021**

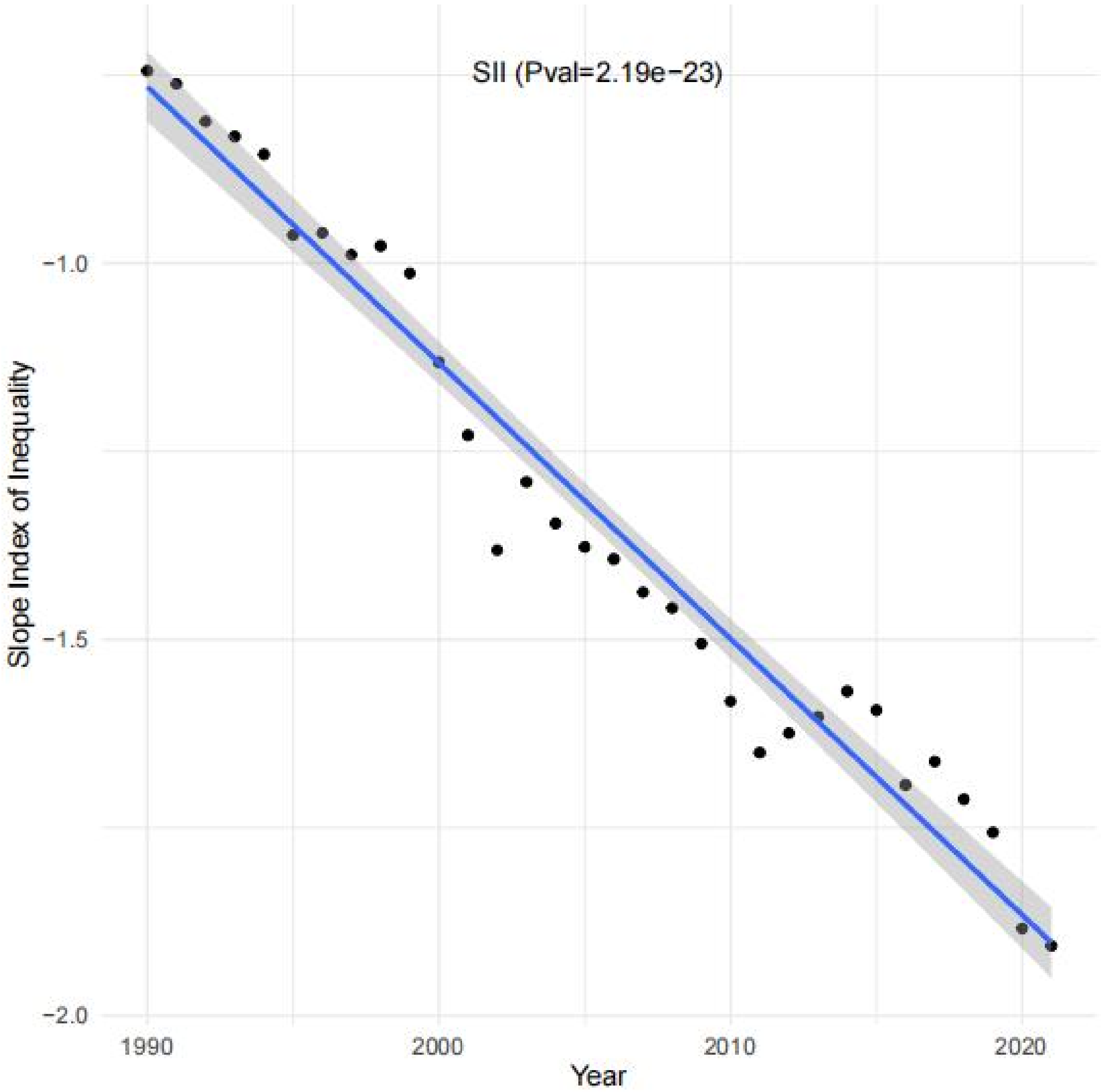

Interpretation:The SII (Slope Index of Inequality) is used to measure the degree of health inequality, with lower values indicating higher levels of inequality.

Supplementary Figure 37. The Crude Incidence of Tension-Type Headache by Socio-demographic Index (SDI) in 1990 and 2021

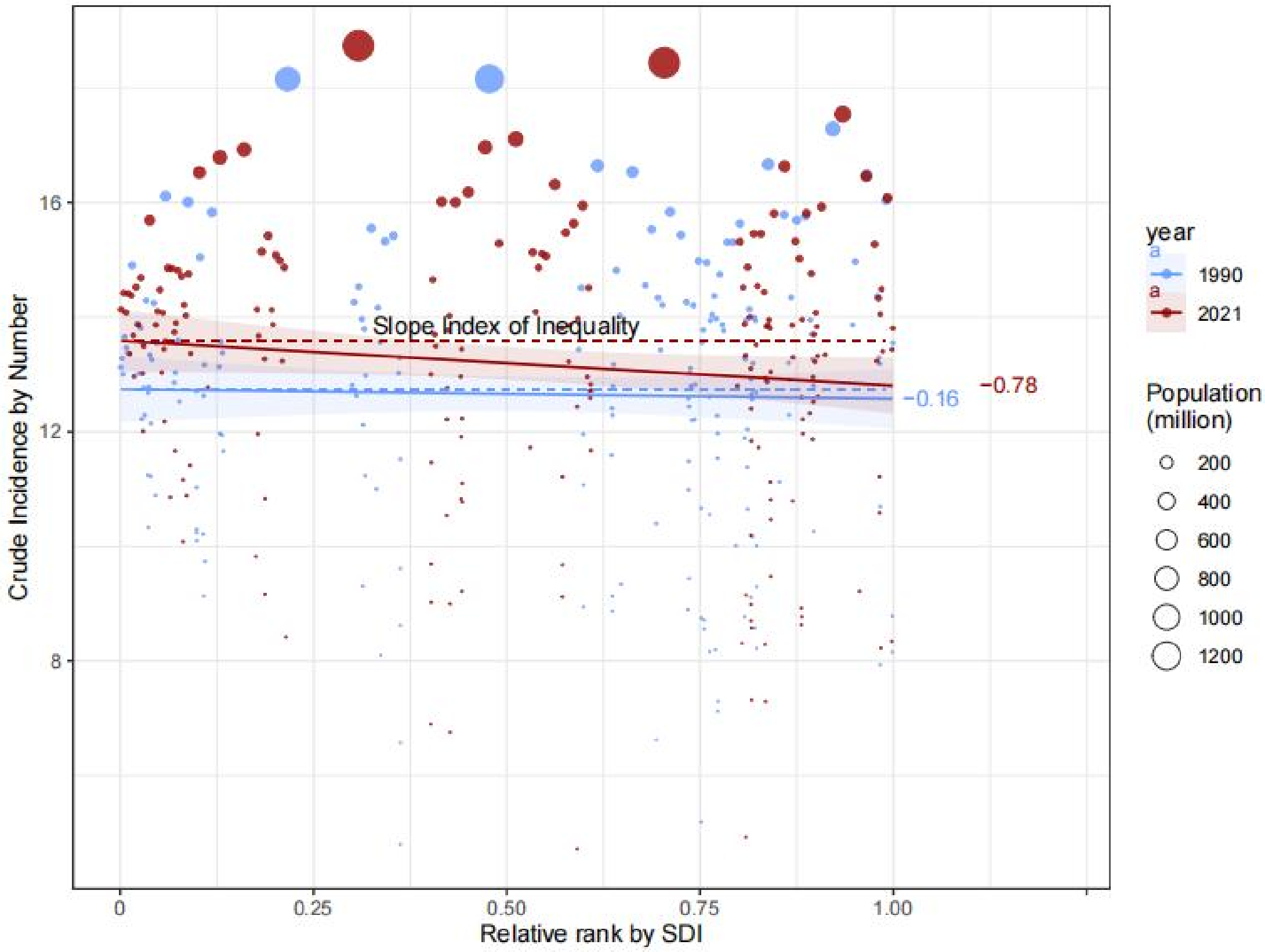

Supplementary Figure 38. The Cumulative Fraction of **Tension-Type Headache** Incidence by Cumulative Fraction of Population Ranked by SDI in 1990 and 2021

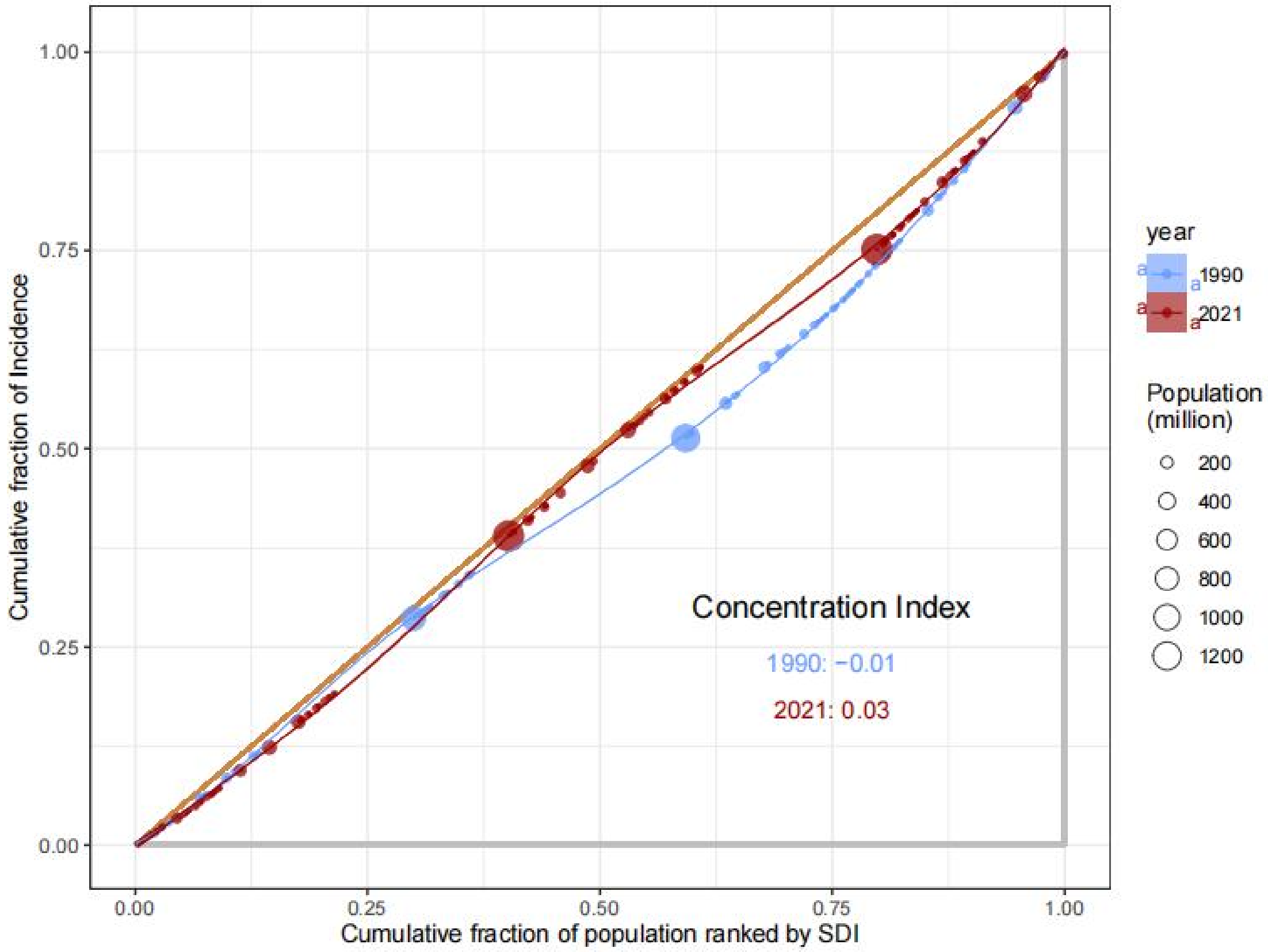

Supplementary Figure 39. The Slope Index of Inequality (SII) for **Tension-Type Headache** Incidence from 1990 to 2021

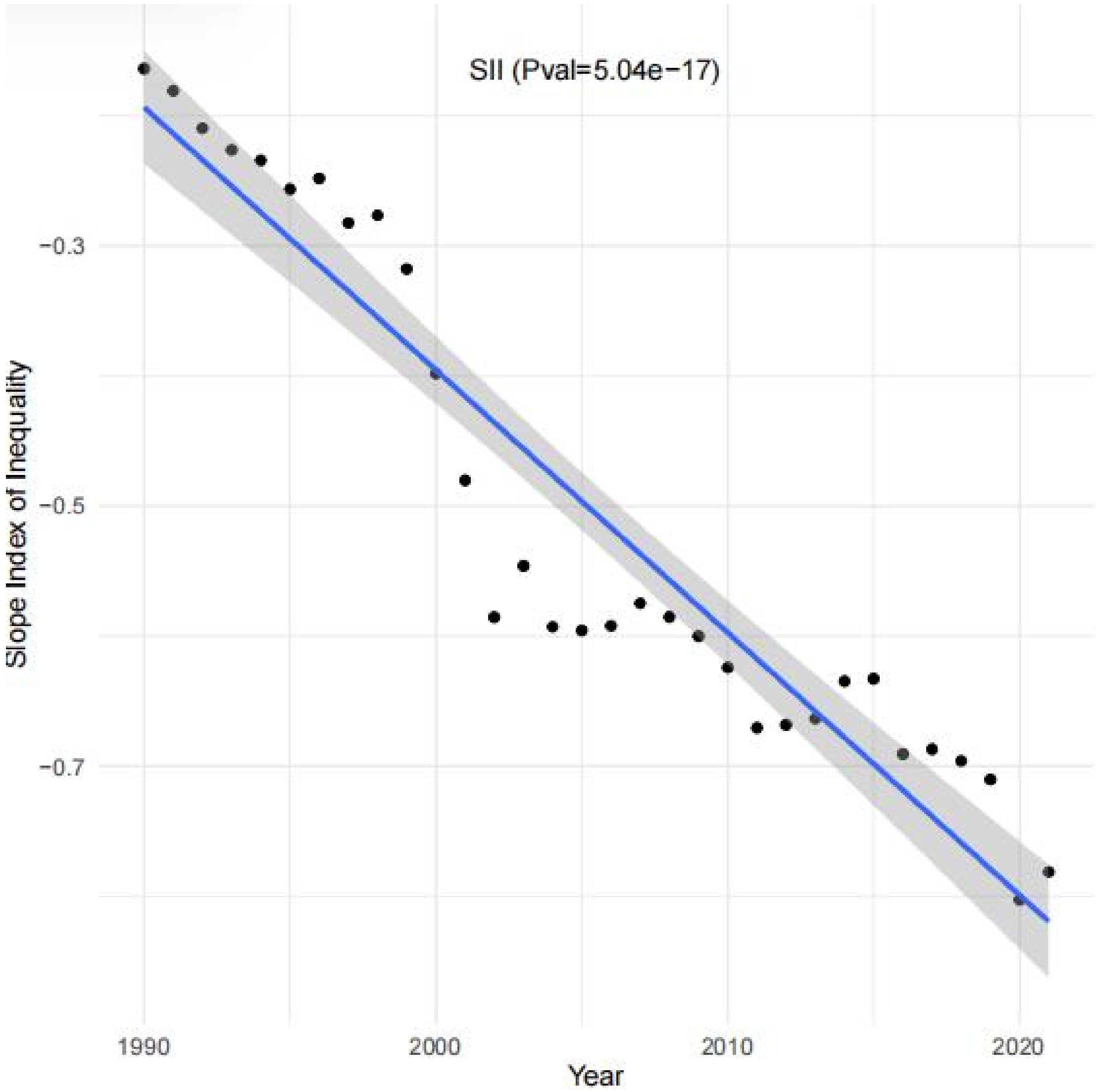

Interpretation:The SII (Slope Index of Inequality) is used to measure the degree of health inequality, with lower values indicating higher levels of inequality.

Supplementary Figure 40. **Changes in Migraine Incidence by Sex from 1990 to 2021 Based on Population-Level Determinants of Aging, Population Growth, and Epidemiological Changes**

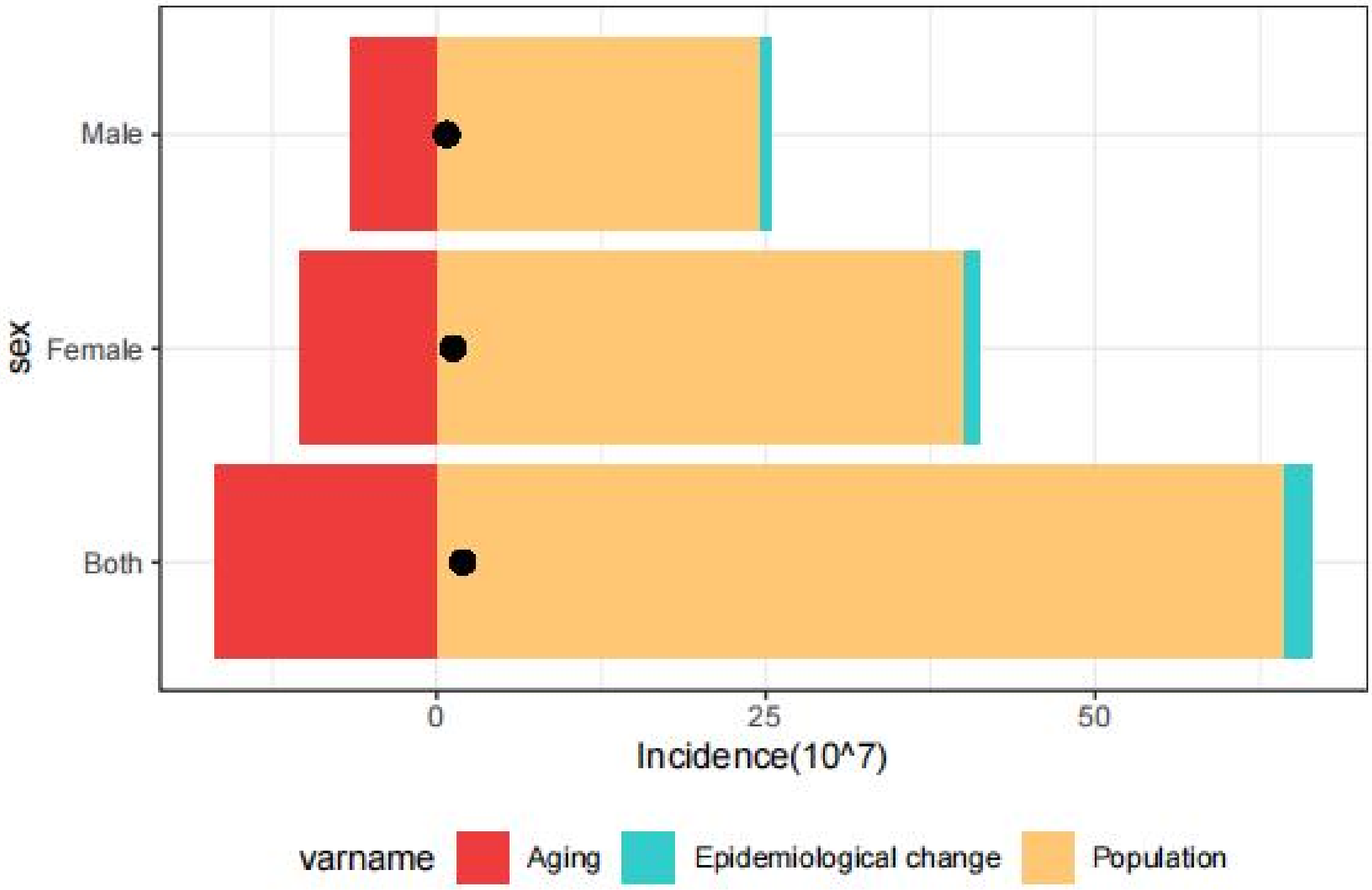

Interpretation:The black dot represents the overall value of change contributed by all three components. For each component, the magnitude of a positive value indicates a corresponding increase in Incidence attributed to that component, while the magnitude of a negative value indicates a corresponding decrease in Incidence attributed to the related component.

Supplementary Figure 41. **Changes in Migraine Prevalence by Sex from 1990 to 2021 Based on Population-Level Determinants of Aging, Population Growth, and Epidemiological Changes**

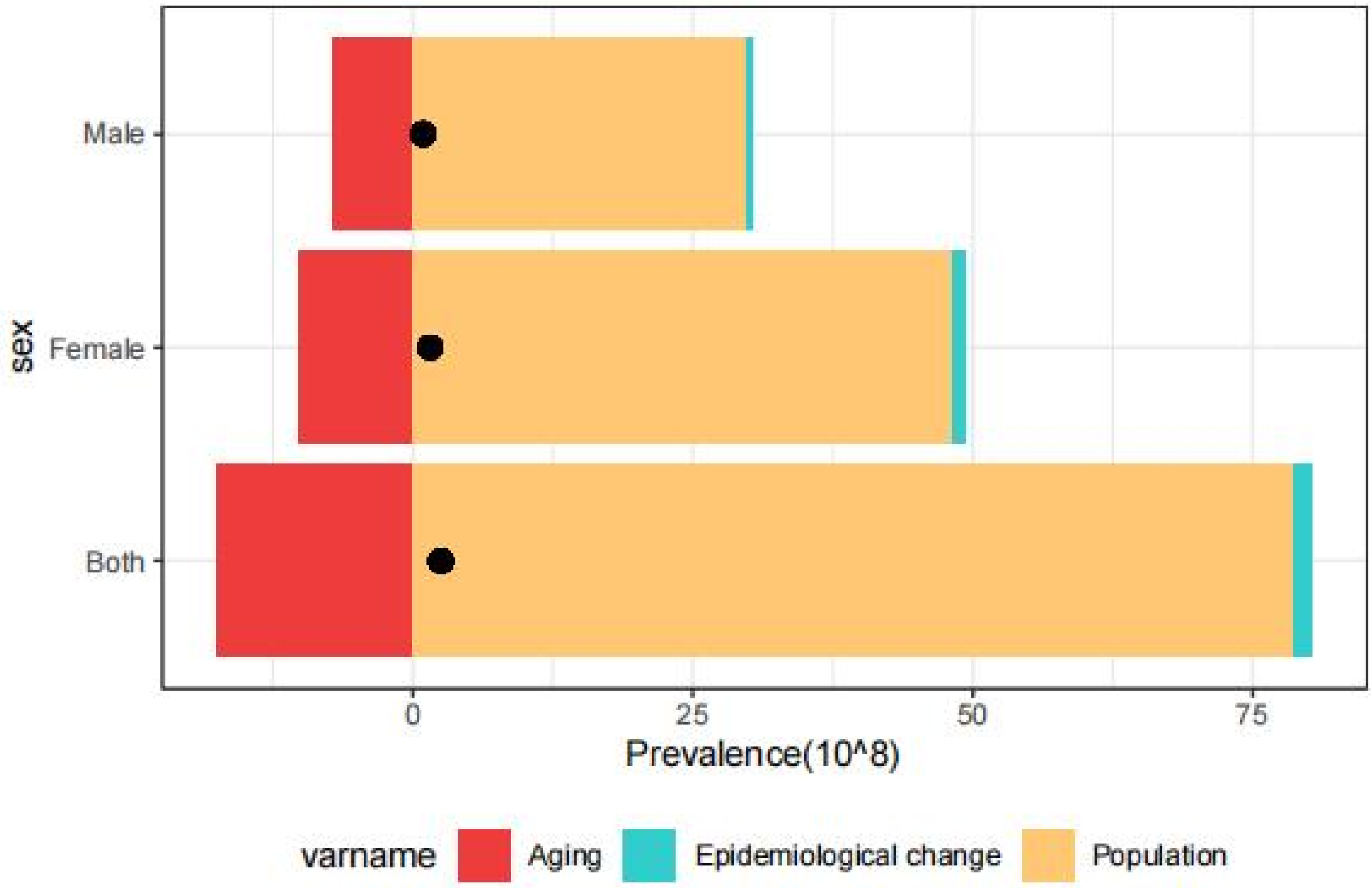

Interpretation:The black dot represents the overall value of change contributed by all three components. For each component, the magnitude of a positive value indicates a corresponding increase in Prevalence attributed to that component, while the magnitude of a negative value indicates a corresponding decrease in Prevalence attributed to the related component.

Supplementary Figure 42. **Changes in Migraine YLDs by Sex from 1990 to 2021 Based on Population-Level Determinants of Aging, Population Growth, and Epidemiological Changes**

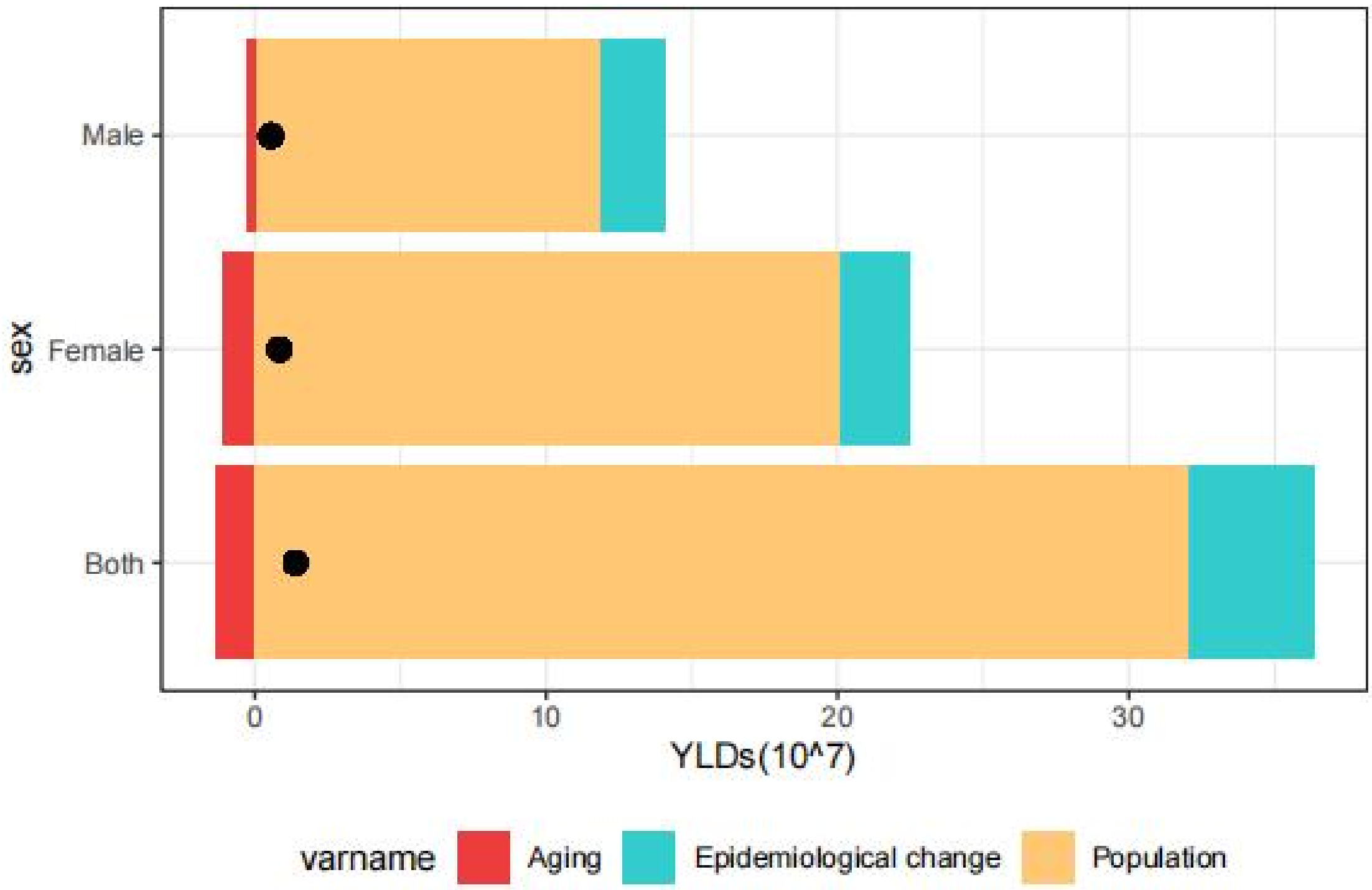

Interpretation:The black dot represents the overall value of change contributed by all three components. For each component, the magnitude of a positive value indicates a corresponding increase in YLDs attributed to that component, while the magnitude of a negative value indicates a corresponding decrease in YLDs attributed to the related component.

Supplementary Figure 43. Changes in **Tension-Type Headache** Incidence by Sex from 1990 to 2021 Based on Population-Level Determinants of Aging, Population Growth, and Epidemiological Changes

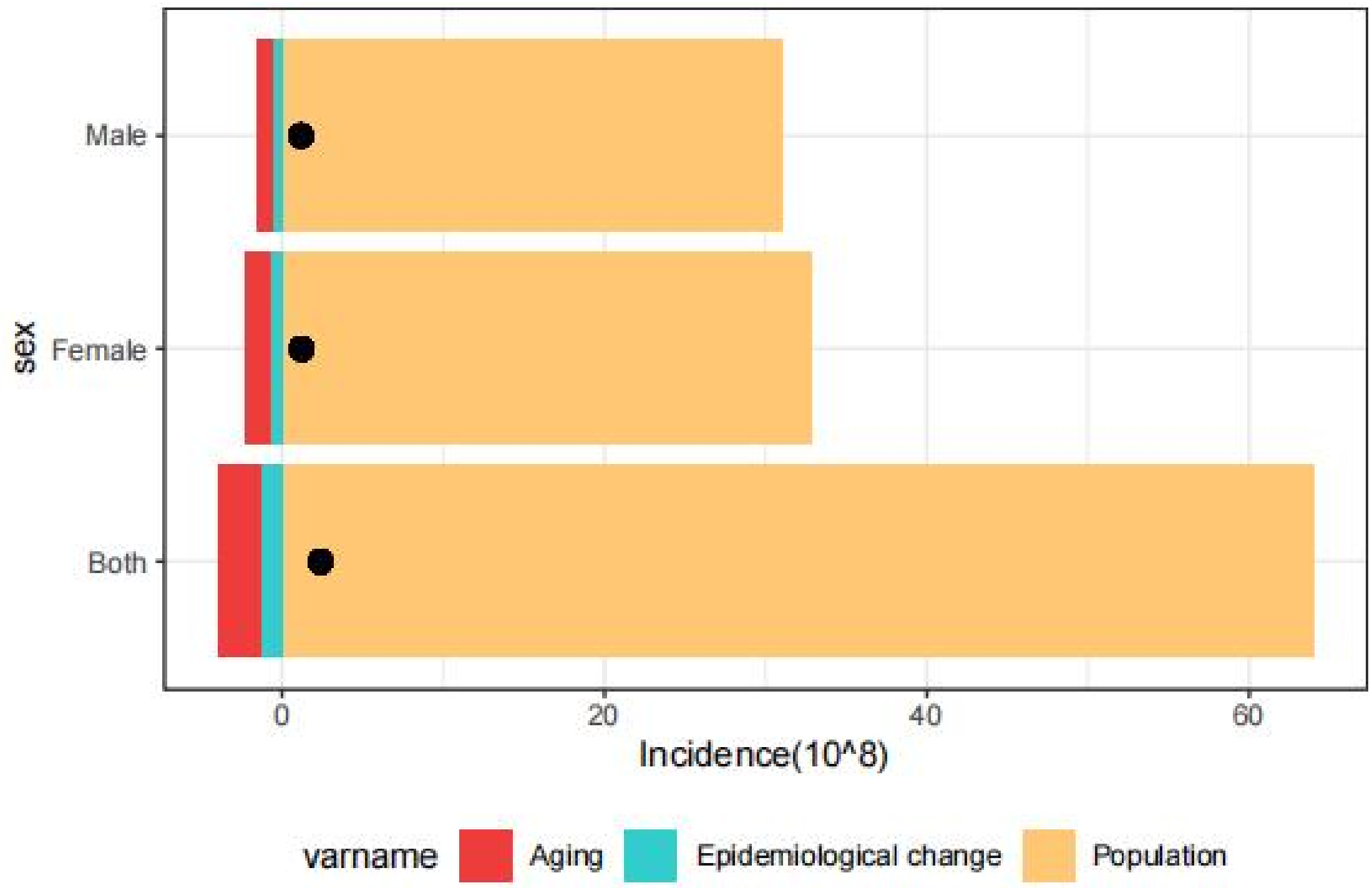

Interpretation:The black dot represents the overall value of change contributed by all three components. For each component, the magnitude of a positive value indicates a corresponding increase in Incidence attributed to that component, while the magnitude of a negative value indicates a corresponding decrease in Incidence attributed to the related component.

Supplementary Figure 44. Changes in **Tension-Type Headache** Prevalence by Sex from 1990 to 2021 Based on Population-Level Determinants of Aging, Population Growth, and Epidemiological Changes

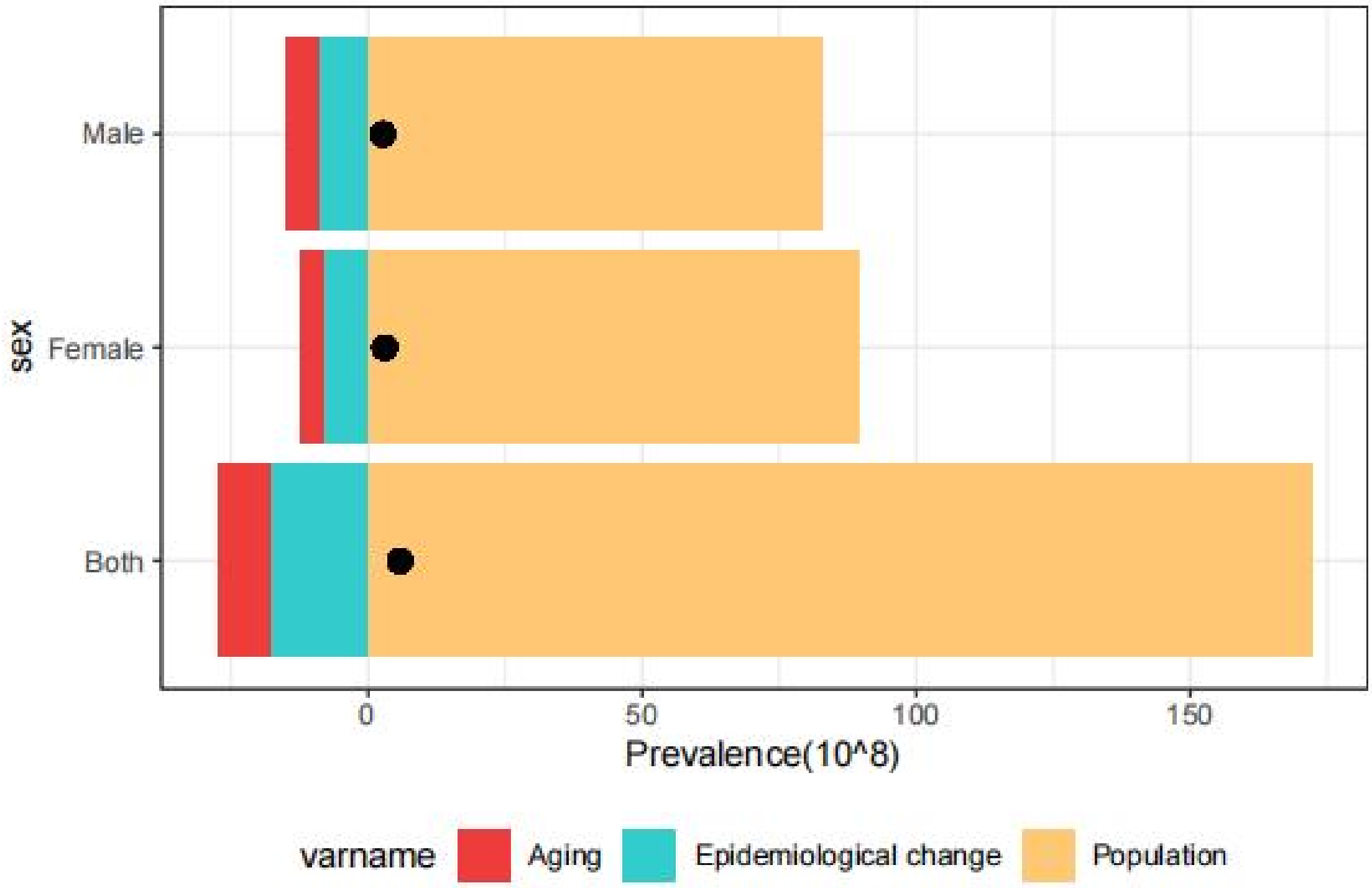

Interpretation:The black dot represents the overall value of change contributed by all three components. For each component, the magnitude of a positive value indicates a corresponding increase in Prevalence **attributed to that component, while the magnitude of a negative value indicates a corresponding decrease in** Prevalence **attributed to the related component.**

Supplementary Figure 45. Changes in **Tension-Type Headache** YLDs by Sex from 1990 to 2021 Based on Population-Level Determinants of Aging, Population Growth, and Epidemiological Changes

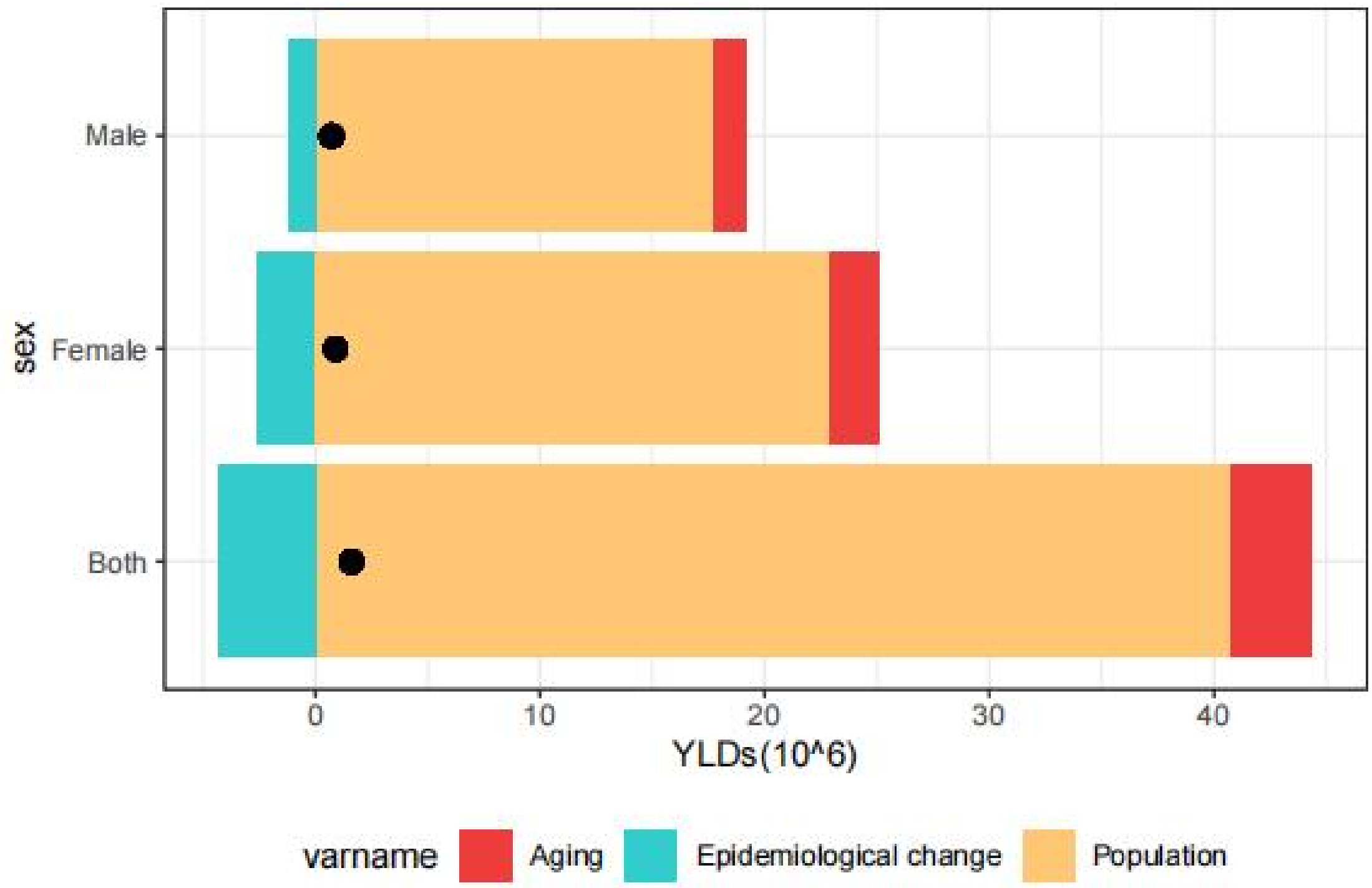

Interpretation:The black dot represents the overall value of change contributed by all three components. For each component, the magnitude of a positive value indicates a corresponding increase in YLDs attributed to that component, while the magnitude of a negative value indicates a corresponding decrease in YLDs attributed to the related component.
