# Supplementary material for: The global burden of migraine and tension-type headache, 1990–2021: a multi-model Bayesian meta-regression with decomposition of age-period-cohort effects, health inequalities, and frontier analysis
Source: Front Neurol. 2026 Aug 18;17:1818932. doi: 10.3389/fneur.2026.1818932 (PMC13529543; doi:10.3389/fneur.2026.1818932)

Supplementary 2 Figure 1. Annual Percentage Change (APC) in the Age-Standardized Prevalence Rates of Migraine

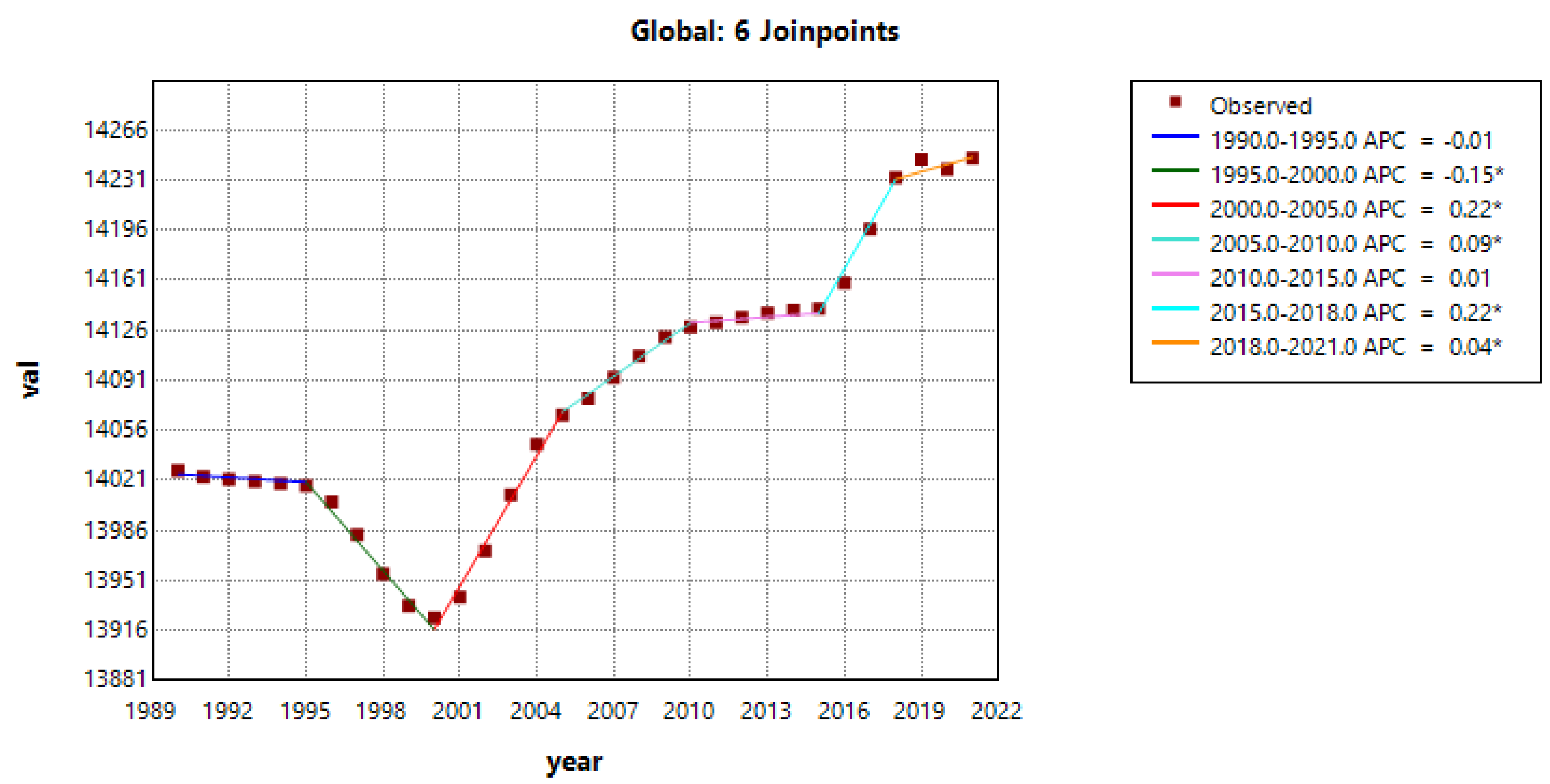

* Indicates that the Annual Percent Change (APC) is significantly different from zero at the alpha = 0.05 level.
Final Selected Model: 6 Joinpoints.

Supplementary 2 Figure 2. Annual Percentage Change (APC) in the Age-Standardized Prevalence Rates of Tension-Type Headache

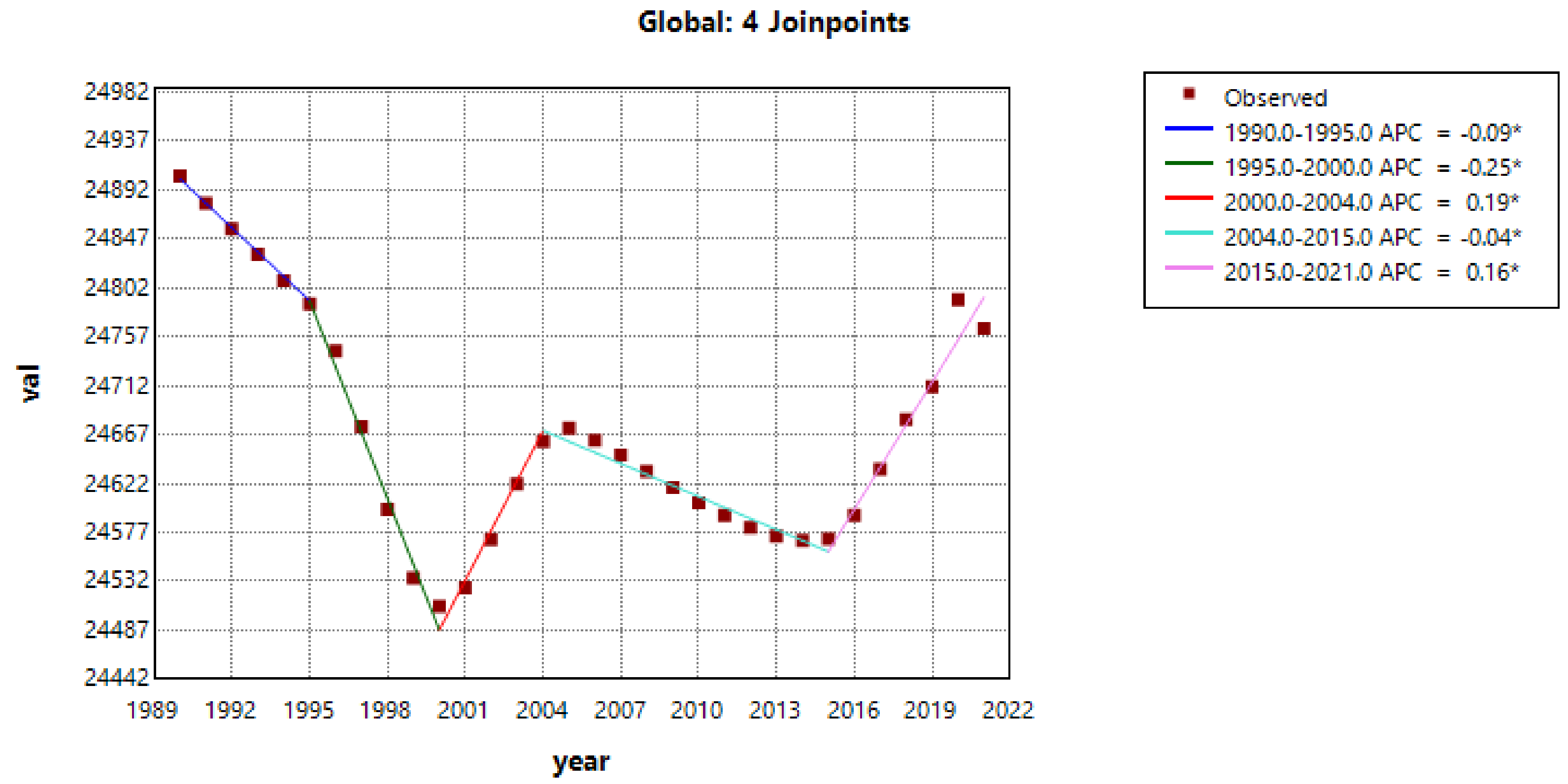

* Indicates that the Annual Percent Change (APC) is significantly different from zero at the alpha = 0.05 level.
Final Selected Model: 4 Joinpoints.

Supplementary 2 Figure 3. Annual Percentage Change (APC) in the Age-Standardized YLDs Rates of Migraine

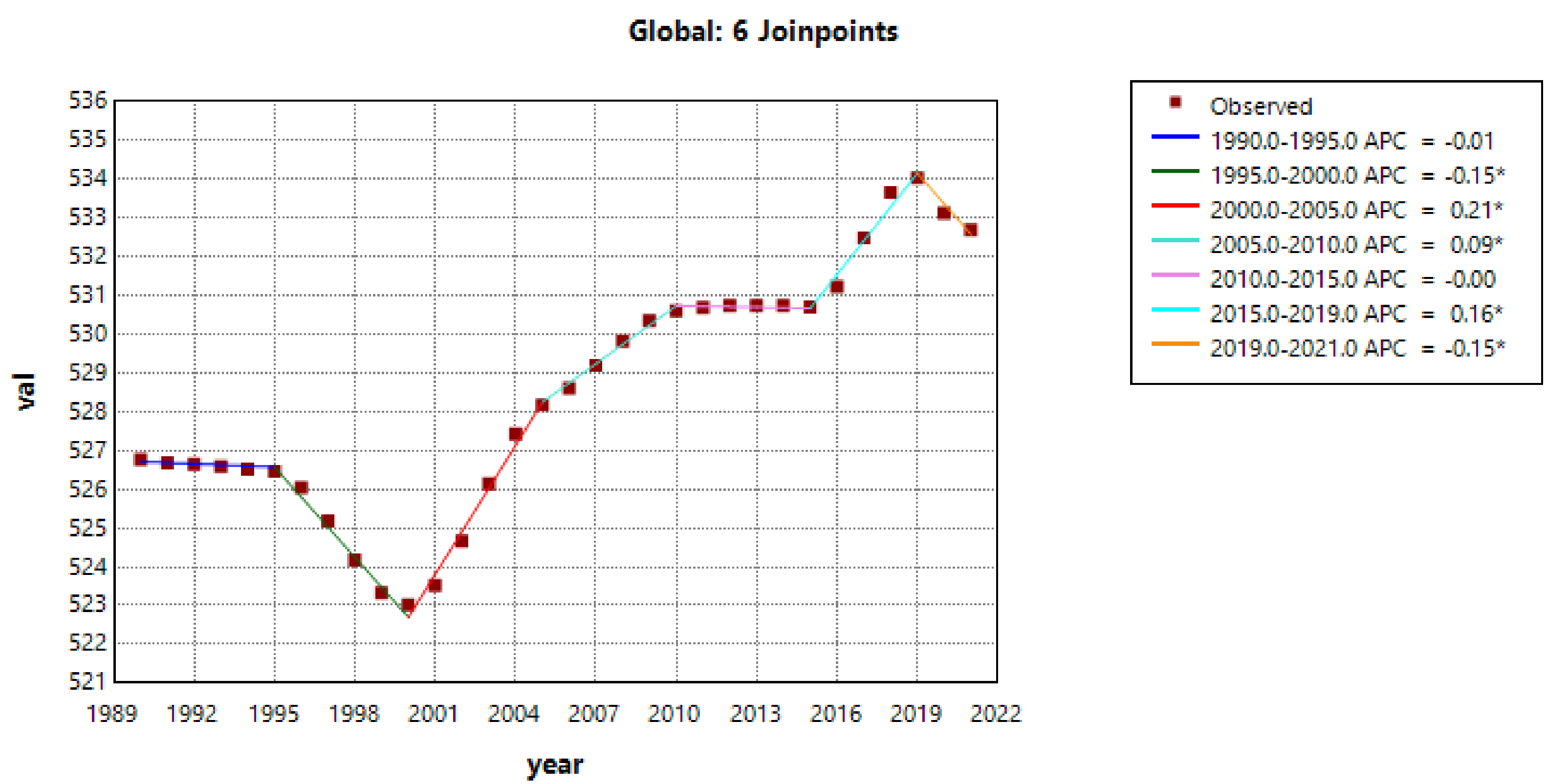

* Indicates that the Annual Percent Change (APC) is significantly different from zero at the alpha = 0.05 level.
Final Selected Model: 6 Joinpoints.

Supplementary 2 Figure 4. Annual Percentage Change (APC) in the Age-Standardized YLDs Rates of Tension-Type Headache

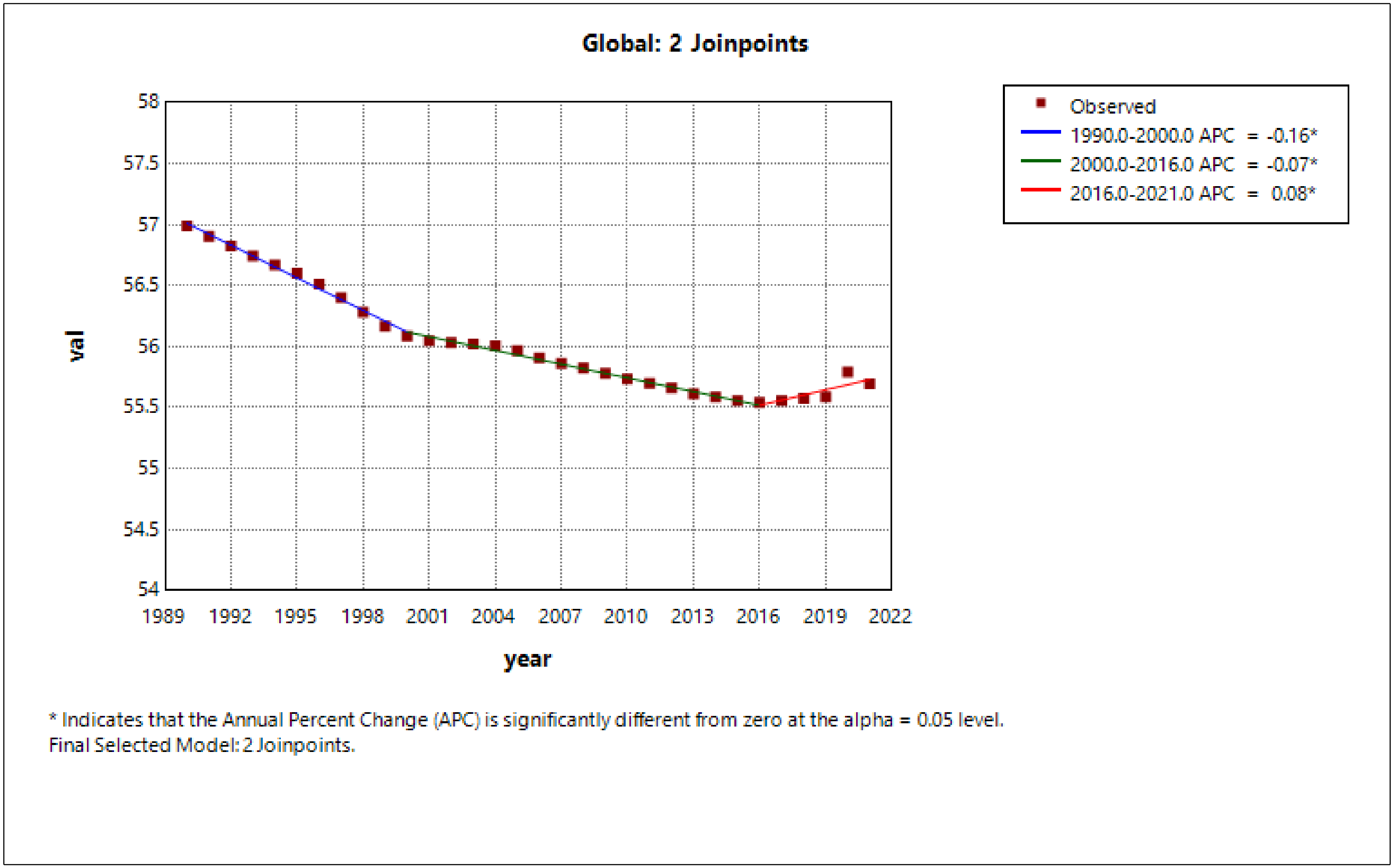

Supplement: Supplementary file 2 [file Supplementary_file_2.pdf]
